# Sonogashira–Hagihara reactions of halogenated glycals

**DOI:** 10.3762/bjoc.8.75

**Published:** 2012-05-02

**Authors:** Dennis C Koester, Daniel B Werz

**Affiliations:** 1 Institut für Organische und Biomolekulare Chemie, Georg-August-Universität Göttingen, Tammannstr. 2, 37077 Göttingen, Germany

**Keywords:** *C*-glycosides, enyne, glycals, reductive/oxidative refunctionalization, Sonogashira–Hagihara reaction

## Abstract

Herein, we report on our findings of the Sonogashira–Hagihara reaction with 1-iodinated and 2-brominated glycals using several aromatic and aliphatic alkynes. This Pd-catalyzed cross-coupling reaction presents a facile access to alkynyl *C*-glycosides and sets the stage for a reductive/oxidative refunctionalization of the enyne moiety to regenerate either *C*-glycosidic structures or pyran derivatives with a substituent in position 2.

## Introduction

**Scheme 1:**
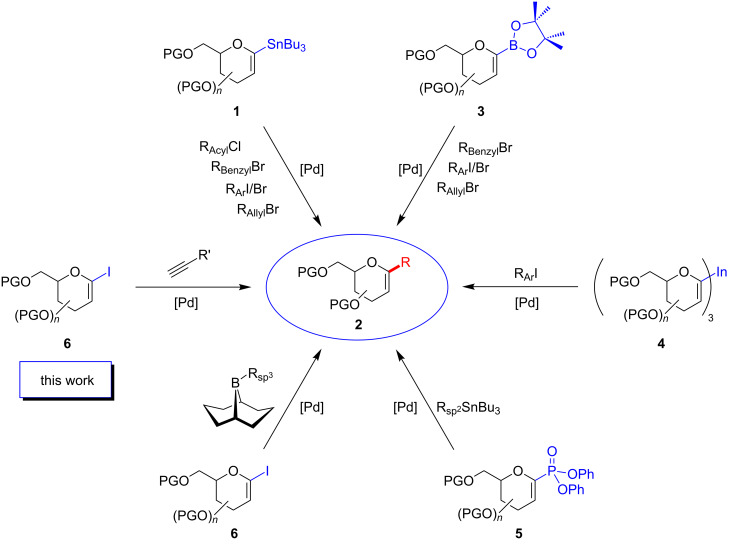
Different strategies to access *C*-glycosides starting from 1-substituted glycals.

Carbohydrates are key players in a plethora of biological processes, such as cell-development, metastasis, cell–cell aggregation and viral infection [1–4]. Many different monosaccharide units and the large variety of possibilities to link two subunits result in an immense variety of highly complex biomolecules [5–7]. In order to mimic certain subunits of oligosaccharides, e.g., for the inhibition of glycosidases or glycosyltransferases, modified mono- or disaccharides have come into the focus of medicinal chemists [8–9]. An important class of carbohydrate mimetics are the *C*-glycosides [10–13]. In such compounds the oxygen of the *O*-glycosidic bond is substituted by a methylene unit rendering them stable to enzymatic degradation or hydrolysis. During the past decades *C*-glycosides have emerged as valuable synthetic targets, not only for medicinal chemists, but also for methodologists [8]. In particular, glycals have transpired as versatile building blocks in the synthesis of various *C*-glycosides. Most of the reported preparations of alkyl- and aryl-*C*-glycosides rely on glycals as starting materials. Transition-metal-catalyzed cross-coupling reactions play a key role in the assembly of these structures; thus, many metalated glycals have been employed in their synthesis. Scheme 1 provides a brief overview.

In 1990 Beau and co-workers utilized 1-stannylated glycals of type **1** in a Stille cross-coupling for the synthesis of aryl-*C*-glycosides such as **2** (R = Ar) [14–15]. To generate these 1-substituted glycals a prefunctionalization of the pseudoanomeric carbon is mandatory. Recent advances in the field of C–H functionalization allowed for the mild and selective borylation of unfunctionalized glycals. Starting from persilylated glycals Ishikawa and Miyaura applied an Ir-catalyzed C–H-functionalization with B_2_pin_2_ to obtain 1-borylated glycals, such as **3**, in excellent yields and selectivity. They elegantly demonstrated the use of these compounds in the synthesis of aryl-, allyl- and benzyl-*C*-glycosides [16]. The group of Minehan described another fascinating approach to aryl-*C*-glycosides in 2003 [17]. They showed that aryl-*C*-glycosides can be prepared from glycalyl indium(III) compounds (e.g., **4**) in a Pd-catalyzed cross-coupling reaction with iodoarenes.

However, it is not only metalated sugar derivatives that have prevailed in the synthesis of *C*-glycosides, but also some electrophilic coupling reagents, such as glycalyl phosphates, and bromo- and iodoglycals. Glycal phosphates of type **5** were employed as electrophiles in a Stille cross-coupling reaction [18]. These building blocks exhibit a high stability and efficiency in their formation and are therefore particularly interesting. In a careful optimization study, Tan and co-workers found that several alkenes hydroborated in situ with 9-BBN can be utilized in a Suzuki–Miyaura coupling with iodoglucals **6** to yield aliphatic *C*-glycosides [19]. This method impressively demonstrates the power of the Suzuki–Miyaura coupling in the formation of C(sp^2^)–C(sp^3^) bonds for the preparation of carbohydrate mimetics. Friesen and co-workers reported on a synthesis of aryl-*C*-glycosides employing different metalated arenes with persilylated 1-iodoglucals [20]. These proved to be particularly reactive to organozinc and organoboron compounds, whereas Hayashi successfully disclosed an approach to react simple stannylated or even electron-poor olefins with 2-bromoglucals in Stille and Heck reactions, respectively [21]. The dienes obtained during these transformations were successfully converted in Diels–Alder reactions to afford carbocyclic chiral compounds with a sugar backbone.

In 2008, Gagné introduced a Ni-mediated Negishi coupling to synthesize alkyl- and aryl-*C*-glycosides from glycosyl halides [22]. Worthy of note is that this transformation displays a C(sp^3^)–C(sp^3^) coupling in the case of alkyl-*C*-glycosides and a C(sp^2^)–C(sp^3^) coupling in the preparation of aryl-*C-*glycosides. Although the diasteroselectivity of the reaction is highly dependent on the type of monosaccharide, this approach is unique due to its use of fully functionalized sugars, thus avoiding further refunctionalization steps to obtain the native *C*-glycoside.

## Results and Discussion

Despite the numerous organometallic reactions performed with substituted glycals, to the best of our knowledge there has been no in-depth study of the synthesis of 1- and 2-alkynylated glycals by means of the Sonogashira–Hagihara reaction to date. Herein, we wish to report on our findings with respect to the reaction of persilylated 1-iodoglucal **7** and peracetylated 2-bromogalactal **10** with readily available alkynes **8a**–**8h**. We became interested in this chemistry whilst searching for a method to synthesize (1→6)-linked *C*-glycosidic disaccharide mimetics [23]. The simplicity of the transformation, the mild reaction conditions, and the commercial availability of the catalyst render this method highly useful for the preparation of internal alkynes [24].

We started our investigations with the persilylated 1-iodoglucal **7**, which is easily available by a sequence of lithiation and iodination from the parent, fully TIPS-protected congener [25]. The Sonogashira reaction was carried out under standard conditions. Pd(PPh_3_)_2_Cl_2_ was used as catalyst, and CuI served as cocatalyst. The reaction was performed at room temperature for 12 h in neat triethylamine acting as the solvent as well as base. As coupling partners, a variety of commercially available alkynes as depicted in Table 1 were investigated. The yields proved to be good to excellent (72% to 92%). Aromatic as well as aliphatic alkynes could be employed for this reaction leading to excellent results. Fluorinated alkynyl-*C*-glycosides (Table 1, entry 4) may be readily functionalized at the aromatic core, whereas TMS-protected enynes (Table 1, entry 6) allow for further manipulations on the alkynyl residue. Our previous studies also revealed that even carbohydrate-derived alkynes can be utilized under these reaction conditions in an efficient manner [23].

**Table 1:** Sonogashira–Hagihara reactions of 1-iodoglucal **7** with different alkynes **8a**–**8h**.

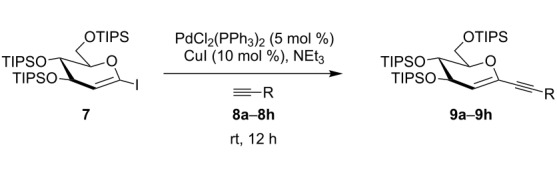			

entry	alkyne	product	yield [%]^a^

1	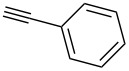 **8a**	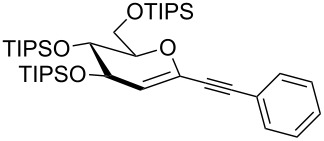 **9a**	92
2	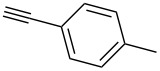 **8b**	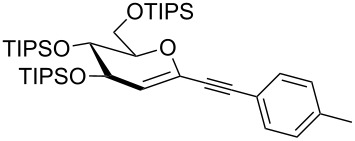 **9b**	72
3	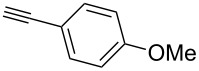 **8c**	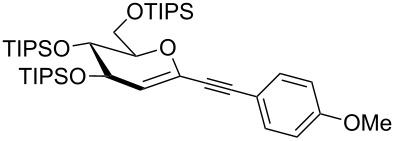 **9c**	86
4	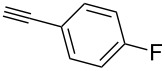 **8d**	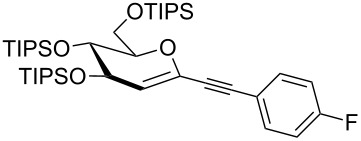 **9d**	82
5	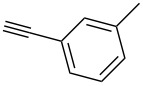 **8e**	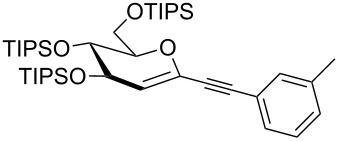 **9e**	90
6	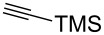 **8f**	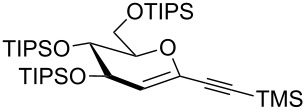 **9f**	79
7	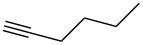 **8g**	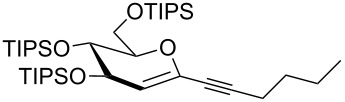 **9g**	83
8	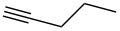 **8h**	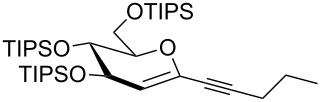 **9h**	90

^a^Isolated yields.

Our recent interest in domino reactions starting with 2-brominated glycals [26–28] motivated us to investigate also the behaviour of peracetylated 2-bromogalactal **10** in Sonogashira–Hagihara reactions (Table 2). We found that **10** is much less reactive than **7**. This may be due to a more facile oxidative addition to the C–I bond compared to the C–Br bond. Furthermore, we assume that the electron density at C-2 of the sugar core is particularly high, rendering the 2-palladated glycal a bad electrophile towards electron-poor or electron-neutral alkynes. To push the reaction an elevated temperature was necessary, and only moderate yields were obtained.

**Table 2:** Sonogashira–Hagihara reactions of 2-bromogalactal **10** with different alkynes.

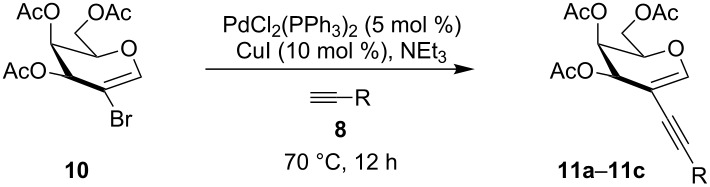			

entry	alkyne	product	yield [%]^a^

1	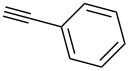 **8a**	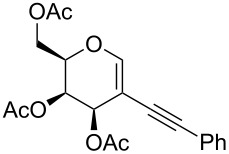 **11a**	66
2	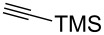 **8f**	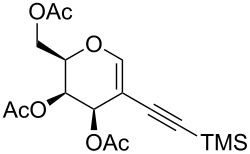 **11b**	45
3	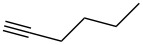 **8g**	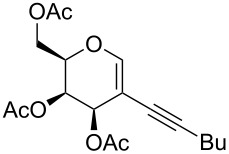 **11c**	traces

^a^Isolated yields.

**Scheme 2:**
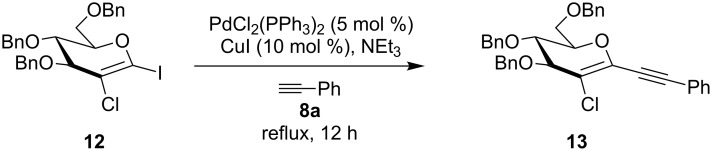
Sonogashira–Hagihara reaction of 1-iodo-2-chloroglucal **12** with phenylacetylene (**8a**) to afford **13**.

Efforts to react perbenzylated 2-chloro-1-iodoglucal **12** in a twofold Sonogashira reaction with an excess of phenylacetylene resulted in a chemoselective monoalkynylation of the pseudoanomeric position in quantitative yield (Scheme 2). Even the use of an elevated temperature did not lead to the formation of an enediyne. Further refunctionalization of the enynes was achieved by selective reduction of the triple bond by making use of Raney nickel (Table 3). We found that the electron-rich enol ether moiety remains untouched, when reaction times of less than four hours were chosen in the case of the enynes **9e**–**9h**. It should be noted that methanol was a crucial part of the solvent mixture, otherwise no reaction was observed. Interestingly, when we employed the perbenzylated enyne **9ea** the yield of the alkyne-reduced product decreased tremendously. A mixture of completely reduced products was obtained. Thus, we assume that not only electronic effects, but also the sterically encumbered TIPS groups render the reduction of the olefinic moiety much more difficult.

**Table 3:** Reduction of carbohydrate-derived enynes **9e**–**9h** and **11a**–**11b**.

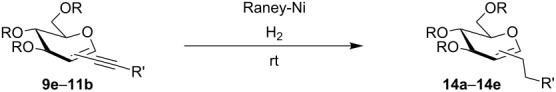					

entry	substrate	time [h]	solvent	product	yield [%]^a^

1	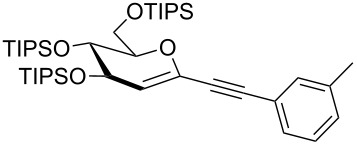 **9e**	3	MeOH:THF (1:1)	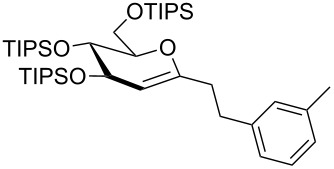 **14a**	88
2	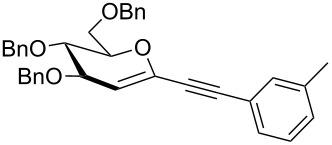 **9ea**	12	MeOH:THF (1:1)	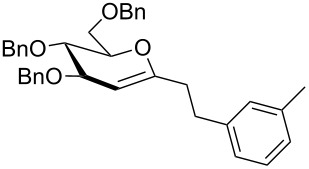 **14b**	11
3	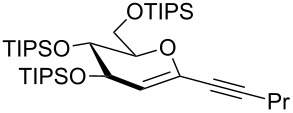 **9h**	4	MeOH:THF (1:1)	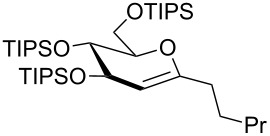 **14c**	63
4	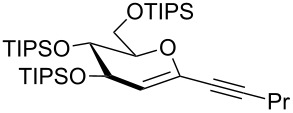 **9e**	4	THF	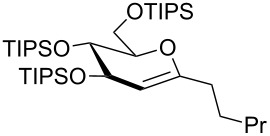 **14c**	0
5	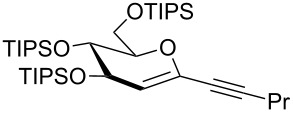 **9e**	7	MeOH:THF (1:1)	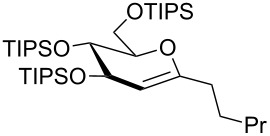 **14c**	58
6^b,c^	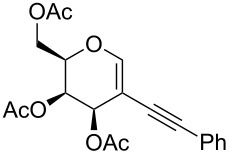 **11a**	12	MeOH:DCM:EtOAc (3:1:1)	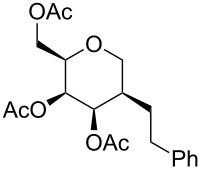 **14d**	97
7^b^	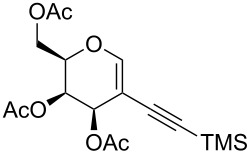 **11b**	12	MeOH:DCM:EtOAc (3:1:1)	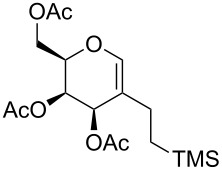 **14e**	86

^a^Isolated yields; ^b^Pearlman’s catalyst (Pd(OH)_2_/C) was used instead of Raney-Ni; ^c^Fully reduced carbohydrate mimetic was observed; a final proof of the stereochemistry by NOESY effects was not possible due to strongly overlapping signals. However, the stereochemistry given is highly reasonable because of the shielding of the top face by three substituents [29].

In contrast to the other enynes the carbohydrate derivative **11a** was fully reduced in a diastereoselective manner and in excellent yield to the pyran **14d** by employing Pearlman’s catalyst (rt, overnight), whereas in the case of enyne **11b**, under the same reaction conditions, only the triple bond was reduced to furnish enol ether **14e** selectively.

In three cases we further functionalized the 1-alkylated glycals by an epoxidation/epoxide-opening sequence [30–33]. Dimethyldioxirane (DMDO) was used as a neutral epoxidation reagent leading to a facial-selective epoxide formation [34–36]. The so-obtained highly reactive acetal epoxide was either attacked by a superhydride, such as LiBHEt_3_ [31], or by a Lewis acidic hydrogen transfer agent, such as DIBAL-H [32–33]. In the former case, an S_N_2-type reaction takes place leading to the α-*gluco*-configured *C*-glycoside **15a** in a moderate yield of 30% (Table 4). The aluminium centre coordinates the epoxide oxygen allowing the hydride to attack from the same side, leading to β-configured alkyl-*C*-glycosides. The epoxidation/ring-opening sequence of TIPS-protected glucals proved to be challenging; for the respective product **15c** only a yield of 13% was observed.

**Table 4:** Diastereoselective epoxidation/epoxide opening sequence employing different hydride sources to afford **15a**–**15c**.

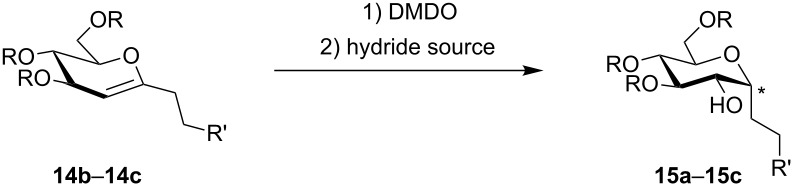			

entry	substrate	product	yield [%]^a^

1^b^	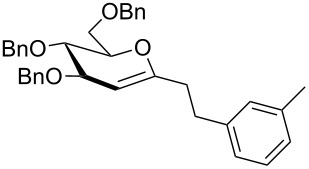 **14b**	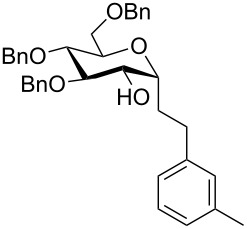 **15a**	30
2^c^	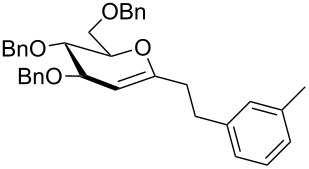 **14b**	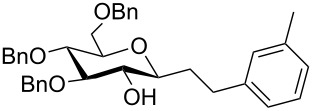 **15b**	40
3^c^	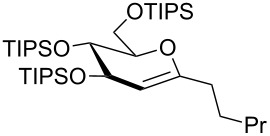 **14c**	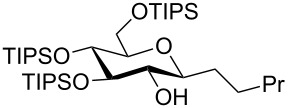 **15c**	13

*Stereochemistry depending on the hydride source; ^a^Isolated yields; ^b^LiBHEt_3_ was employed as a hydride source; ^c^DIBAL-H was employed as a hydride source.

## Conclusion

We investigated the behaviour of 1-iodinated and 2-brominated glycals in Sonogashira–Hagihara cross-coupling reactions with various alkylated and arylated alkynes. 1-Alkynylated glycals were obtained in very good yields whereas the alkynylation in position 2 gave poorer results. Chemoselective reduction of the triple bond in the resulting enyne system by the action of Raney-Ni furnished enol ethers, which could be readily refunctionalized. Methanol proved to be an essential co-solvent in order to execute the Ni-catalyzed reduction. The enol ether double bond could be further hydroxylated by an epoxidation/epoxide opening sequence. Depending on the hydride source α- and β-configured alkyl-*C*-glycosides were obtained diastereoselectively in moderate yield. These investigations with respect to the Sonogashira–Hagihara coupling complement the rich organometallic chemistry that has already been performed with borylated, stannylated and phosphorylated glycals.

## Supporting Information

Supporting Information containing all experimental details and analytical data of all new compounds given in this article as well as their ^1^H and ^13^C NMR spectra is provided.

File 1 Experimental procedures, analytical data and NMR spectra.
