# Supplementary material for: Sonogashira–Hagihara reactions of halogenated glycals
Source: Beilstein J Org Chem. 2012 May 2;8:675–82. doi: 10.3762/bjoc.8.75 (PMC3388853; doi:10.3762/bjoc.8.75)
Supplement: File 1 — Experimental procedures, analytical data and NMR spectra. [file Beilstein_J_Org_Chem-08-675-s001.pdf]

Supporting Information
for
Sonogashira–Hagihara reactions of halogenated glycals

Dennis C. Koester and Daniel B. Werz*

Address: Institut für Organische und Biomolekulare Chemie, Georg-August-Universität Göttingen, Tammannstr. 2, 37077 Göttingen, Germany

Email: Daniel B. Werz* - dwerz@gwdg.de

* Corresponding author

Experimental procedures, analytical data and NMR spectra

Table of contents

General experimental S2

General procedures S2

Syntheses and analytical data of new compounds S4

^{1}H and ^{13}C NMR spectra of new compounds S28

General experimental

All solvents were distilled before use, unless otherwise stated. Tetrahydrofuran (THF) and diethyl ether (Et_2O) were distilled from sodium/benzophenone under a nitrogen atmosphere. Dichloromethane (CH_2Cl_2) and toluene were distilled from $CaCl_2$ under a nitrogen atmosphere. Air and moisture sensitive reactions were carried out in oven-dried or flame-dried glassware, septum-capped under atmospheric pressure of argon. Commercially available compounds were used without further purification, unless otherwise stated. A microwave oven with adjustable power in the range of 0–400 W was used to apply microwave irradiation. The exact reaction conditions are given in the respective procedures. Proton (^{1}H) and carbon (^{13}C) NMR spectra were recorded on a 300, 500 or 600 MHz instrument using the residual signals from $CHCl_3$, δ 7.26 ppm and δ 77.0 ppm, DMSO, δ 2.54 ppm and δ 40.45 ppm, acetone, δ 2.09 ppm and δ 30.92 ppm, δ 207.07 ppm, and methanol, δ 4.87 ppm and δ 49.2 ppm, as internal references for ^{1}H and ^{13}C chemical shifts, respectively. Assignments of the respective signals were made by the combination of H,H-COSY, HSQC and HMBC experiments. ESI-HRMS mass spectrometry was carried out on a FTICR instrument. IR spectra were measured by using a conventional spectrometer. UV spectra were measured with a common photometer. Optical rotations were measured at 20 °C by using a common optical-rotation instrument.

General procedures

a) Sonogashira–Hagihara reactions

The organic halide (1.0 equiv) and the respective alkyne (1.0–2.0 equiv) were dissolved in NEt_3 (30 mL/mmol). Dichlorobistriphenylphosphane-palladium(II) (5 mol %) and copper iodide (10 mol %) were subsequently added to the reaction mixture. The mixture was stirred for 12 h at room temperature. The reaction mixture was diluted with water and the aqueous phase was re-extracted with chloroform (3×). The combined organic layers were dried over Na_2SO_4, filtered and concentrated by rotary evaporation. The crude product was purified by column chromatography on silica gel.

b) Changing the protecting groups from TIPS to Bn

ba) Deprotection of TIPS groups

The silylated substrate (1.0 equiv) was dissolved in THF (25 mL/mmol). A solution of $TBAF \cdot 3H_2O$ (1.5 equiv per silyl group) in THF (1.7 mL/mmol) was added dropwise.

The reaction was stirred at room temperature overnight. The solvent was removed by rotary evaporation and the crude material was subjected to a column chromatography on silica gel.

bb) Protection with Bn groups

The respective substrate (1.0 equiv) was dissolved in DMF (12 mL/mmol). The solution was cooled to 0 °C and NaH (1.5 equiv per hydroxy group) was added in one portion. The reaction mixture was stirred for 30 min at 0 °C before BnBr (1.5 equiv per hydroxy group) was added dropwise. The reaction was allowed to warm to room temperature. After stirring overnight, the reaction was finished by the addition of water. The aqueous layer was extracted with EtOAc (3×). The combined organic layers were washed with sat. aq. NaCl solution (2×). The organic layer was dried over Na_2SO_4, filtered and concentrated by rotary evaporation. The crude product was purified by column chromatography on silica gel.

c) Reductions

ca) Reduction by Raney-Ni

A slurry of Raney-Ni was washed with water (4×). When pH 7 was reached it was further washed with MeOH (4×). A solution of the substrate (1.0 equiv) in MeOH:THF (1:1) was added to the washed slurry of Raney-Ni. The argon atmosphere was replaced by hydrogen and the reaction mixture was stirred for 3–5 h. Raney nickel was removed by filtration through a pad of celite after the reaction. The filtrate was concentrated and purified by column chromatography on silica gel.

cb) Reduction by Pearlman’s catalyst

The substrate (1.0 equiv) was dissolved in a mixture of MeOH:CH_2Cl_2:EtOAc (3:1:1). Pearlman’s catalyst ($Pd(OH)_2/C$) was added to the reaction mixture. The argon atmosphere was replaced by hydrogen and the reaction mixture was stirred at room temperature overnight. The catalyst was removed by filtration through a pad of celite and the filtrate was concentrated. The crude mixture was purified by column chromatography on silica gel.

1-Phenylethynyl-3,4,6-tri-*O*-isopropylsilylglucal (9a)

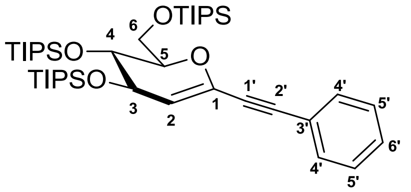

The reaction was performed according to general procedure **a**. 1-Iodoglucal **7** (1.00 g, 1.35 mmol, 1.0 equiv) and phenylacetylene (275.9 mg, 297 µL, 2.70 mmol, 2.0 equiv) were dissolved in NEt_3 (40 mL). Bis(triphenylphosphine)palladium(II) dichloride (47.4 mg, 0.067 mmol, 0.05 equiv) and copper iodide (25.7 mg, 0.135 mmol, 0.1 equiv) were added subsequently . After column chromatography on silica gel (pentane/CH_2Cl_2 = 12:1 → pentane/ CH_2Cl_2 = 6:1) the desired compound **9a** was obtained as a yellow oil (890 mg, 92%).

Analytical data of compound **9a**:

R_f: 0.21 (hexane/CH_2Cl_2 = 10:1).

$[\alpha]_D^{20}$ = –20.2° (c = 0.51, $CHCl_3$).

^{1}H-NMR (300 MHz, $CDCl_3$): δ = 0.96–1.12 (m, 63H, TIPS-H), 3.86–3.92 (dd, *J* = 4.6, 11.3 Hz, 1H, 6-H), 4.01–4.10 (m, 3H, 3-H, 5-H, 6-H'), 4.30–4.37 (m, 1H, 4-H), 5.32 (dd, *J* = 5.4, 1.7 Hz, 1H, 2-H), 7.28–7.30 (m, 3H, 5'-H, 6'-H), 7.45–7.49 (m, 2H, 4'-H).

^{13}C-NMR (125 MHz, $CDCl_3$): δ = 12.1, 12.3, 12.5 (CH-TIPS), 18.0, 18.1, 18.2 (CH_3-TIPS), 61.7 (C-6), 66.0 (C-3), 69.5 (C-5), 81.54 (C-4), 85.1 (C-1'), 87.1 (C-2'), 107.1 (C-2), 122.4 (C-3'), 128.2 (C-5'), 128.5 (C-6'), 131.8 (C-4'), 136.2 (C-1).

IR (film): ν (cm^{-1}) = 2757, 2724, 2620, 2562, 2225, 1946, 1877, 1550.

MS (ESI): *m*/*z* (%) = 715.50 (100) $[M+H]^+$.

$C_{11}H_{74}O_4Si_3$ (715.28) calc.: 715.49739

found: 715.49641, $[M+H]^+$ (ESI-HRMS).

1-(4-Methylphenylethynyl)-3,4,6-tri-*O*-isopropylsilylglucal (9b)

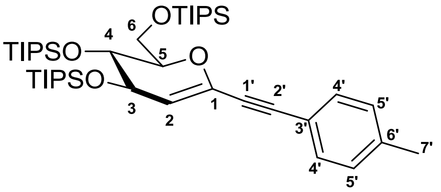

The reaction was performed according to general procedure **a**. 1-Iodoglucal **7** (100 mg, 0.135 mmol, 1.0 equiv) and 1-ethynyl-4-methylbenzene (31.4 mg, 0.270 mmol, 2.0 equiv) were dissolved in NEt_3 (4 mL). Bis(triphenylphosphine)palladium(II) dichloride (5.4 mg, 0.008 mmol, 0.06 equiv) and copper iodide (4.9 mg, 0.026 mmol, 0.2 equiv) were added subsequently. After column chromatography on silica gel (pentane/CH_2Cl_2 = 12:1) the desired compound **9b** was obtained as a yellow oil (70.4 mg, 72%).

Analytical data of compound **9b**:

R_f: 0.17 (Hex/CH_2Cl_2 = 12:1).

$[\alpha]_D^{20}$ = +7.1° (c = 0.65, $CHCl_3$).

^{1}H-NMR (300 MHz, $CDCl_3$): δ = 1.01–1.12 (m, 63H, TIPS-H), 2.33 (s, 3H, 3 × 7'-H), 3.87–3.92 (dd, *J* = 11.4, 4.8 Hz, 1H, 6-H), 3.99–4.07 (m, 2H, 3-H, 6-H'), 4.07–4.11, 1H, 5-H), 4.30–4.37, 1H, 4-H), 5.30 (dd, *J* = 5.4, 1.6 Hz, 1H, 2-H), 7.10 (d, *J* = 8.0 Hz, 2H, 5'-H), 7.36 (d, *J* = 8.0 Hz, 2H, 5'-H).

^{13}C-NMR (125 MHz, $CDCl_3$): δ = 12.0, 12.3, 12.5 (CH-TIPS), 18.0, 18.1 (CH_3-TIPS), 21.5 (C-7'), 61.7 (C-6), 66.0 (C-3), 69.5 (C-5), 81.4 (C-4), 84.4 (C-1'), 87.3 (C-2'), 106.7 (C-2), 119.3 (C-3'), 128.9 (C-5'), 131.7 (C-4'), 136.3 (C-6'), 138.7 (C-1).

IR (film): ν (cm^{-1}) = 2726, 2624, 2351, 2203, 1555, 1538, 1510, 1368, 1342, 1315, 1248.

MS (ESI): *m/z* (%) = 751.50 (100) $[M+Na]^+$.

$C_{42}H_{76}O_4Si_3$ (729.31) calc.: 751.49491

found: 751.49474, $[M+Na]^+$ (ESI-HRMS).

1-(4-Methoxyphenylethynyl)-3,4,6-tri-*O*-isopropylsilylglucal (9c)

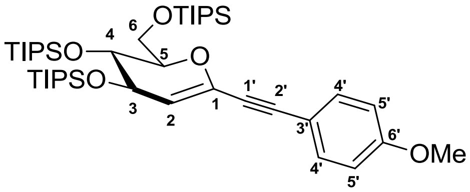

The reaction was performed according to general procedure **a**. 1-Iodoglucal **7** (100 mg, 0.135 mmol, 1.0 equiv) and 1-ethynyl-4-methoxybenzene (32 mg, 0.241 mmol, 1.8 equiv) were dissolved in NEt_3 (4 mL). Bis(triphenylphosphine)palladium(II) dichloride (6.9 mg, 0.01 mmol, 0.07 equiv) and copper iodide (7.7 mg, 0.04 mmol, 0.3 equiv) were added subsequently. After column chromatography on silica gel (pentane/CH_2Cl_2 = 12:1) the desired compound **9c** was obtained as a yellow oil (86.4 mg, 86%).

Analytical data of compound **9c**:

R_f: 0.26 (Hex/CH_2Cl_2 = 8:1).

$[\alpha]_D^{20}$ = –17.7° (c = 0.6, $CHCl_3$).

^{1}H-NMR (300 MHz, $CDCl_3$): δ = 0.80–1.17 (m, 63H, TIPS-H), 3.79 (s, 3H, OMe), 3.87–3.92 (dd, *J* = 11.1, 5.3 Hz, 1H, 6-H), 3.99–4.07 (m, 2H, 3-H, 6-H'), 4.07–4.10 (m, 1H, 5-H), 4.30–4.37 (m, 1H, 4-H), 5.28 (dd, *J* = 5.3, 1.6 Hz, 1H, 2-H), 6.81 (dt, *J* = 9.4, 2.8 Hz, 2H, 5'-H_{Ar}) 7.40 (dt, *J* = 9.1, 2.9 Hz, 2H, 4'-H_{Ar}).

^{13}C-NMR (125 MHz, $CDCl_3$): δ = 12.0, 12.3, 12.4 (CH-TIPS), 18.0, 18.1 (CH_3-TIPS), 55.26 (OMe), 61.7 (C-6), 66.0 (C-3), 69.5 (C-5), 81.4 (C-4), 83.8 (C-1'), 87.1 (C-2'), 106.4 (C-2), 113.8 (C-5'), 114.5 (C-3'), 133.3 (C-4'), 136.3 (C-1), 159.8 (C-6').

IR (film): ν (cm^{-1}) = 2759, 2726, 2560, 2200, 2044, 1922.

MS (ESI): *m*/*z* (%) = 767.6 (100) $[M+Na]^+$.

$C_{42}H_{76}O_5Si_3$ (745.31) calc.: 767.48982

found: 767.48949, $[M+Na]^+$ (ESI-HRMS).

1-(4-Fluorophenylethynyl)-3,4,6-tri-*O*-isopropylsilylglucal (9d)

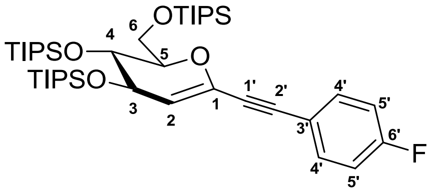

The reaction was performed according to general procedure **a**. 1-Iodoglucal **7** (115 mg, 0.155 mmol, 1.0 equiv) and 1-ethynyl-4-fluorobenzene (44 mg, 0.366 mmol, 2.4 equiv) were dissolved in NEt_3 (4 mL). Bis(triphenylphosphine)palladium(II) dichloride (8.5 mg, 0.012 mmol, 0.08 equiv) and copper iodide (5.6 mg, 0.029 mmol, 0.18 equiv) were added subsequently. After column chromatography on silica gel (pentane/CH_2Cl_2 = 12:1) the desired compound **9d** was obtained as a yellow oil (93 mg, 82%).

Analytical data of compound **9d**:

R_f: 0.21 (hexane/CH_2Cl_2 = 6:1).

$[\alpha]_D^{20}$ = –16.9° (c = 0.49, $CHCl_3$).

^{1}H-NMR (300 MHz, $CDCl_3$): δ = 0.96–1.12 (m, 63H, TIPS-H), 3.88 (dd, J = 11.2, 5.5 Hz, 1H, 6-H), 4.32–4.36 (m, 3H, 3-H, 5-H, 6-H'), 4.32–4.36 (m, 1H, 4-H), 5.31 (dd, J = 5.0, 1.7 Hz, 1H, 2-H), 6.99 (dt, J = 8.5, 2.0 Hz, 2H, 5-H'$_{Ar}$), 7.44 (dt, J = 8.5, 2.0 Hz, 2H, 4'-H$_{Ar}$).

^{13}C-NMR (125 MHz, $CDCl_3$): δ = 12.0, 12.3, 12.4 (CH-TIPS), 18.0, 18.1 (CH_3- TIPS), 61.6 (C-6), 65.9 (C-3), 69.4 (C-5), 81.5 (C-4), 84.8 (C-1'), 86.0 (C-2'), 107.1 (C-2), 115.4, 115.6 (C-5', J_{C-F} = 22 Hz), 118.5 (C-3', J_{C-F} = 5.0 Hz), 133.7 (C-4', J_{C-F} = 8.5 Hz), 136.0 (C-1), 161.6, 163.6, (C-6', J_{C-F} = 257 Hz).

IR (Film): ν (cm^{-1}) = 2757, 2725, 2623, 2351, 2208, 1887, 1555, 1538.

MS (ESI): *m/z* (%) = 755.47 (100) $[M+Na]^+$.

$C_{41}H_{73}FO_4Si_3$ (734.28) calc.: 755.46984

found: 755.46928, $[M+Na]^+$ (ESI-HRMS).

1-(3-Methylphenylethynyl)-3,4,6-tri-*O*-isopropylsilylglucal (9e)

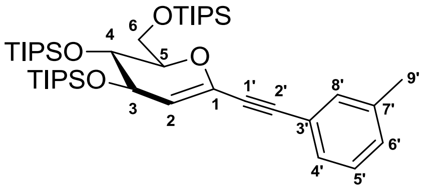

The reaction was performed according to general procedure **a**. 1-Iodoglucal **7** (500 mg, 0.677 mmol, 1.0 equiv) and 1-ethynyl-3-methylbenzene (86.8 mg, 96.5 µL, 0.745 mmol, 1.1 equiv) were dissolved in NEt_3 (20 mL). Bis(triphenylphosphine)palladium(II) dichloride (23.7 mg, 0.034 mmol, 0.05 equiv) and copper iodide (12.9 mg, 0.068 mmol, 0.1 equiv) were added subsequently. After column chromatography on silica gel (pentane/CH_2Cl_2 = 12:1) the desired compound **9e** was obtained as a yellow oil (442.4 mg, 90%).

Analytical data of compound **9e**:

R_f: 0.16 (hexane/CH_2Cl_2 = 12:1).

$[\alpha]_D^{20}$ = –13.9° (c = 0.38, $CHCl_3$).

^{1}H-NMR (300 MHz, $CDCl_3$): δ = 0.93–1.12 (m, 63H, TIPS-H), 2.30 (s, 3H, 9'-H), 3.90 (dd, *J* = 11.4, 5.0 Hz, 1H, 6-H), 4.00–4.08 (m, 2H, 3-H, 6-H'), 4.08–4.11 (m, 1H, 5-H), 4.31–4.37 (m, 1H, 4-H), 5.31 (dd, *J* = 5.5, 1.7 Hz, 1H, 2-H), 7.09–7.31 (m, 4H, 4'-H, 5'-H, 6'-H, 8'-H).

^{13}C-NMR (125 MHz, $CDCl_3$): δ = 12.0, 12.3, 12.5 (CH-TIPS), 18.0, 18.1 (CH_3-TIPS), 21.1 (C-9'), 61.7 (C-6), 65.9 (C-3), 69.5 (C-5), 81.4 (C-4), 84.7 (C-1'), 87.3 (C-2'), 106.9 (C-2), 122.2 (C-3'), 128.1 (C-5'), 128.8 (C-6'), 129.4 (C-4'), 132.3 (C-8'), 136.2 (C-7'), 137.8 (C-1).

IR (film): ν (cm^{-1}) = 2758, 2726, 2622, 2562, 2352, 2218, 2192, 1940, 1538, 1515.

MS (ESI): *m/z* (%) = 729.51 (100) $[M+Na]^+$.

$C_{42}H_{76}O_4Si_3$ (729.31) calc.: 751.49491

found.: 751.49480, $[M+Na]^+$ (ESI-HRMS).

1-(Trimethylsilylethynyl)-3,4,6-tri-*O*-isopropylsilylglucal (9f)

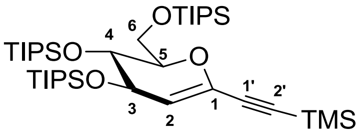

The reaction was performed according to general procedure **a**. 1-Iodoglucal **7** (59 mg, 0.079 mmol, 1.0 equiv) and ethynyltrimethylsilane (16 mg, 0.158 mmol, 2.0 equiv) were dissolved in NEt_3 (2 mL). Bis(triphenylphosphine)palladium(II) dichloride (2.8 mg, 0.004 mmol, 0.05 equiv) and copper iodide (1.5 mg, 0.008 mmol, 0.1 equiv) were added subsequently. After column chromatography on silica gel (pentane/CH_2Cl_2 = 8:1) the desired compound **9f** was obtained as a yellow oil (44.2 mg, 79%).

Analytical data of compound **9f**:

$\mathbf{R_f}$: 0.25 (hexane/CH_2Cl_2 = 8:1).

$[\alpha]_D^{20}$ = –119.0° (c = 0.67, $CHCl_3$).

^{1}H-NMR (300 MHz, $CDCl_3$): δ = 0.12–0.21 (m, 9H, TMS-H), 0.98–1.09 (m, 63H, TIPS-H), 3.81–3.86 (dd, *J* = 11.2, 4.9 Hz, 1H, 6-H), 3.93–4.02 (m, 2H, 3-H, 6-H'), 4.03–4.06 (m, 1H, 5-H), 4.25–4.33 (m, 1H, 4-H), 5.24 (dd, *J* = 5.4, 1.6 Hz, 1H, 2-H).

^{13}C-NMR (125 MHz, $CDCl_3$): δ = –0.29 (CH_3-TMS), 12.0, 12.2, 12.4 (CH-TIPS), 18.9, 18.1 (CH_3-TIPS), 61.5 (C-6), 65.8 (C-3), 69.4 (C-5), 81.3 (C-4), 92.6 (C-1'), 100.0 (C-2'), 107.4 (C-2), 135.8 (C-1).

IR (film): ν (cm^{-1}) = 3065, 2757, 2725, 2623, 2562, 2485, 2243, 1947, 1898, 1805, 1494.

$C_{38}H_{78}O_4Si_4$ (710.50) calc.: 711.50554
found: 711.50478, $[M+H]^+$ (ESI-HRMS).

1-Hexyn-1-yl-3,4,6-tri-*O*-isopropylsilylglucal (9g)

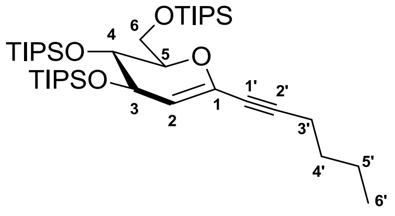

The reaction was performed according to general procedure **a**. 1-Iodoglucal **7** (100 mg, 0.135 mmol, 1.0 equiv) and 1-hexyne (22.2 mg, 31 µL, 0.270 mmol, 2.0 equiv) were dissolved in NEt_3 (4 mL). Bis(triphenylphosphine)palladium(II) dichloride (6.9 mg, 0.01 mmol, 0.07 equiv) and copper iodide (8.7 mg, 0.046 mmol, 0.3 equiv) were added subsequently. After column chromatography on silica gel (pentane/CH_2Cl_2 = 12:1) the desired compound **9g** was obtained as a yellow oil (77.9 mg, 83%).

Analytical data of compound **9g**:

$\mathbf{R_f}$: 0.18 (hexane/CH_2Cl_2 = 12:1).

$[\alpha]_D^{20}$ = –9.3° (c = 4.54, $CHCl_3$).

^{1}**H-NMR** (300 MHz, $CDCl_3$): δ = 0.89 (dt, *J* = 7.2, 1.6 Hz, 3H, 6'-H), 0.89–1.12 (m, 63H, H-TIPS), 1.33–1.54 (m, 4H, 4'-H, 5'-H), 2.21–2.31 (m, 2H, 3'-H), 3.86 (dd, *J* = 11.1, 5.0 Hz, 1H, 6-H), 3.92–4.01 (m, 2H, 3-H, 6-H'), 4.03–4.07 (m, 1H, 5-H), 4.24–4.30 (m, 1H, 4-H), 5.10 (dd, *J* = 5.4, 1.5 Hz, 1H, 2-H).

^{13}**C-NMR** (125 MHz, $CDCl_3$): δ = 12.0, 12.3, 12.5 (CH-TIPS), 13.5 (C-6'), 18.0, 18.1, 18.2 (CH_3-TIPS), 18.9 (C-3'), 21.9 (C-4'), 30.4 (C-5'), 61.7 (C-6), 65.2 (C-1'), 66.0 (C-3), 69.5 (C-5), 81.2 (C-4), 88.6 (C-2'), 105.2 (C-2), 136.3 (C-1).

IR (film): ν (cm^{-1}) = 2757, 2725, 2623, 2562, 1929, 1728, 1549.

MS (ESI): *m/z* (%) = 695.53 (100) $[M+H]^+$.

$\mathbf{C_{39}H_{78}O_4Si_3}$ (695.29) calc.: 695.52794

found: 695.52794, $[M+H]^+$ (ESI-HRMS).

1-Pentyn-1-yl-3,4,6-tri-*O*-isopropylsilylglucal (9h)

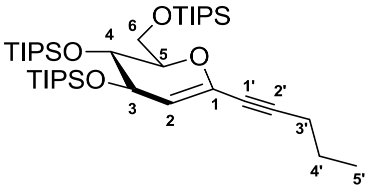

The reaction was performed according to general procedure **a**. 1-Iodoglucal **7** (502 mg, 0.677 mmol, 1.0 equiv) and 1-pentyne (50.7 mg, 74 µL, 0.745 mmol, 1.1 equiv) were dissolved in NEt_3 (20 mL). Bis(triphenylphosphine)palladium(II) dichloride (23.7 mg, 0.034 mmol, 0.05 equiv) and copper iodide (12.8 mg, 0.067 mmol, 0.1 equiv) were added subsequently. After column chromatography on silica gel (pentane/CH_2Cl_2 = 12:1) the desired compound **9h** was obtained as a yellow oil (442 mg, 96%).

Analytical data of compound **9h**:

R_f: 0.20 (hexane/CH_2Cl_2 = 10:1).
$[\alpha]_D^{20}$ = –15.6° (c = 0.5, $CHCl_3$).
^{1}H-NMR (300 MHz, $CDCl_3$): δ = 0.96 (t, *J* = 7.1 Hz, 3H, 5'-H), 0.99–1.12 (m, 63H, TIPS-H), 1.55 (hex, *J* = 7.1, 7.5, 13.8 Hz, 2H, 4'-H), 2.26 (t, *J* = 7.1 Hz, 2H, 3'-H), 3.86 (dd, *J* = 10.8, 5.1 Hz, 1H, 6-H), 3.93–4.01 (m, 2H, 3-H, 6-H'), 4.05–4.07 (m, 1H, 5-H), 4.23–4.30 (m, 1H, 4-H), 4.06 (dd, *J* = 3.9, 2.0 Hz, 1H, 2-H).
^{13}C-NMR (125 MHz, $CDCl_3$): δ = 12.1, 12.4, 12.6 (CH-TIPS), 13.6 (5'-C), 18.1, 18.2, 18.3 (CH_3-TIPS), 21.2 (3'-C), 21.9 (4'-C), 61.7 (6-C), 66.1 (3-C), 69.5 (5-C), 76.6 (1'-C), 81.3 (4-C), 88.5 (2'-C), 105.3 (2-C), 136.3 (1-C).
IR (film): ν (cm^{-1}) = 2757, 2623, 2561, 2383, 1927, 1730, 1548.
MS (ESI): *m/z* (%) = 681.5 (100) $[M+H]^+$.
$C_{38}H_{76}O_4Si_3$ (681.26) calc.: 681.51262
found: 681.51262, $[M+H]^+$ (ESI-HRMS).

1-Phenylethynylglucal (9aa)

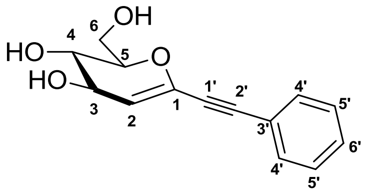

The reaction was performed according to general procedure **ba**. Compound **9a** (562 mg, 0.786 mmol, 1.0 equiv) was dissolved in THF (20 mL). A solution of tetrabutylammonium fluoride trihydrate (1.12 g, 3.54 mmol, 4.5 equiv) in THF (6 mL) was added dropwise. After column chromatography on silica gel (DCM/MeOH = 25:1) the desired compound **9aa** **(**151.0 mg, 78%) was obtained as a white solid.

Analytical data of compound **9aa**:

R_f: 0.17 (DCM/MeOH = 25:1).

$[\alpha]_D^{20}$ = +37.6° (c = 0.17, MeOH).

^{1}H-NMR (300 MHz, CD_3OD): δ = 3.58–3.64 (m, 1H, 4-H), 3.78–3.96 (m, 3H, 5-H, 6-H, 6-H'), 4.19 (dd, *J* = 7.2, 2.8 Hz, 1H, 3-H), 4.88 (s, 3H, OH), 5.20 (d, *J* = 2.8 Hz, 1H, 2-H), 7.32–7.37 (m, 3H, 5'-H, 6'-H), 7.39–7.45 (m, 2H, 4'-H).

^{13}C-NMR (125 MHz, CD_3OD): δ =62.0 (C-6), 70.2 (C-4), 71.0 (C-3), 81.3 (C-5), 84.5 (C-1'), 88.9 (C-2'), 111.4 (C-2), 123.3 (C-3'), 129.6 (C-5'), 130.0 (C-6'), 132.5 (C-4'), 138.7 (C-1).

IR (KBr): ν (cm^{-1}) = 2209, 1973, 1952, 1881, 1804, 1755, 1572.

MS (ESI): *m*/*z* (%) = 269.08 (100) $[M+Na]^+$.

$C_{14}H_{14}O_4$ (246.25) calc.: 269.07843

found: 269.07857, $[M+Na]^+$ (ESI-HRMS).

1-(Phenylethynyl)-3,4,6-tri-*O*-benzylglucal (9ab)

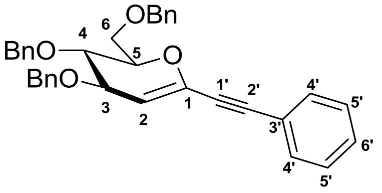

The reaction was performed according to general procedure **bb**. Compound **9aa** (100 mg, 0.407 mmol, 1.0 equiv) was dissolved in DMF (2.5 mL). Sodium hydride (60% in mineral oil) (53.6 mg, 1.34 mmol, 3.3 equiv) was added at 0 °C followed by dropwise addition of benzyl bromide (229.2 mg, 159 µL, 1.34 mmol, 3.3 equiv). After column chromatography on silica gel (hexane/EtOAc = 3:1) the desired compound **9ab** (207.0 mg, 99%) was obtained as a white solid.

Analytical data of compound **9ab**:

$\mathbf{R_f}$: 0.27 (hexane/EtOAc = 3:1).

$[\alpha]_D^{20}$ = –8.6° (c = 0.50, $CHCl_3$).

^{1}H-NMR (300 MHz, $CDCl_3$): δ = 3.76–3.87 (m, 2H, CH_2-Bn, 4-H), 3.91–3.96 (m, 1H, 5-H), 4.12–4.17 (m, 1H, 4-H), 4.27 (dd, *J* = 2.9, 5.6 Hz, 1H, 3-H), 4.52–4.70 (m, 6H, 6-H, 6-H', CH_2-Bn), 4.82 (d, *J* = 11.2 Hz, 1H, CH_2-Bn), 5.42 (d, *J* = 3.1 Hz, 1H, 2-H), 7.21–7.36 (m, 18H, 5'-H, 6'-H, H_{Bn}), 7.43–7.48 (m, 2H, 4'-H).

^{13}C-NMR (125 MHz, $CDCl_3$): δ = 68.2 (CH_2Bn), 70.6 (C-6), 73.5 (C-5), 73.7 (2 × CH_2Bn), 76.2 (C-4), 77.7 (C-4), 83.6 (C-1'), 88.6 (C-2'), 106.9 (C-2), 122.0 (C-3'), 127.6, 127.7, 127.8, 127.9, 128.3, 128.4, 128.7, 128.8, 129.0 (C_{Bn}), 131.7 (C-4'), 137.7, 138.0, 138.1 ($C_{q,Bn}$), 138.2 (C-1).

IR (KBr): ν (cm^{-1}) = 3442, 2726, 2336, 2022, 1955, 1881, 1816, 1712, 1671, 1570, 1398, 1181.

MS (ESI): *m/z* (%) = 539.3 (100) $[M+Na]^+$.

$\mathbf{C_{35}H_{32}O_4}$ (516.62)	calc.: 539.2193
	found: 539.2188, $[M+Na]^+$ (ESI-HRMS).

1-(3-Methylphenylethynyl)-3,4,6-tri-*O*-benzylglucal (9ea)

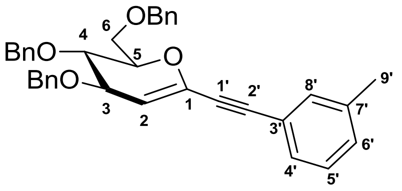

The reaction was performed according to general procedure **bb**. Compound **9eb** (89.6 mg, 0.345 mmol, 1.0 equiv) was dissolved in DMF (4 mL). Sodium hydride (60% in mineral oil) (55.1 mg, 1.38 mmol, 4.0 equiv) was added at 0 °C followed by dropwise addition of benzyl bromide (235.7 mg, 164 µL, 1.38 mmol, 4.0 equiv). After column chromatography on silica gel (hexane/EtOAc = 3:1) the desired compound **9ea** (175.0 mg, 96%) was obtained as a white solid.

Analytical data of compound **9ea**:

R_f: 0.42 (hexane/EtOAc = 3:1).

$[\alpha]_D^{20}$ = –6.3° (c = 0.24, $CHCl_3$).

^{1}H-NMR (300 MHz, $CDCl_3$): δ = 2.31 (s, 3H, 9'-H), 3.78–3.96 (m, 3H, 6-H, 6-H', 4-H), 4.08–4.16 (m, 1H, 5-H), 4.28 (dd, *J* = 3.1, 6.1 Hz, 1H, 3-H), 4.49–4.68 (m, 5H, CH_2-Bn), 4.82 (d, *J* = 11.4 Hz, 1H, CH_2-Bn), 5.41 (d, *J* = 3.5 Hz, 1H, 2-H), 7.12–7.37 (m, 19H, 4'-H, 5'-H, 6'-H, 8'-H, H_{Bn}).

^{13}C-NMR (125 MHz, $CDCl_3$): δ = 21.2 (C-9'), 68.2 (C-6), 70.5 (CH_2-Bn), 73.5 (CH_2-Bn), 73.7 (CH_2-Bn), 73.8 (C-4), 76.2 (C-3), 77.7 (C-5), 83.2 (C-1'), 88.8 (C-2'), 106.7 (C-2), 121.7 (C-3'), 127.5, 127.6, 127.7, 127.8, 128.2, 128.3, 128.7 (C-4', C-5', C_{Bn}), 129.6 (C-4'), 132.2 (C-8'), 137.9, 138.0, 138.2 (C_q-Bn, C-1).

IR (KBr): ν (cm^{-1}) = 3107, 2777, 2337, 2211, 1949, 1868, 1810, 1718, 1543.

MS (ESI): *m/z* (%) = 553.2 (100) $[M+Na]^+$.

$C_{36}H_{34}O_4$ (530.65) calc.: 553.2349

found: 553.2338, $[M+Na]^+$ (ESI-HRMS).

1-(3-Methylphenylethynyl)glucal (9eb)

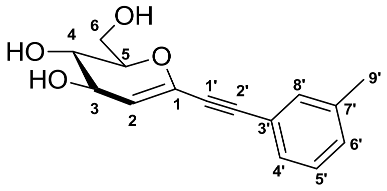

The reaction was performed according to general procedure **ba**. Compound **9e** (303 mg, 0.416 mmol, 1.0 equiv) was dissolved in THF (15 mL). A solution of tetrabutylammonium fluoride trihydrate (594 mg, 1.87 mmol, 4.5 equiv) in THF (4 mL) was added dropwise. After column chromatography on silica gel (DCM/MeOH = 15:1) the desired compound **9eb** (97.0 mg, 90%) was obtained as a white solid.

Analytical data of compound **9eb**:

$\mathbf{R_f}$: 0.05 (MeOH/DCM = 15:1).

$[\alpha]_D^{20}$ = +40.9° (c = 0.22, MeOH).

^{1}H-NMR (300 MHz, CD_3OD): δ = 2.31 (s, 3H, 9'-H), 3.57–3.65 (m, 1H, 3-H), 3.78–3.96 (m, 3H, 5-H, 6'-H), 4.19 (dd, *J* = 3.3, 7.0 Hz, 1H, 4-H), 5.17 (d, *J* = 2.7 Hz, 1H, 2-H), 7.16–7.25 (m, 4H, 4'-H, 5'-H, 6'-H, 8'-H).

^{13}C-NMR (125 MHz, CD_3OD): δ =21.2 (C-9'), 62.0 (C-6), 70.2 (C-3), 71.0 (C-4), 81.2 (C-5), 84.1 (C-1'), 89.1 (C-2'), 111.2 (C-2), 123.0 (C-3'), 129.3 (C-5'), 129.5 (C-6'), 130.7 (C-4'), 132.8 (C-8'), 138.6 (C-7'), 139.4 (C-1).

IR (KBr): ν (cm^{-1}) = 2627, 1958, 188, 1818, 1789, 1754,1699.

MS (ESI): *m*/*z* (%) = 283.1 (100) $[M+Na]^+$.

$\mathbf{C_{15}H_{16}O_4}$ (260.29) calc.: 283.0941

found: 283.0948, $[M+Na]^+$ (ESI-HRMS).

2-(Phenylethynyl)-3,4,6-tri-*O*-acetylgalactal (11a)

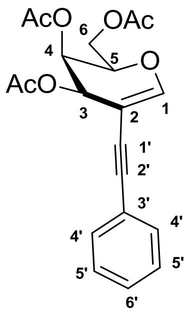

The reaction was performed according to general procedure **a** at 70 °C. The 2-Bromogalactal **10** (70.2 mg, 0.200 mmol, 1.0 equiv) and phenylacetylene (27 µL, 0.240 mmol, 1.2 equiv) were dissolved in NEt_3 (2 mL). Bis(triphenylphosphine)palladium(II) dichloride (7.0 mg, 0.01 mmol, 0.05 equiv) and copper iodide (3.8 mg, 0.02 mmol, 0.1 equiv) were added subsequently. After column chromatography on silica gel (pentane/EtOAc = 9:1 → pentane/EtOAc = 3:1) the desired compound **11a** was obtained as a yellowish solid (49.0 mg, 66%).

Analytical data of compound **11a**:

$\mathbf{R_f}$: 0.38 (hexane/EtOAc = 3:1).

$[\alpha]_D^{20}$ = +54.0° (c = 0.10, $CHCl_3$).

^{1}**H-NMR** (300 MHz, $CDCl_3$): δ = 2.03–2.15 (m, 9H,3 × CH_3^{Ac}), 4.21–4.41 (m, 3H, 4-H, 6-H), 5.50 (dd, *J* = 1.6 Hz, 4.5 Hz, 1H, 3-H), 6.94 (s, 1H, 1-H), 7.26–7.37 (m, 5H, H^{Ph}).

^{13}**C-NMR** (125 MHz, $CDCl_3$): δ = 20.7 (CH_3^{Ac}), 20.7 (CH_3^{Ac}), 20.7 (CH_3^{Ac}), 61.7 (C-6), 63.2 (C-3), 64.4 (C-4), 73.5 (C-5), 83.3 (C-1'), 90.3 (C-2'), 97.0 (C-2), 123.1 (C- Ph), 127.9 (C-Ph), 128.2 (C-Ph), 130.1 (C-Ph), 150.4 (C-1), 169.9 ($C{=}O^{Ac}$), 170.0 ($C{=}O^{Ac}$), 170.3 ($C{=}O^{Ac}$).

IR (ATR): ν (cm^{-1}) = 3082, 2965, 2934, 2340, 1593, 1488, 1407, 1078.

MS (ESI): *m*/*z* (%) = 395.2 (100) $[M+Na]^+$.

$\mathbf{C_{20}H_{20}O_7}$ (372.37) calc.: 395.1101

found: 395.1103, $[M+Na]^+$ (ESI-HRMS).

2-(Trimethylsilylethynyl)-3,4,6-tri-*O*-acetylgalactal (11b)

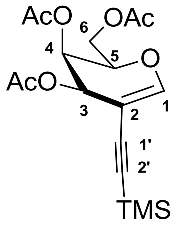

The reaction was performed according to general procedure **a** at 70 °C. 2-Bromogalacal **10** (351 mg, 1.00 mmol, 1.0 equiv) and trimethylsilylacetylene (160 mg, 1.63 mmol, 1.6 equiv) were dissolved in a mixture of NEt_3:THF (4:1, 5 mL). Bis(triphenylphosphine)palladium(II) dichloride (35.0 mg, 0.05 mmol, 0.05 equiv) and copper iodide (19.0 mg, 0.10 mmol, 0.1 equiv) were added subsequently. After column chromatography on silica gel (pentane/EtOAc = 9:1 → pentane/EtOAc = 3:1) the desired compound **11b** was obtained as a brownish solid (160 mg, 45%).

Analytical data of compound **11b**:

R_f: 0.49 (hexane/EtOAc = 3:1).

$[\alpha]_D^{20}$ = +73.5° (c = 0.23, $CHCl_3$).

^{1}H-NMR (300 MHz, $CDCl_3$): δ = 0.13–0.23 (m, 9H, 3 × CH_3^{TMS}), 2.03 (s, 3H, CH_3^{Ac}), 2.07 (s, 3H, CH_3^{Ac}), 2.12 (s, 3H, CH_3^{Ac}), 4.18–4.34 (m, 3H, 4-H, 6-H), 4.83 (dd, *J* = 1.5 Hz, 4.8 Hz, 1H, 3-H), 5.64–5.68 (m, 1H, 5-H), 6.87 (d, *J* = 1.5 Hz, 1 H, 1-H).

^{13}C-NMR (125 MHz, $CDCl_3$): δ = 0.0 (CH_3^{TMS}) 20.6 (CH_3^{Ac}), 20.7 (CH_3^{Ac}), 20.7 (CH_3^{Ac}), 61.6 (C-6), 63.0 (C-3), 64.1 (C-4), 73.6 (C-5), 95.5 (C-1'), 97.1 (C-2'), 98.9 (C-2), 151.3 (C-1), 169.9 (C=O^{Ac}), 170.2 (C=O^{Ac}).

IR (ATR): ν (cm^{-1}) = 2963, 2899, 2144, 1434, 1410, 1314.

MS (ESI): *m/z* (%) = 391.1 (100) $[M+Na]^+$.

$C_{17}H_{24}O_7Si$ (368.45) calc.: 391.1184

found: 391.1177, $[M+Na]^+$ (ESI-HRMS).

1-Phenylethynyl-3,4,6-tri-*O*-benzyl-2-chloroglucal (13)

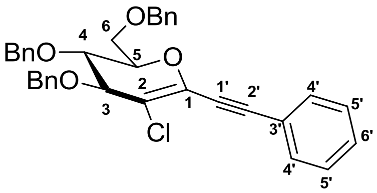

The reaction was performed according to general procedure **a** under reflux. 2-Chloro-1-iodoglucal **12** (48.8 mg, 0.087 mmol, 1.0 equiv) and phenylacetylene (20.3 mg, 21.5 µL, 0.199 mmol, 2.3 equiv) were dissolved in NEt_3 (5 mL). Bis(triphenylphosphine)palladium(II) dichloride (6.1 mg, 0.008 mmol, 0.1 equiv) and copper iodide (3.3 mg, 0.017 mmol, 0.2 equiv) were added subsequently. After column chromatography on silica gel (pentane/EtOAc = 10:1) the desired compound **13** (47.8 mg, >99%) was obtained as a brownish oil.

Analytical data of compound **13**:

$\mathbf{R_f}$: 0.19 (hexane/EtOAc = 10:1).

$[\alpha]_D^{20}$ = +10.9° (c = 0.23, $CHCl_3$).

^{1}H-NMR (300 MHz, $CDCl_3$): δ = 3.74–3.85 (m, 2H, 6-H, 5-H), 4.01–4.04 (m, 1H, 4-H), 4.23 (d, *J* = 4.5 Hz, 1H, 3-H), 4.28–4.33 (m, 1H, 6-H'), 4.50–4.75 (m, 6H, CH_2-Bn), 7.20–7.38 (m, 18H, 5'-H, 6'-H, H_{Bn}), 7.50–7.53 (m, 2H, 4'-H).

^{13}C-NMR (125 MHz, $CDCl_3$): δ = 67.6 (C-6), 72.4 (CH_2-Bn), 72.9 (CH_2-Bn), 73.4 (CH_2-Bn), 73.7 (C-4), 77.3 (C-3), 78.0 (C-5), 81.2 (C-1'), 95.1 (C-2'), 115.8 (C-2), 121.6 (C-3'), 127.6, 127.7, 127.8, 127.9, 128.2, 128.3, 128.4 (C_{Bn}, C-5'), 129.1 (C-6'), 131.8 (C-4') 136.2 (C-1), 137.4, 137.6, 137.7 ($C_{q,Bn}$).

IR (film): ν (cm^{-1}) = 2510, 2335, 1954, 1881, 1808.

MS (ESI): *m*/*z* (%) = 573.2 (100) $[M+Na]^+$.

$C_{35}H_{31}ClO_4$ (551.07) calc.: 573.1803

found: 573.1807, $[M+Na]^+$ (ESI-HRMS).

1-(2-(3-Methylphenyl)ethyl)-3,4,6-tri-*O*-isopropylsilylglucal (14a)

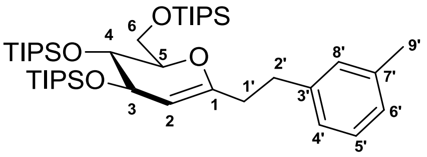

The reaction was performed according to general procedure **ca**. A solution of compound **9e** (128.5 mg, 0.176 mmol, 1.0 equiv) in a mixture of MeOH and THF (1:1, 10 mL) was added to a slurry of Raney nickel. The reaction mixture was stirred for 3 h at room temperature. After column chromatography on silica gel (pentane/ CH_2Cl_2 = 12:1) the desired compound **14a** (113.0 mg, 88%) was obtained as a clear oil.

Analytical data of compound **14a**:

$\mathbf{R_f}$: 0.32 (hexane/CH_2Cl_2 = 12:1).

$[\alpha]_D^{20}$ = –13.2° (c = 0.22, $CHCl_3$).

^{1}H-NMR (300 MHz, $CDCl_3$): δ = 0.84–1.11 (m, 61H, TIPS-H), 2.28–2.33 (m, 5H, 1'-H, 9'-H), 2.71–2.83 (m, 2H, 2'-H), 3.81 (dd, *J* = 5.1, 11.7 Hz, 1H, 5-H), 3.95–4.03 (m, 3H, 4-H, 6-H, 6-H'), 4.26–4.27 (m, 1H, 3-H), 4.60 (dd, *J* = 1.1, 5.0 Hz, 1H, 2-H), 6.95–7.01 (m, 3H, 4'-H 6'-H 8'-H), 7.11–7.16 (m, 1H, 5'-H).

^{13}C-NMR (125 MHz, $CDCl_3$): δ = 12.1, 12.4, 12.6, (TIPS-CH), 18.1, 18.2, 18.4, 18.6 (TIPS-CH_3), 21.4 (C-9'), 33.6 (C-2'), 36.5 (C-1'), 62.2 (C-6), 66.4 (C-5), 69.8 (C-3), 81.0 (C-4), 95.8 (C-2), 125.3 (C-4'), 126.3 (C-6'), 128.0 (C-5'), 129.0 (C-8'), 137.6 (C-7'), 141.9 (C-3'), 152.8 (C-1).

IR (film): ν (cm^{-1}) = 2723, 2352, 1729, 1610, 1555, 1367, 1344, 1313, 1246.

MS (ESI): *m/z* (%) = 755.5 (100) $[M+Na]^+$.

$\mathbf{C_{42}H_{80}O_4Si_3}$ (733.33) calc.: 755.5257

found: 755.5255, $[M+Na]^+$ (ESI-HRMS).

1-(2-(3-Methylphenyl)ethyl)-3,4,6-tri-*O*-benzylglucal (14b)

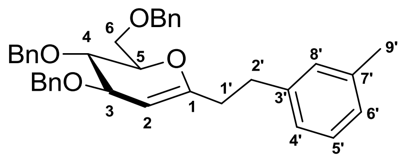

The reaction was performed according to general procedure **bb**. Compound **9eb** (17.6 mg, 0.066 mmol, 1.0 equiv) was dissolved in DMF (1 mL). Sodium hydride (60% in mineral oil) (10.6 mg, 0.266 mmol, 4.0 equiv) was added at 0 °C followed by the dropwise addition of benzyl bromide (45.5 mg, 32 µL, 0.266 mmol, 4.0 equiv). After column chromatography on silica gel (pentane/EtOAc = 3:1) the desired compound **14b** (17.2 mg, 49%) was obtained as a clear oil.

Analytical data of compound **14b**:

R_f: 0.50 (hexane/EtOAc = 3:1).

$[\alpha]_D^{20}$ = 0.0° (c = 0.22, $CHCl_3$).

^{1}H-NMR (300 MHz, $CDCl_3$): δ = 2.32 (s, 3H, 9'-H), 2.39 (t, *J* = 8.18 Hz, 2H, 2'-H), 2.72–2.82 (m, 2H, 1'-H), 3.76–3.88 (m, 3H), 4.09–4.19 (m, 2H), 4.41–4.70 (m, 6H, CH_2Bn), 4.83 (d, *J* = 11.9 Hz, 2H, 2-H), 7.01 (d, *J* = 7.5 Hz, 2H), 7.14–7.19 (m, 1H), 7.24–7.38 (m, 15H, Bn-H).

^{13}C-NMR (125 MHz, $CDCl_3$): δ = 21.4 (C-9'), 33.1 (C-2'), 35.6 (C-1'), 68.6 (C-6*), 69.9 (C-5*), 73.2, 73.4, 74.1 (3 × CH_2Bn*), 75.8 (C-3*), 76.7 (C-4*) 95.4 (C-2), 125.3 (C-4'*), 126.5 (C-6'*), 127.4, 127.5, 127.8, 128.0, 128.2 (C-Bn), 129.1 (C-5'*), 137.6, 138.1, 138.4 (C-Bn, C-7'*), 141.2 (C-3'*), 155.3 (C-1).

IR (film): ν (cm^{-1}) = 2731, 1949, 1873, 1808, 1548.

MS (ESI): *m/z* (%) = 537.3 (100) $[M+Na]^+$.

$C_{36}H_{38}O_4$ (534.08) calc.: 557.2662

found: 557.2664, $[M+Na]^+$ (ESI-HRMS).

1-Pentyl-3,4,6-tri-*O*-isopropylsilylglucal (14c)

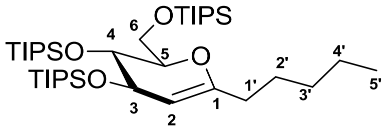

The reaction was performed according to general procedure **ca**. A solution of compound **9h** (111.0 mg, 0.162 mmol, 1.0 equiv) in a mixture of MeOH and THF (1:1, 10 mL) was added to a slurry of Raney nickel. The reaction was stirred for 4 h at room temperature. After column chromatography on silica gel (pentane/CH_2Cl_2 = 12:1) the desired compound **14c** (69.0 mg, 63%) was obtained as a clear oil.

Analytical data of compound **14c**:

R_f: 0.43 (hexane/CH_2Cl_2 = 12:1).

$[\alpha]_D^{20}$ = –16.4° (c = 0.76, $CHCl_3$).

^{1}H-NMR (300 MHz, $CDCl_3$): δ = 0.83–0.87 (m, 5H), 0.94–1.13 (m, 61H), 1.24–1.29 (m, 6H), 1.41–1.54 (m, 3H), 2.00 (t, *J* = 7.7 Hz, 2H), 3.80 (dd, *J* = 4.8, 10.9 Hz, 1H, 6-H), 3.94–4.00 (m, 3H), 4.20–4.24 (m, 1H), 4.56 (dd, *J* = 1.7, 5.6 Hz, 1H, 2-H).

^{13}C-NMR (125 MHz, $CDCl_3$): δ = 12.1, 12.4, 12.6 (TIPS-CH), 14.0 (C-5'), 18.0, 18.1, 18.2 (TIPS-CH_3), 22.6 (C-4'), 26.7 (C-2'), 31.3 (C-3'), 34.4 (C-1'), 62.3 (C-6), 66.5 (C-5), 69.8 (C-3), 80.9 (C-4), 95.4 (C-2), 153.5 (C-1).

IR (film): ν (cm^{-1}) = 2725, 2563, 2351, 2243, 2126, 1915, 1806, 1548, 1538, 1514.

MS (ESI): *m*/*z* (%) = 685.6 (100) $[M+H]^+$, 707.5 (79) $[M+Na]^+$.

$C_{38}H_{80}O_4Si_3$ (684.30) calc.: 685.5437
found: 685.5438, $[M+H]^+$ (ESI-HRMS).

1-(2-(3-Methyphenyl)ethyl)glucal (14aa)

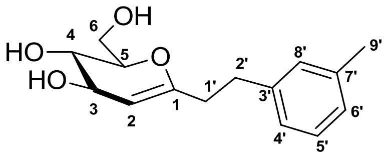

The reaction was performed according to general procedure **ba**. Compound **14a** (64.5 mg, 0.088 mmol, 1.0 equiv) was dissolved in THF (3.2 mL). A solution of tetrabutylammonium fluoridetrihydrate (125.6 mg, 0.396 mmol, 4.5 equiv) in THF (0.8 mL) was added dropwise. After column chromatography on silica gel (CH_2Cl_2/MeOH = 15:1) the desired compound **14aa** (22.2 mg, 96%) was obtained as a white solid.

Analytical data of compound **14aa**:

R_f: 0.09 (CH_2Cl_2/MeOH = 15:1).

$[\alpha]_D^{20}$ = +4.5° (c = 0.20, MeOH).

^{1}H-NMR (300 MHz, CD_3OD): δ =2.19–2.38 (m, 5H, 3 × 9'-H, 2 × 1'-H), 2.75 (t, *J* = 7.7 Hz, 2H, 2 × 2'-H), 3.48–3.54 (m, 1H, 5-H), 3.67–3.73 (m, 1H, 4-H), 3.76–3.92 (m, 2H, 2 × 6-H), 4.03–4.07 (m, 1H, 3-H), 4.44 (d, *J* = 2.3 Hz, 1H, 2-H), 6.93–7.01 (m, 3H, 4'-H, 6'-H, 8'-H), 7.08–7.16 (m, 1H, 5'-H).

^{13}C-NMR (125 MHz, CD_3OD): δ =21.4 (C-9'), 34.2 (C-2'), 36.4 (C-1'), 62.4 (C-6), 71.0 (C-5), 71.2 (C-3), 80.3 (C-4), 100.0 (C-2), 126.3 (C-4'), 127.4 (C-6'), 129.0 (C-5'), 130.0 (C-8'), 138.7 (C-7'), 142.6 (C-3'), 155.6 (C-1).

IR (KBr): ν (cm^{-1}) = 1936, 1590, 1556.

MS (ESI): *m*/*z* (%) = 287.1 (100) $[M+Na]^+$.

$C_{15}H_{20}O_4$ (264.31) calc.: 287.1254

found: 287.1256, $[M+Na]^+$ (ESI-HRMS).

3,4,6-Tri-*O*-acetyl-1,2-dideoxy-2-(*R*)-(2-phenylethyl)galactose (14d)

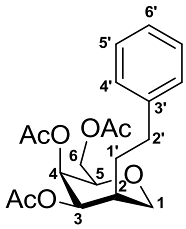

The reaction was performed according to general procedure **cb**. To a solution of compound **11a** (28.5 mg, 0.077 mmol, 1.0 equiv) in a mixture of MeOH:CH_2Cl_2:EtOAc (3:1:1, 5 mL) was added Perlman's catalyst (10 mg). The reaction was stirred overnight at room temperature. After column chromatography on silica gel (pentane/EtOAc = 9:1 → pentane/EtOAc = 3:1) the desired compound **14d** (28.0 mg, 97%) was obtained as a yellowish oil.

Analytical data of compound **14d**:

R_f: 0.34 (hexane/EtOAc = 3:1).

$[\alpha]_D^{20}$ = –5.5° (c = 0.20, $CHCl_3$).

^{1}H-NMR (300 MHz, $CDCl_3$): δ =1.88–1.94 (m, 1H, 2-H), 2.01–2.07 (m, 9H, 3 × CH_3^{Ac}), 2.45–2.60 (m, 2H, 1'-H, 2'-H), 2.65–2.80 (m, 2H, 1'-H, 2'-H), 3.49–3.61 (m, 1H, 1-H), 3.84–4.00 (m, 2H, 1-H, 5-H), 4.06–4.14 (m, 1H, 6-H), 4.26–4.32 (m, 1H, 6-H), 5.16–5.23 (m, 2H, 3-H, 4-H), 7.13–7.32 (m, 5H, H^{Ph}).

^{13}C-NMR (125 MHz, $CDCl_3$): δ =20.9 (CH_3^{Ac}), 20.9 (CH_3^{Ac}), 20.9 (CH_3^{Ac}), 27.5 (C-1'), 33.8 (C-2'), 37.5 (C-2), 61.8 (C-6), 66.9 (C-1), 67.8 (C-4), 70.0 (C-3), 74.4 (C-5), 125.9 (C-Ph), 128.2 (C-Ph), 128.4 (C-Ph), 141.5 (C-Ph), 169.8 ($C{=}O^{Ac}$), 169.8 ($C{=}O^{Ac}$), 170.6 ($C{=}O^{Ac}$).

IR (ATR): ν (cm^{-1}) = 3061, 3025, 2961, 2933, 2858, 1602, 1573, 1496.

MS (ESI): *m*/*z* (%) = 401.2 (100) $[M+Na]^+$.

$C_{20}H_{26}O_7$ (376.40) calc.: 401.1571
found: 401.1574, $[M+Na]^+$ (ESI-HRMS).

2-(2-Trimethylsilylethyl)-3,4,6-tri-*O*-acetylgalactal (14e)

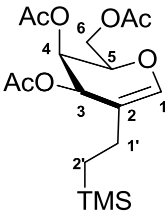

The reaction was performed according to general procedure **cb**. To a solution of compound **11b** (40.3 mg, 0.109 mmol, 1.0 equiv) in a mixture of MeOH:CH_2Cl_2:EtOAc (3:1:1, 5 mL) was added Perlman's catalyst (10 mg). The reaction was stirred overnight at room temperature. After column chromatography on silica gel (pentane/EtOAc = 9:1 → pentane/EtOAc = 3:1) the desired compound **14e** (35.0 mg, 87%) was obtained as a clear oil.

Analytical data of compound **14e**:

R$_f$: 0.56 (hexane/EtOAc = 3:1).

$[\alpha]_D^{20}$ = +25.3° (c = 0.17, $CHCl_3$).

^{1}H-NMR (300 MHz, CD_3OD): δ = –0.06–0.02 (m, 9H, 3 × CH_3^{TMS}), 0.43–0.50 (m, 1H, 2'-H), 0.55–0.65 (m, 1H, 2'-H), 1.81–2.01 (m, 2H, 1'-H), 2.04 (s, 3H, CH_3^{Ac}), 2.08 (s, 3H, CH_3^{Ac}), 2.11 (s, 3H, CH_3^{Ac}), 4.17–4.30 (m, 3H, 4-H, 6-H), 5.42 (dd, *J* = 1.6 Hz, 4.9 Hz, 1H, 3-H), 5.66 (d, *J* = 4.7 Hz, 5-H) 6.30 (s, 1H, 1-H).

^{13}C-NMR (125 MHz, CD_3OD): δ = –1.7 (CH_3^{TMS}), 14.9 (C-2'), 20.7 (CH_3^{Ac}), 20.8 (CH_3^{Ac}), 20.9 (CH_3^{Ac}), 22.8 (C-1'), 62.0 (C-6), 64.6 (C-3*), 64.7 (C-4*), 72.5 (C-5), 112.6 (C-2), 139.7 (C-1), 170.0 (C=O^{Ac}), 170.4 (C=O^{Ac}), 170.5 (C=O^{Ac}).

IR (ATR): ν (cm^{-1}) = 2952, 2923, 1666, 1438, 1103.

MS (ESI): *m/z* (%) = 395.2 (60) $[M+Na]^+$.

$C_{17}H_{28}O_7Si$ (372.49) calc.: 395.1497

found: 395.1497, $[M+Na]^+$ (ESI-HRMS).

α-1-(2-(3-Methylphenyl)ethyl)-1-deoxy-3,4,6-tri-*O*-benzylglucose (15a)

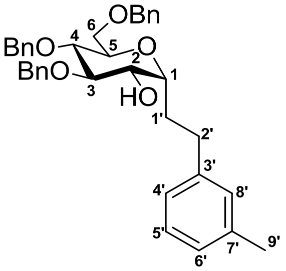

Compound **14b** (17.2 mg, 0.032 mmol, 1.0 equiv) was dissolved in CH_2Cl_2 (1 mL). A solution of DMDO (0.07 M, 0.92 mL, 0.064 mmol, 2.0 equiv) in acetone was added dropwise at 0 °C. The reaction was stirred at 0 °C for 30 min. After the solvent had been removed at 0 °C in vacuo the resulting epoxide was dissolved in THF (3 mL). A solution of $LiBHEt_3$ (1 M, 1.28 mL, 1.28 mmol, 40.0 equiv) in THF was added at 0 °C. After stirring for 3 h at 0 °C, the reaction was stopped by the addition of sat. aq. NH_4Cl solution. The aqueous layer was extracted with CH_2Cl_2 and washed with sat. aq. NaCl solution. The combined organic layers were dried over Na_2SO_4, filtered and concentrated. After column chromatography on silica gel (pentane/EtOAc = 5:1) the desired compound **15a** (5.3 mg, 30%) was obtained as a clear oil.

Analytical data of compound **15a**:

R_f: 0.14 (hexane/EtOAc = 5:1).

$[\alpha]_D^{20}$ = +34.4° (c = 0.48, $CHCl_3$).

^{1}H-NMR (300 MHz, $CDCl_3$): δ = 1.79–2.08 (m, 4H, 1'-H, 2'-H), 2.30 (s, 3H, 9'-H), 2.53–2.63 (m, 1H, 4-H), 2.70–2.79 (m, 1H, 3-H), 3.61–3.82 (m, 3H, 5-H, 6-H, 6-H'), 3.89–4.01 (m, 2H, 1-H, 2-H), 4.49–4.71 (m, 6H, CH_2-Bn), 6.95–7.00 (m, 3H, 4'-H, 6'-H, 8'-H), 7.20–7.36 (m, 16H, 5'-H, H_{Bn}).

^{13}C-NMR (125 MHz, $CDCl_3$): δ = 21.4 (C-9'), 29.7 (C-2'), 31.6 (C-1'), 68.3 (C-6), 69.9 (C-5*), 71.4 (CH_2-Bn), 73.1 (CH_2-Bn), 73.1 (CH_2-Bn), 73.3 (C-2*), 73.5 (C-4*), 75.6 (C-3*), 78.5 (C-1*), 125.4 (C-4'), 126.3 (C-6'), 127.5, 127.6, 127.8, 128.1, 128.2, 128.3, 128.4 (C-5', C_{Bn}), 129.2 (C-8'), 137.4, 137.6, 137.9, 138.0 (C-7', $C_{q,Bn}$), 141.8 (C-3').

IR (film): ν (cm^{-1}) = 2605, 2335, 2084, 1950, 1873, 1809, 1728.

MS (ESI): *m/z* (%) = 575.3 (100) $[M+Na]^+$.

$C_{36}H_{40}O_5$ (552.69) calc.: 575.2773

found: 575.2769, $[M+Na]^+$ (ESI-HRMS).

β-1-(2-(3-Methylphenyl)ethyl)-1-deoxy-3,4,6-tri-*O*-benzylglucose (15b)

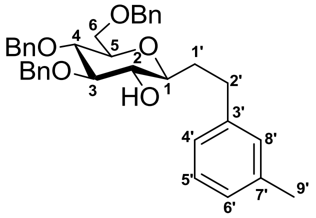

Compound **14b** (8.5 mg, 0.016 mmol, 1.0 equiv) was dissolved in CH_2Cl_2 (2 mL). A solution of DMDO (0.06 M, 0.66 mL, 0.040 mmol, 2.5 equiv) in acetone was added dropwise at −78 °C. The reaction mixture was stirred at −78 °C for 30 min. After the solvent had been removed at 0 °C in vacuo the resulting epoxide was dissolved in CH_2Cl_2 (2 mL). A solution of DIBAL-H (1 M, 40 µL, 0.040 mmol, 2.5 equiv) in toluene was added at room temperature. After stirring for 30 min at room temperature, the reaction was stopped by the addition of sat. aq. sodium potassium tartrate solution. The aqueous layer was extracted with CH_2Cl_2 and washed with sat. aq. NaCl solution. The combined organic layers were dried over Na_2SO_4, filtered and concentrated. After column chromatography on silica gel (pentane/EtOAc = 5:1) the desired compound **15b** (3.5 mg, 40%) was obtained as a clear oil.

Analytical data of compound **15b**:

R_f: 0.23 (hexane/EtOAc = 4:1).

$[\alpha]_D^{20}$ = +1.0° (c = 1.78, $CHCl_3$).

^{1}H-NMR (300 MHz, $CDCl_3$): δ = 1.69–1.77 (m, 2H, 1'-H), 2.01–2.11 (m, 2H, 2'-H), 2.29 (s, 3H, 9'-H), 2.59–2.69 (m, 1H, 4-H), 2.78–2.88 (m, 1H, 3-H), 3.13 (dt, *J* = 2.8, 9.1 Hz, 1H, 2-H), 3.29–3.86 (m, 4H, 1-H, 5-H, 6-H, 6-H'), 4.52–4.96 (m, 6H, CH_2-Bn), 6.95–7.00 (m, 3H, 4'-H, 6'-H, 8'-H), 7.11–7.38 (m, 16H, 5'-H, H_{Bn}).

^{13}C-NMR (125 MHz, $CDCl_3$): δ = 21.4 (C-9'), 31.4 (C-2'), 33.6 (C-1'), 69.0 (C-6), 73.5 (C-5*), 73.9 (CH_2-Bn), 74.7 (CH_2-Bn), 75.1 (CH_2-Bn), 78.3 (C-2), 78.6 (C-4), 79.0 (C-3*), 86.9 (C-1*), 125.4 (C-4'), 126.3 (C-6'), 127.5, 127.6, 127.7, 127.8, 127.9, 128.2, 128.3, 128.6 (C-5', C_q), 129.3 (C-8'), 137.7, 137.8, 138.0, 138.1 (C-7', C_q), 141.9 (C-3').

IR (film): ν (cm^{-1}) = 1952, 1875, 1808, 1547.

MS (ESI): *m*/*z* (%) = 575.28 (100) $[M+Na]^+$.

$C_{36}H_{40}O_5$ (552.69) calc.: 575.27680

found: 575.27672, $[M+Na]^+$ (ESI-HRMS).

β-1-Pentyl-3,4,6-tri-*O*-isopropylsilylglucose (15c)

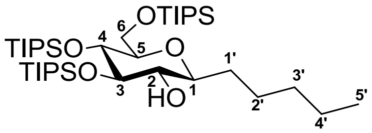

Compound **14c** (26.0 mg, 0.038 mmol, 1.0 equiv) was dissolved in CH_2Cl_2 (1 mL). A solution of DMDO (0.07 M, 2.72 mL, 0.190 mmol, 5.0 equiv) in acetone was added dropwise at −78 °C. The reaction mixture was stirred at −78 °C for 20 min before it was allowed to warm to 0 °C for 15 min. After the solvent had been removed at 0 °C in vacuo the resulting epoxide was dissolved in CH_2Cl_2 (1 mL). A solution of DIBAL-H (1 M, 190 µL, 0.19 mmol, 5.0 equiv) in toluene was added at room temperature. After stirring for 30 min at room temperature, the reaction was stopped by the addition of sat. aq. sodium potassium tartrate solution. The aqueous layer was extracted with CH_2Cl_2 and washed with sat. aq. NaCl solution. The combined organic layers were dried over Na_2SO_4, filtered and concentrated. After column chromatography on silica gel (pentane/EtOAc = 30:1) the desired compound **15c** (3.5 mg, 13%) was obtained as a clear oil.

Analytical data of compound **15c**:

R_f: 0.33 (hexane/EtOAc = 30:1).

$[\alpha]_D^{20}$ = +14.1° (c = 0.32, $CHCl_3$).

^{1}H-NMR (300 MHz, $CDCl_3$): δ = 0.84–0.89 (m, 3H, 5'-H), 0.95–1.15 (m, 63H, TIPS-H), 1.20–1.35 (m, 4H, 2'-H, 3'-H), 1.40–1.53 (m, 2H, 4'-H), 1.60–1.73 (m, 2H, 1'-H), 3.33–3.43 (m, 2H, 2-H, 4-H), 3.76–3.84 (m, 3H, 1-H, 2-H, 6-H'), 3.90–4.02 (m, 1H, 3-H), 4.04 (d, *J* = 3.7 Hz, 1H, 5-H), 4.18 (dd, *J* = 1.1, 3.9 Hz, 1H, 6-H), 4.54–4.57 (m, 1H, OH).

^{13}C-NMR (125 MHz, $CDCl_3$): δ = 12.1, 12.3, 12.4 (TIPS-CH), 14.1 (C-5'), 18.0, 18.1, 18.2 (TIPS-CH3), 22.7 (C-4'), 25.4 (C-2'), 32.0 (C-1'), 35.1 (C-3'), 65.3 (C-6), 71.6 (C-4), 75.5 (C-2), 76.3 (C-3), 78.4 (C-1), 82.6 (C-5).

IR (film): ν (cm^{-1}) = 3478, 2756, 2725, 2623, 2563, 2243, 1937, 1730, 1548.

MS (ESI): *m/z* (%) = 725.6 (100) $[M+Na]^+$.

$C_{38}H_{82}O_5Si_3$ (703.31) calc.: 725.5362

found: 725.5385, $[M+Na]^+$ (ESI-HRMS).

1-Phenylethynyl-3,4,6-tri-*O*-isopropylsilylglucal (9a)

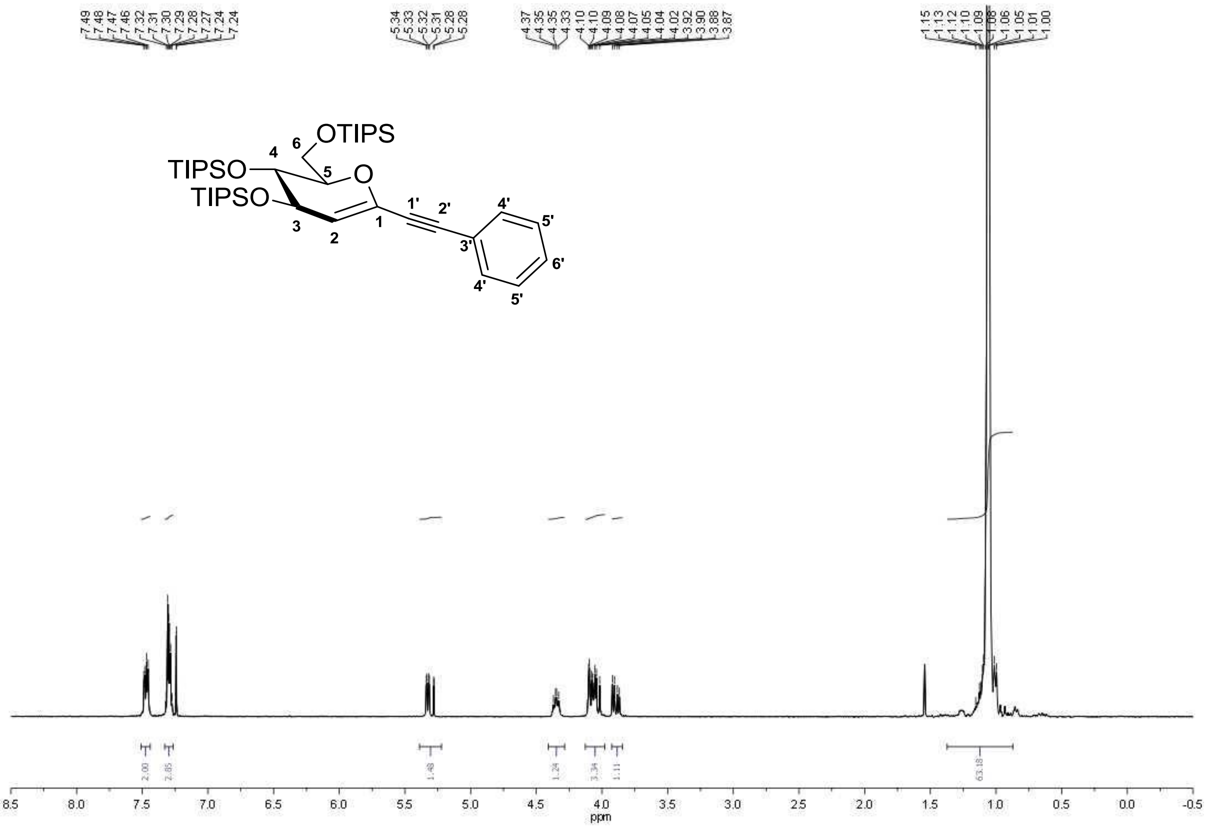

^{1}H NMR (300 MHz, $CDCl_3$)

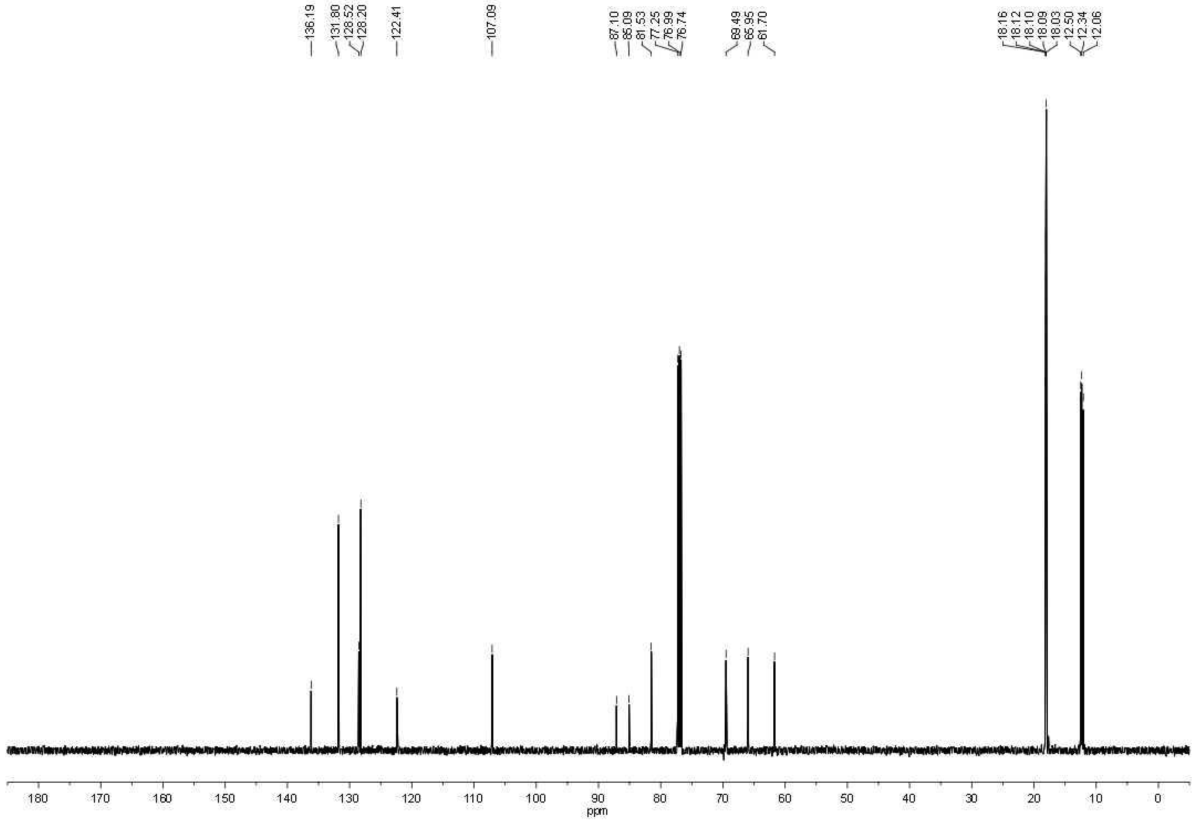

^{13}C NMR (125 MHz, $CDCl_3$)

1-(4-Methylphenylethynyl)-3,4,6-tri-*O*-isopropylsilylglucal (9b)

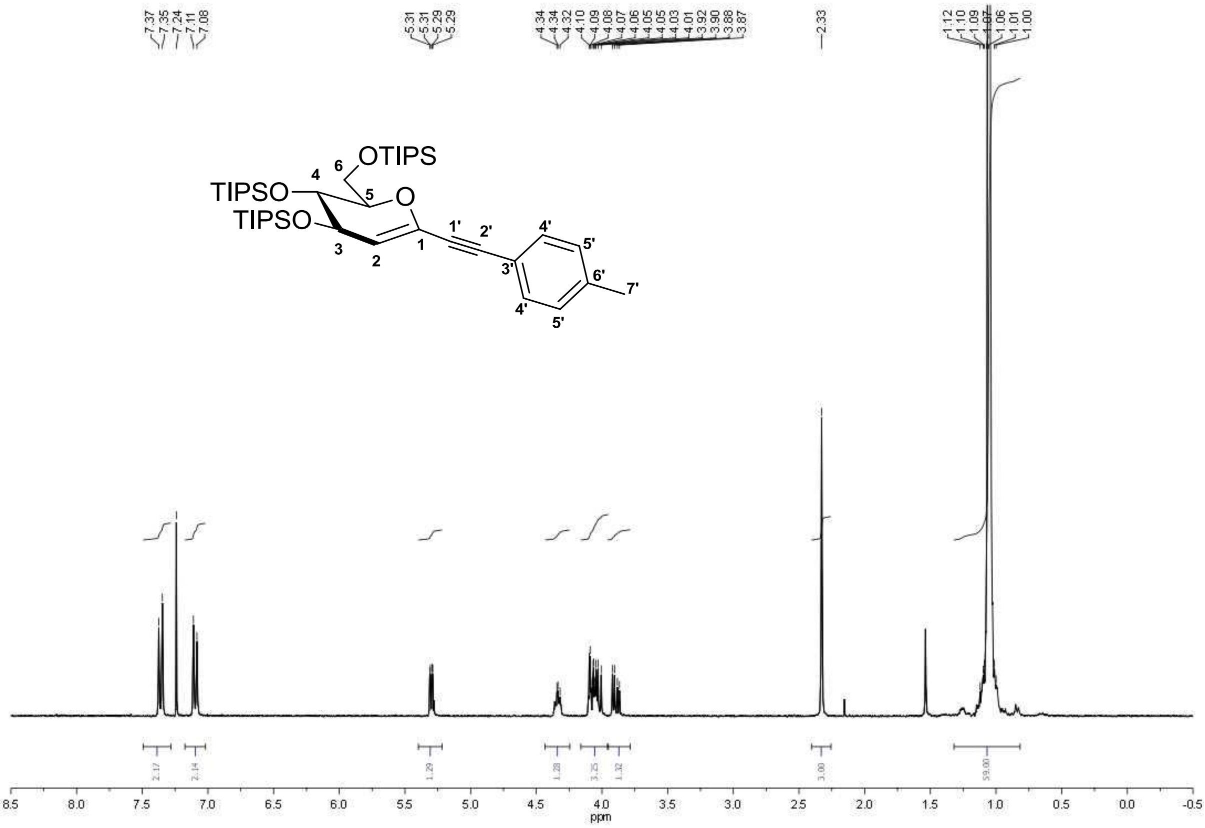

^{1}H NMR (300 MHz, $CDCl_3$)

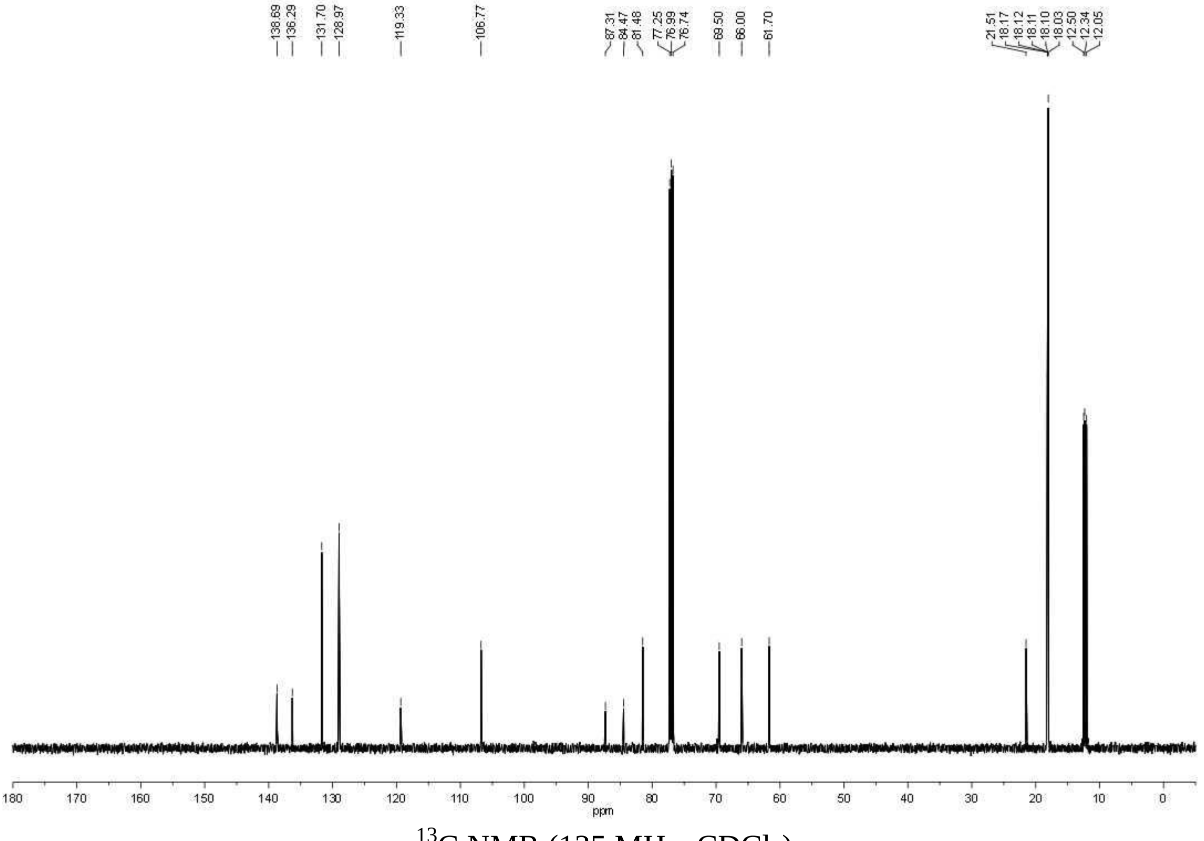

^{13}C NMR (125 MHz, $CDCl_3$)

1-(4-Methoxyphenylethynyl)-3,4,6-tri-*O*-isopropylsilylglucal (9c)

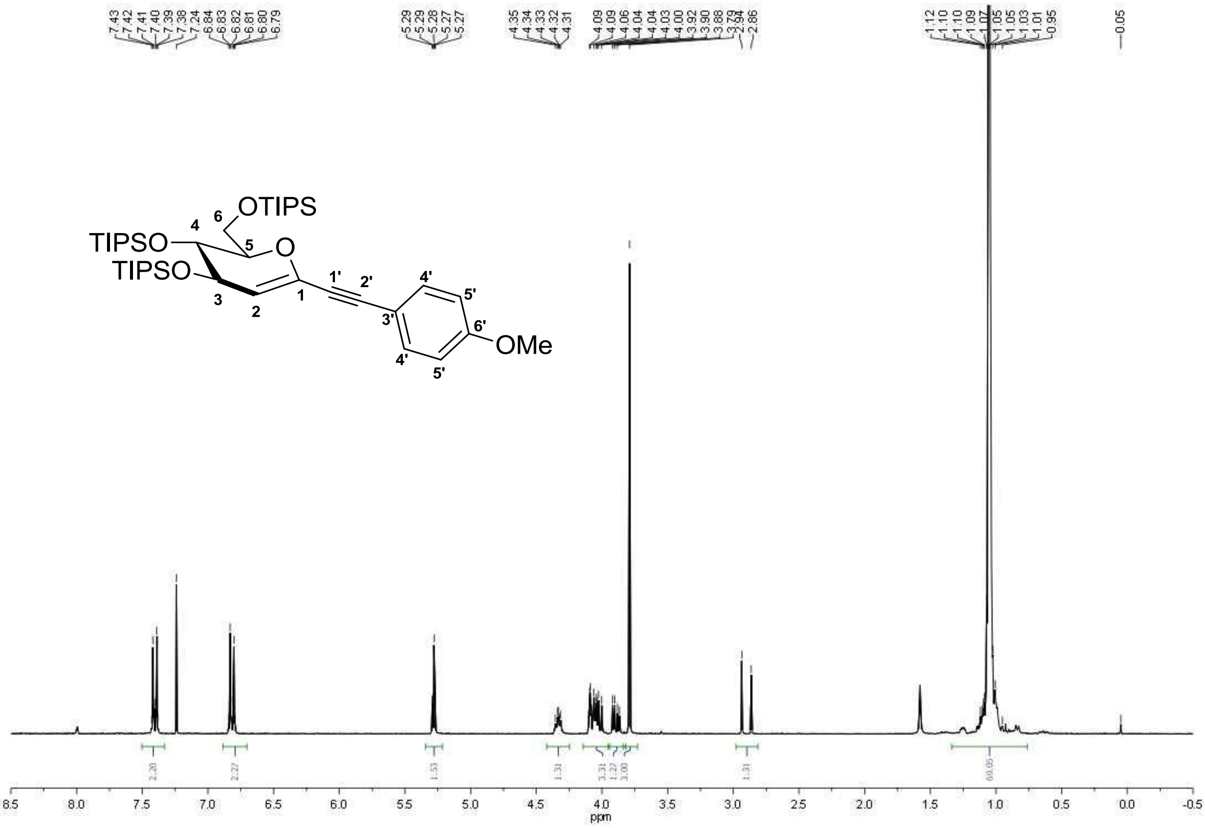

^{1}H NMR (300 MHz, $CDCl_3$)

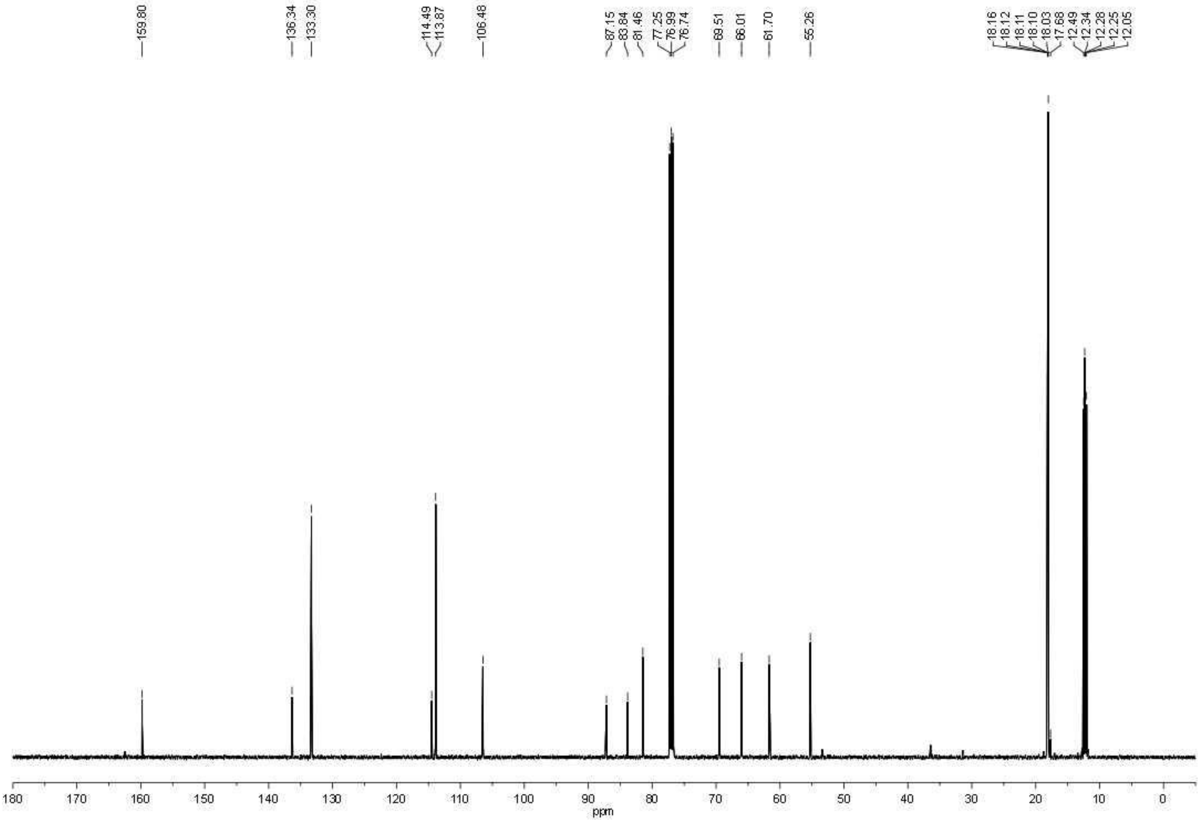

^{13}C NMR (125 MHz, $CDCl_3$)

1-(4-Fluorophenylethynyl)-3,4,6-tri-*O*-isopropylsilylglucal (9d)

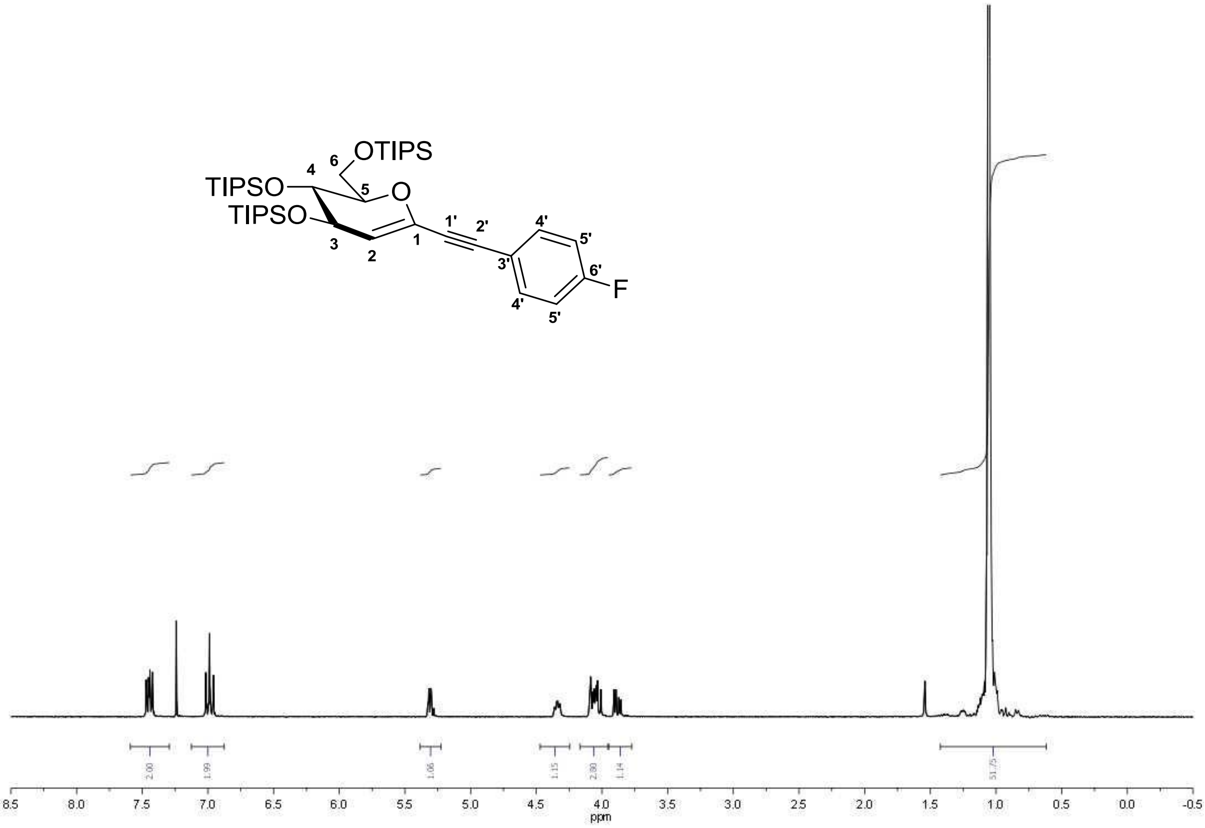

^{1}H NMR (300 MHz, CDCl$_3$)

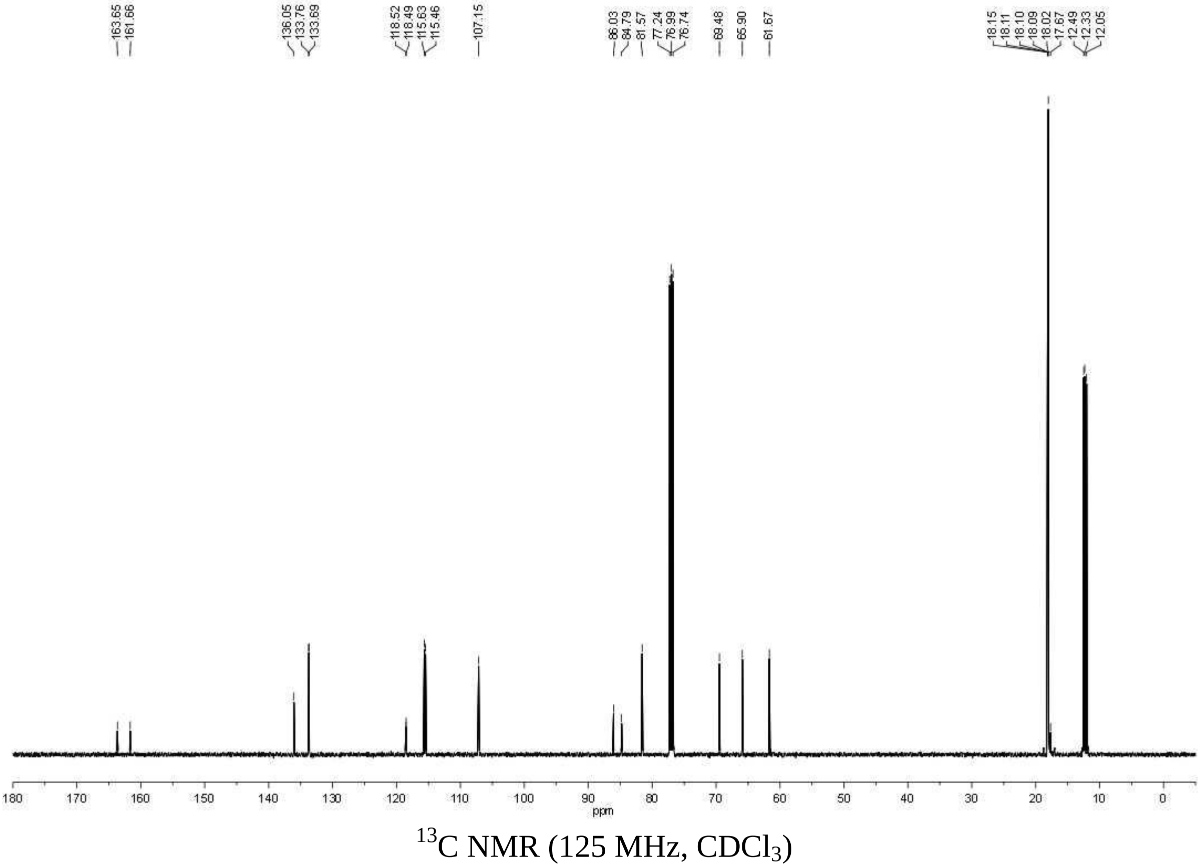

^{13}C NMR (125 MHz, CDCl$_3$)

1-(3-Methylphenylethynyl)-3,4,6-tri-*O*-isopropylsilylglucal (9e)

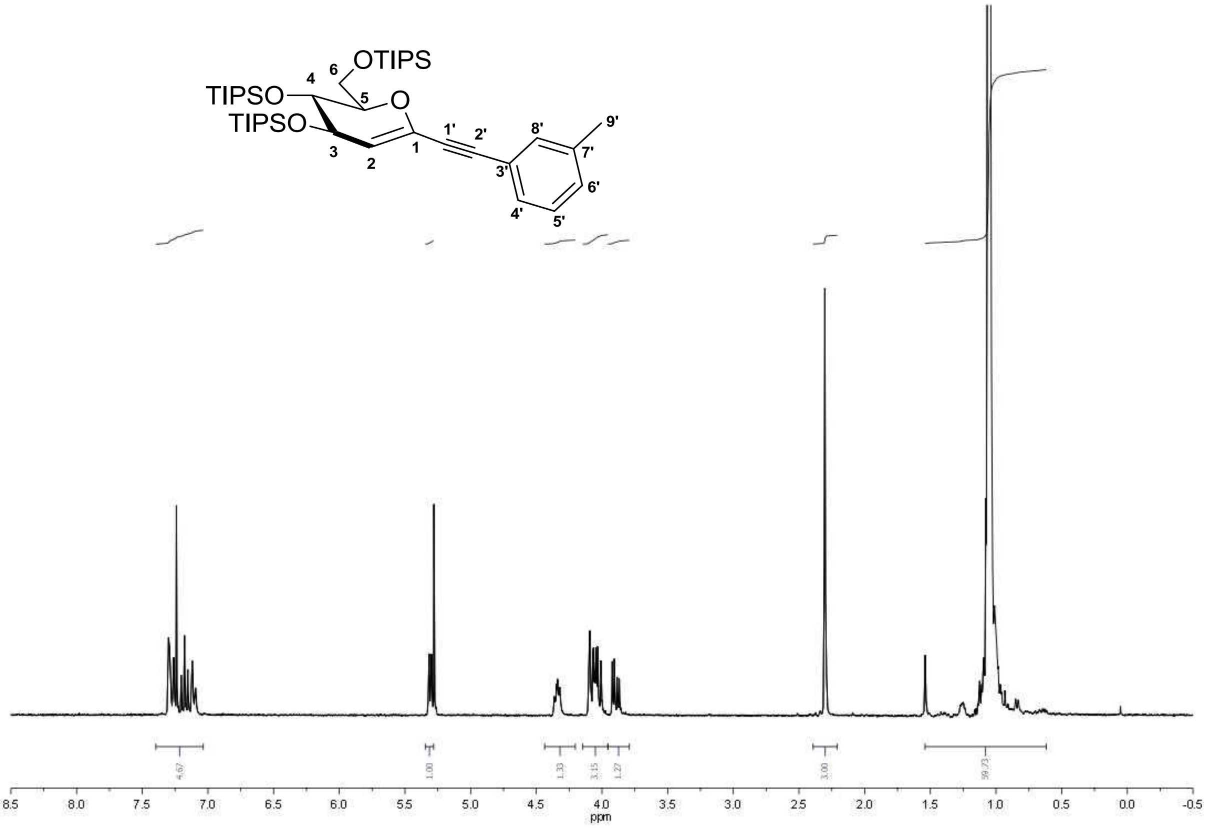

^{1}H NMR (300 MHz, $CDCl_3$)

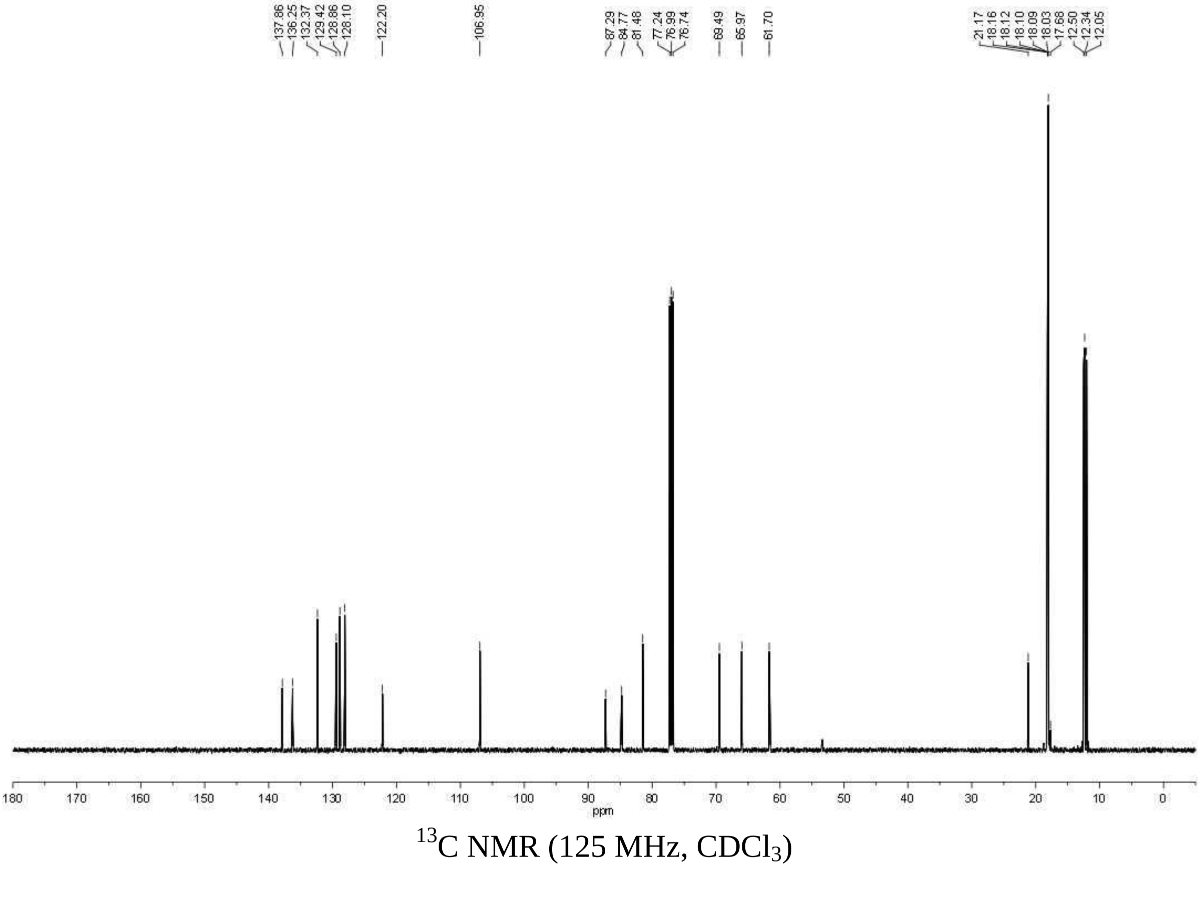

^{13}C NMR (125 MHz, $CDCl_3$)

1-(Trimethylsilylethynyl)-3,4,6-tri-*O*-isopropylsilylglucal (9f)

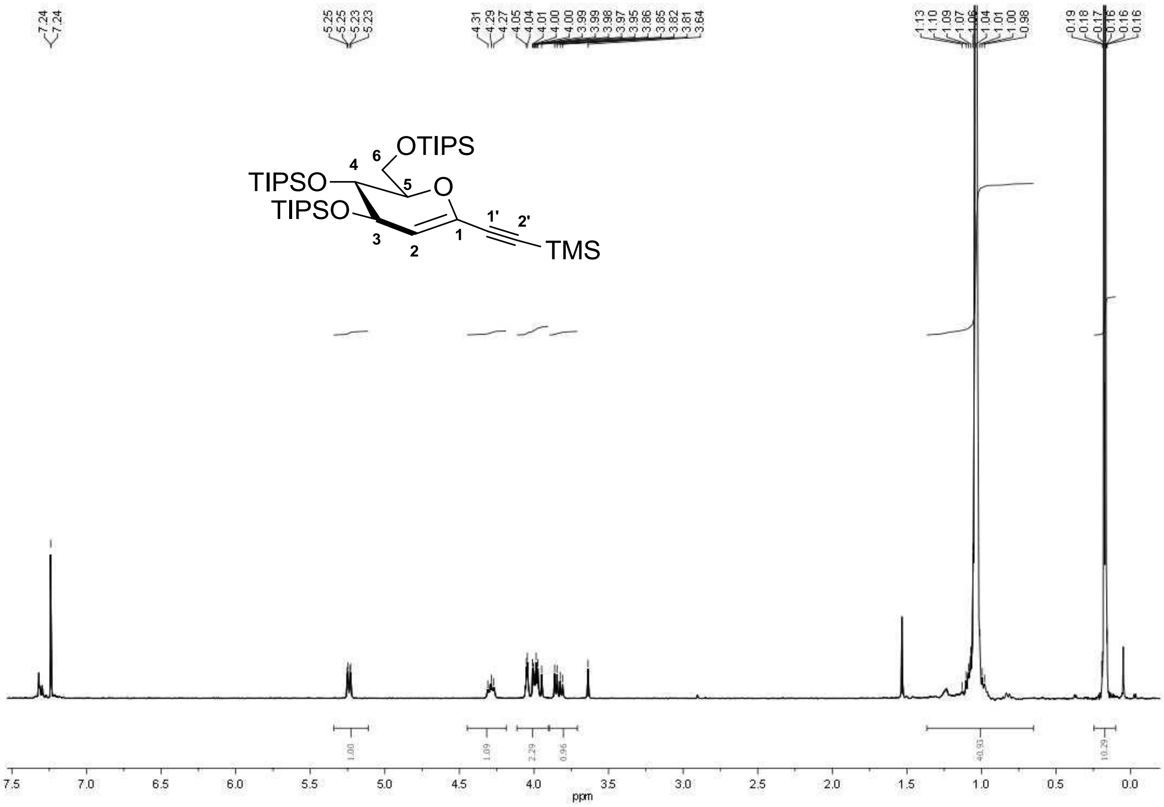

^{1}H NMR (300 MHz, $CDCl_3$)

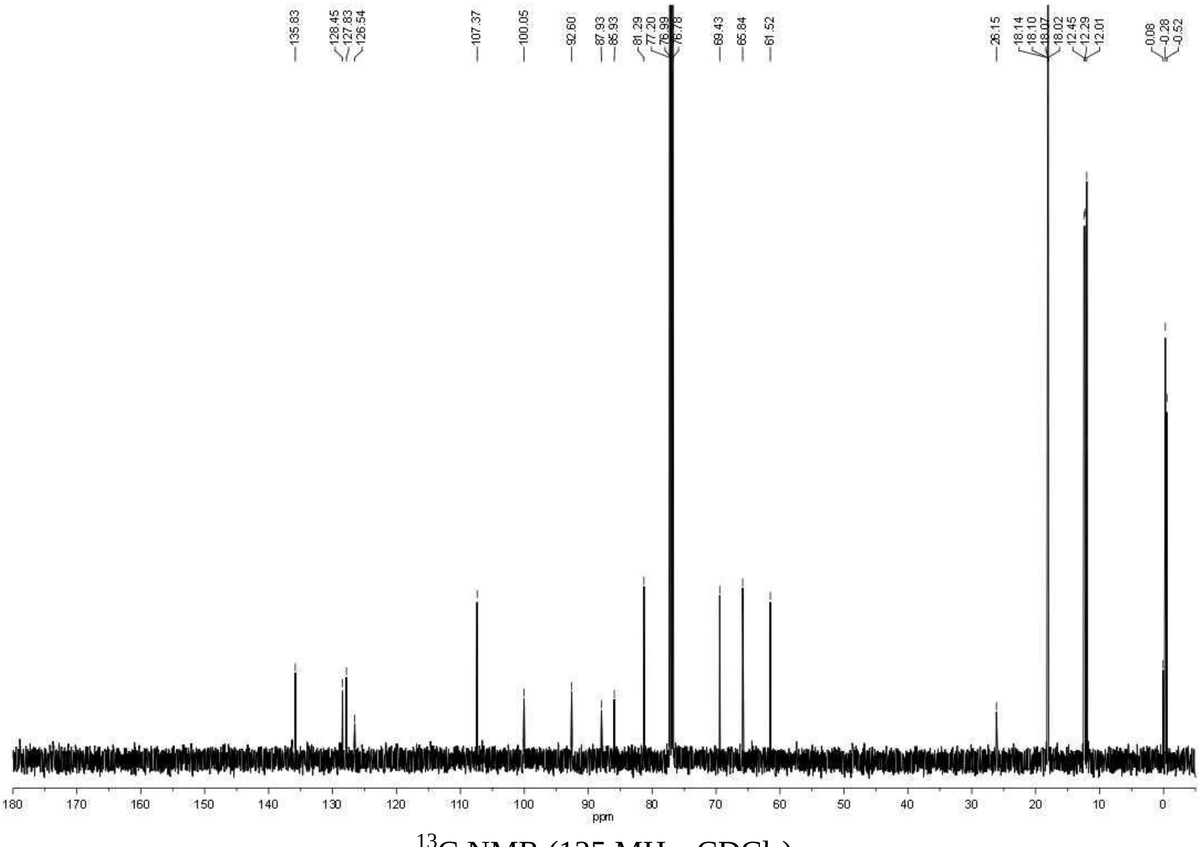

^{13}C NMR (125 MHz, $CDCl_3$)

1-Hexyn-1-yl-3,4,6-tri-*O*-isopropylsilylglucal (9g)

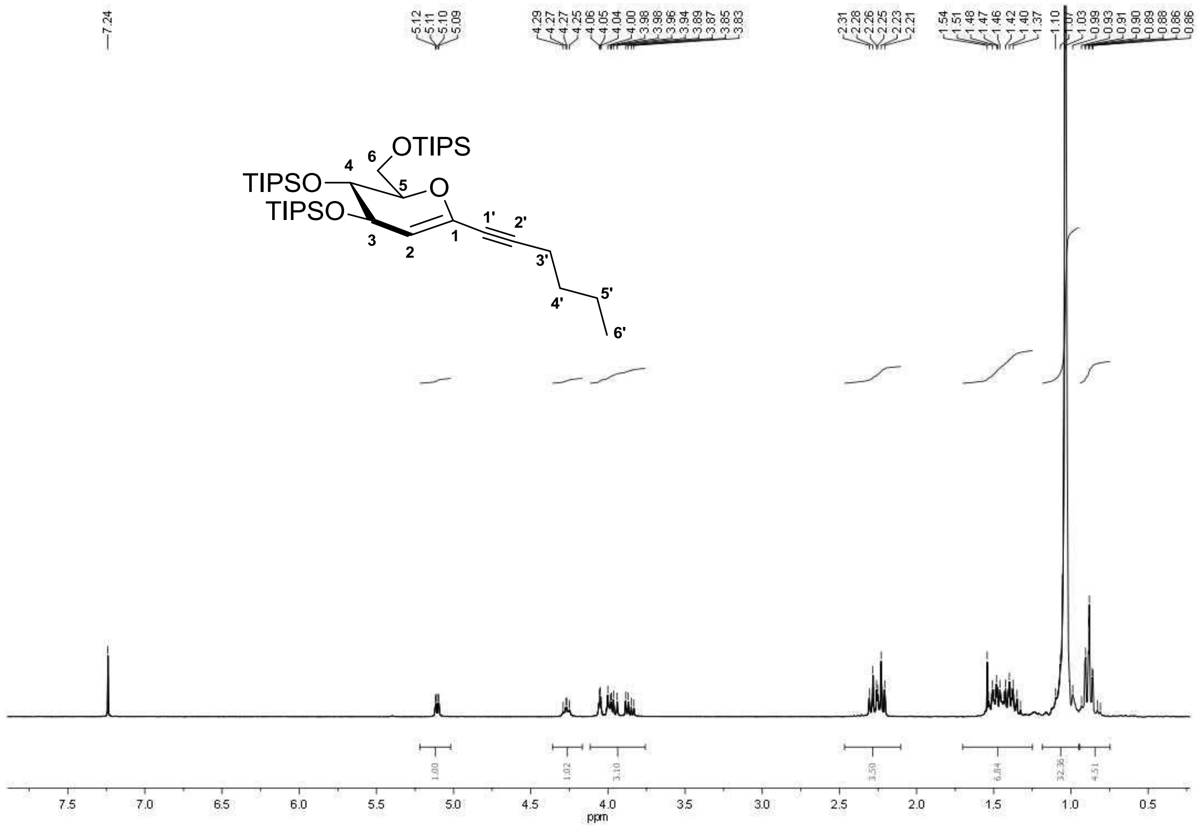

^{1}H NMR (300 MHz, $CDCl_3$)

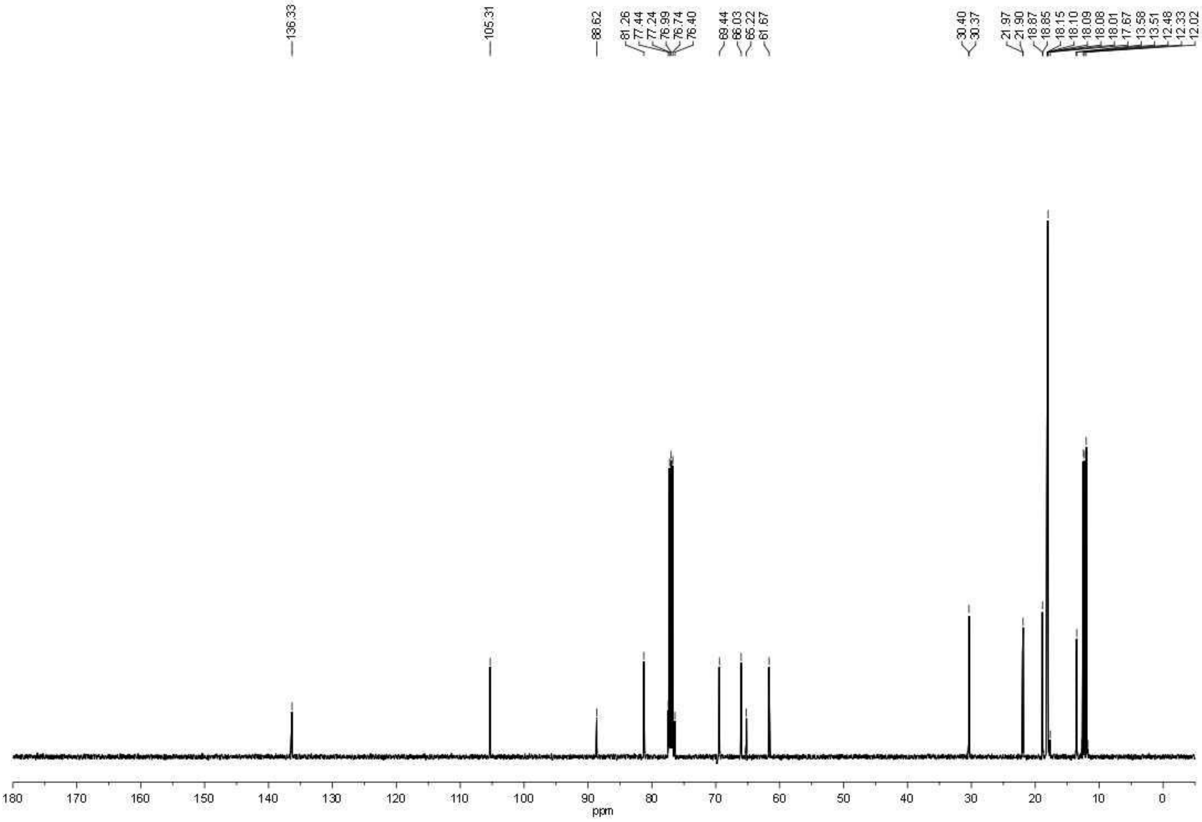

^{13}C NMR (125 MHz, $CDCl_3$)

1-Pentyn-1-yl-3,4,6-tri-*O*-isopropylsilylglucal (9h)

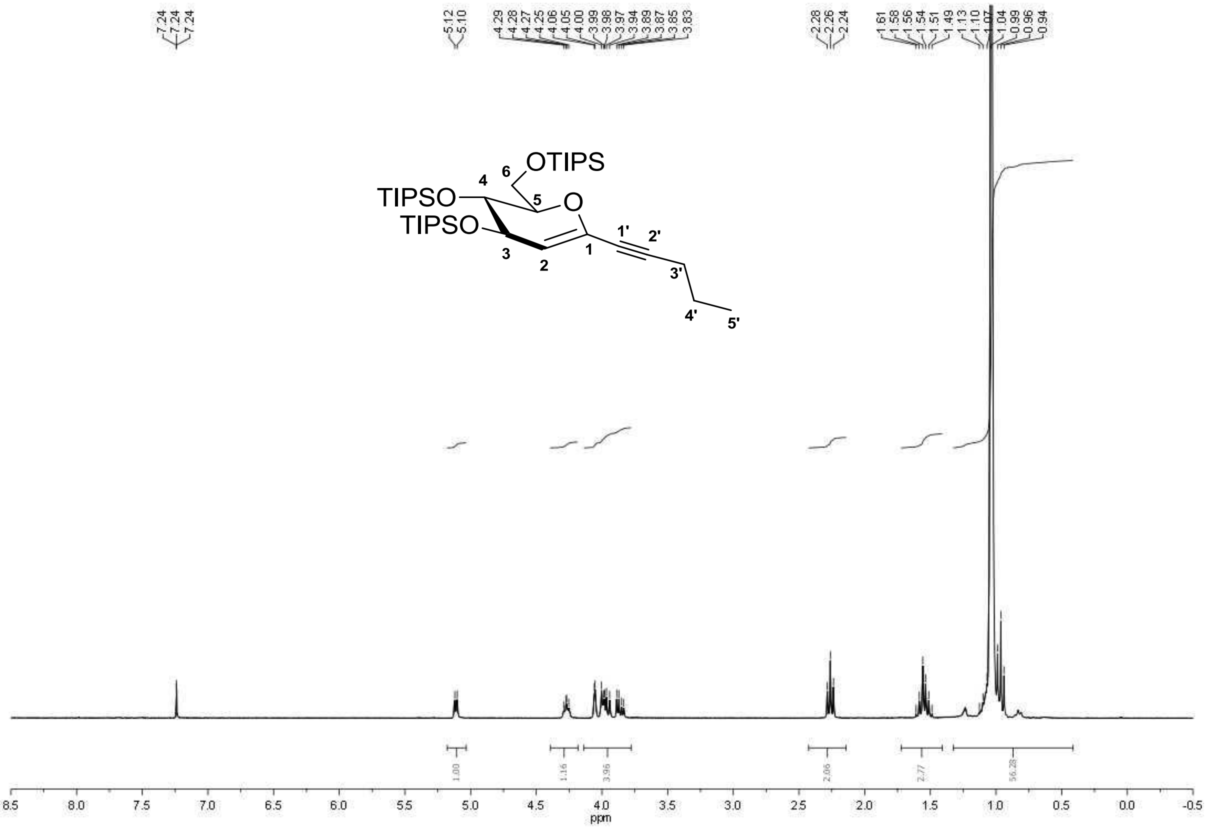

^{1}H NMR (300 MHz, $CDCl_3$)

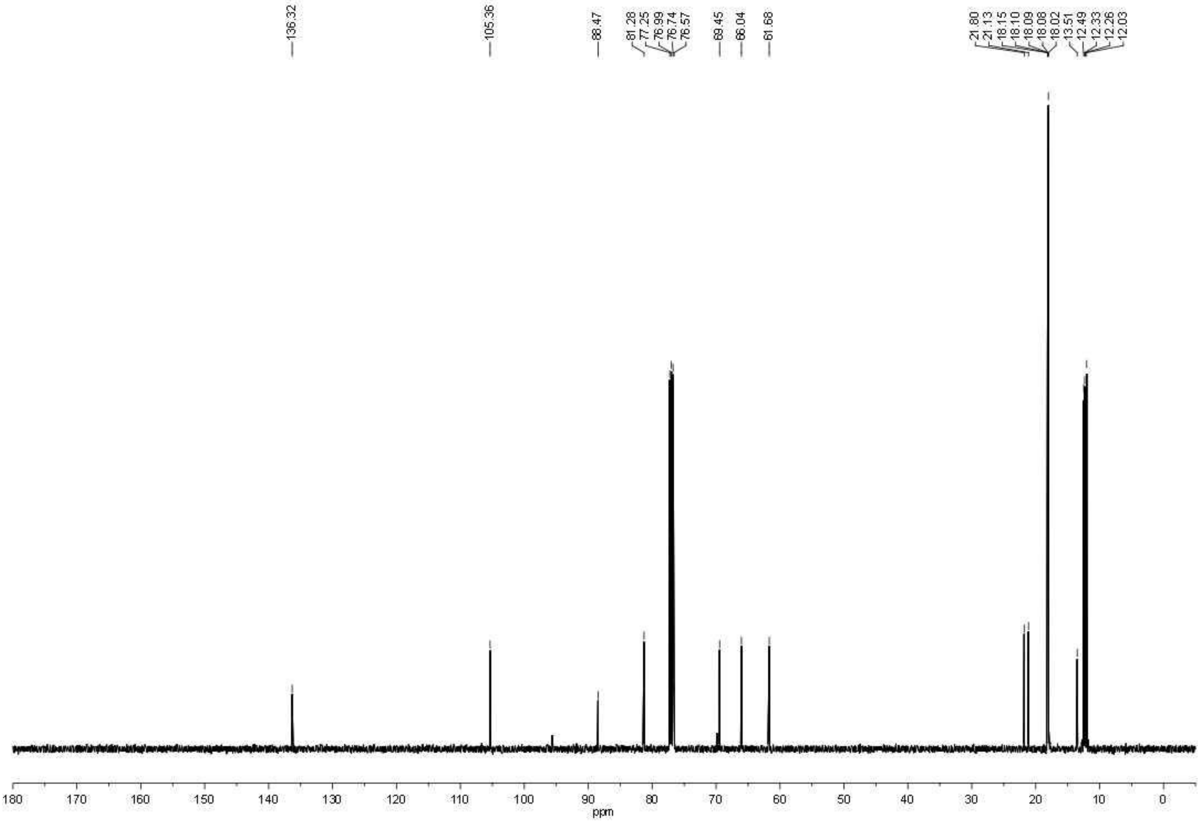

^{13}C NMR (125 MHz, $CDCl_3$)

1-Phenylethynylglucal (9aa)

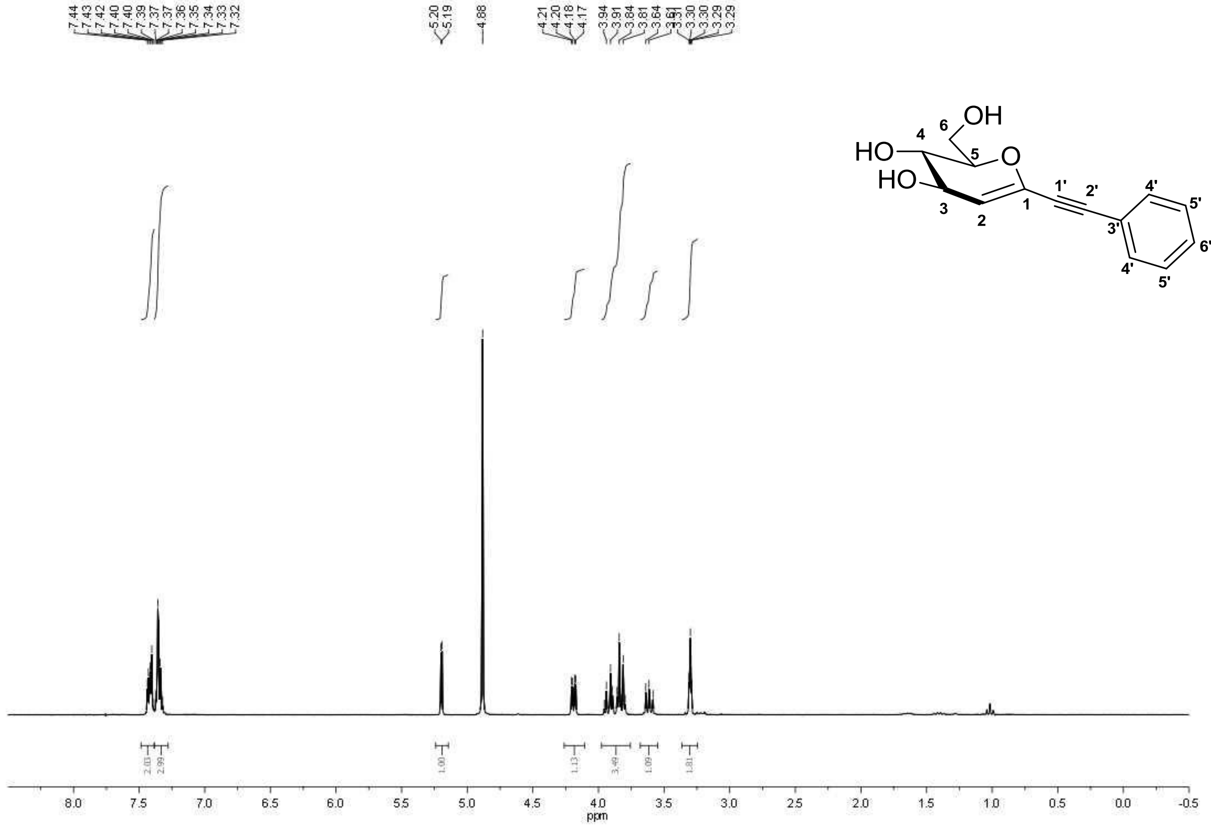

^{1}H NMR (300 MHz, $CDCl_3$)

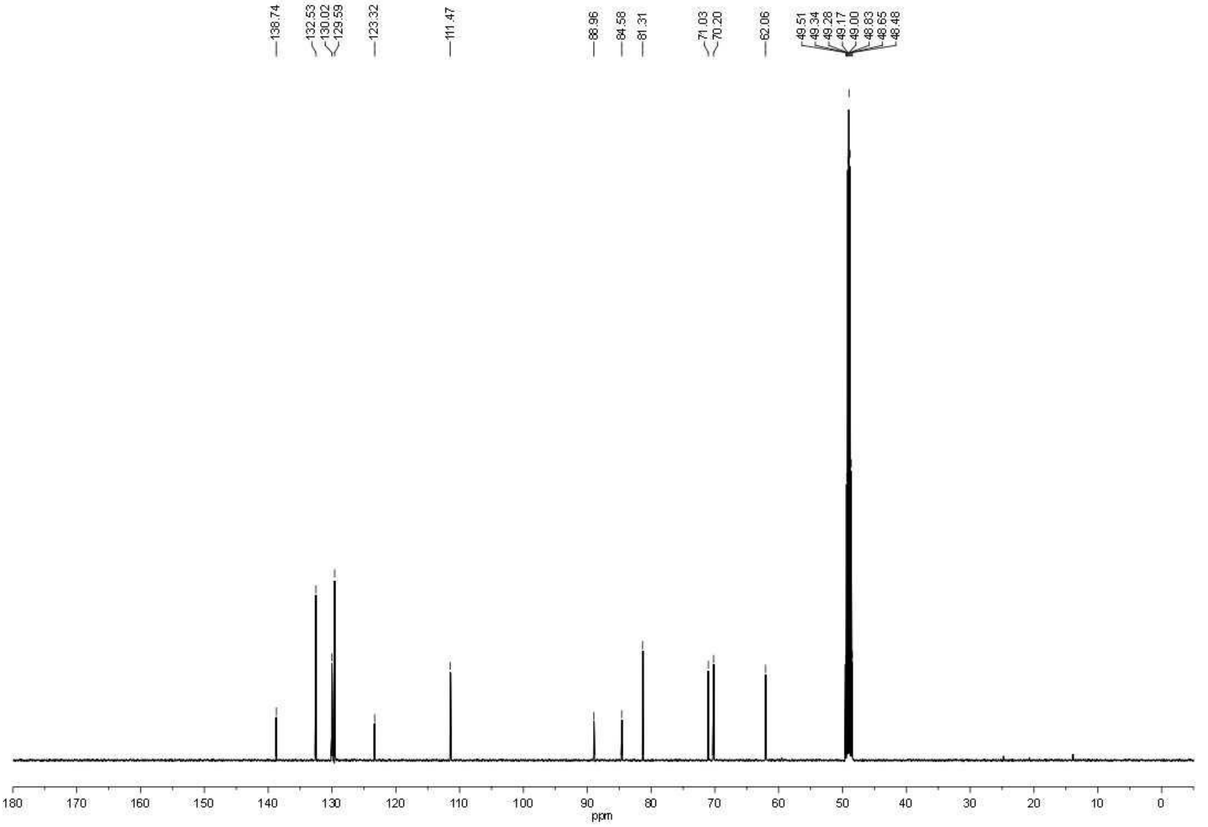

^{13}C NMR (125 MHz, $CDCl_3$)

1-(Phenylethynyl)-3,4,6-tri-*O*-benzylglucal (9ab)

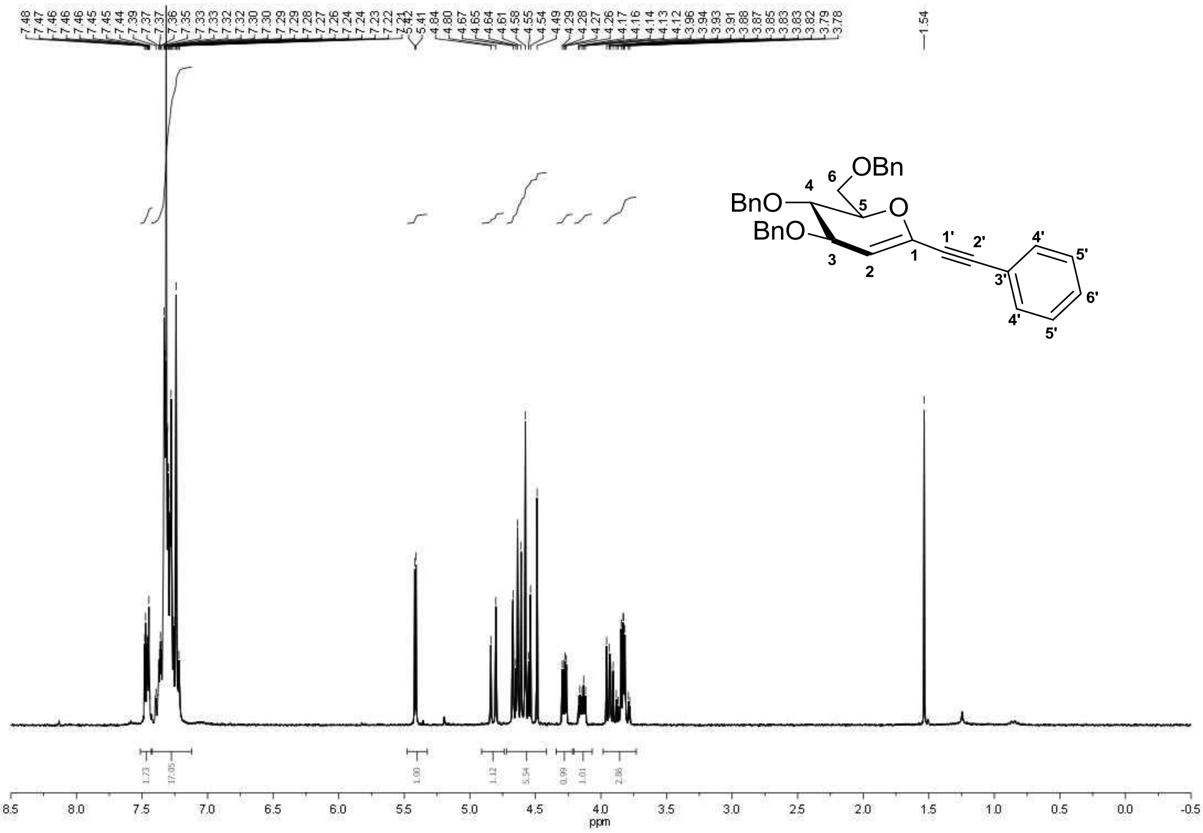

^{1}H NMR (300 MHz, $CDCl_3$)

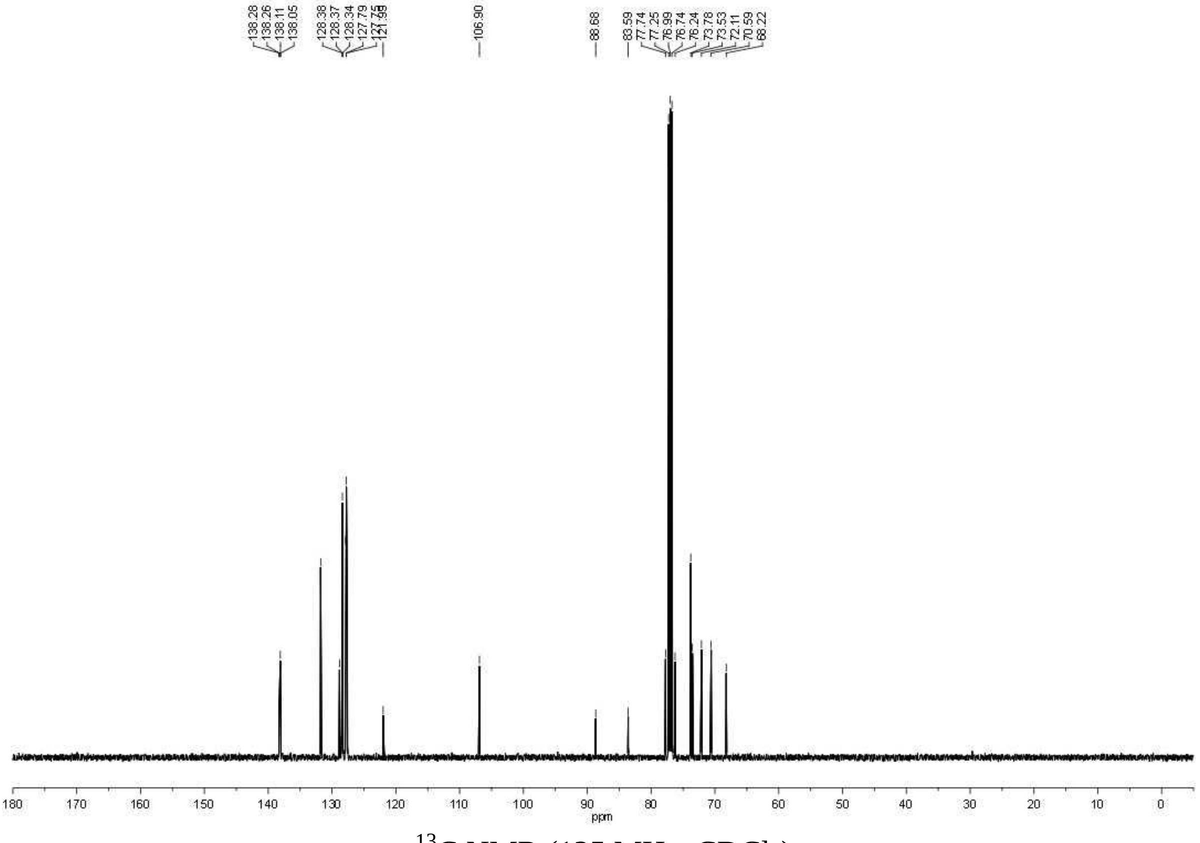

^{13}C NMR (125 MHz, $CDCl_3$)

1-(3-Methylphenylethynyl)-3,4,6-tri-*O*-benzylglucal (9ea)

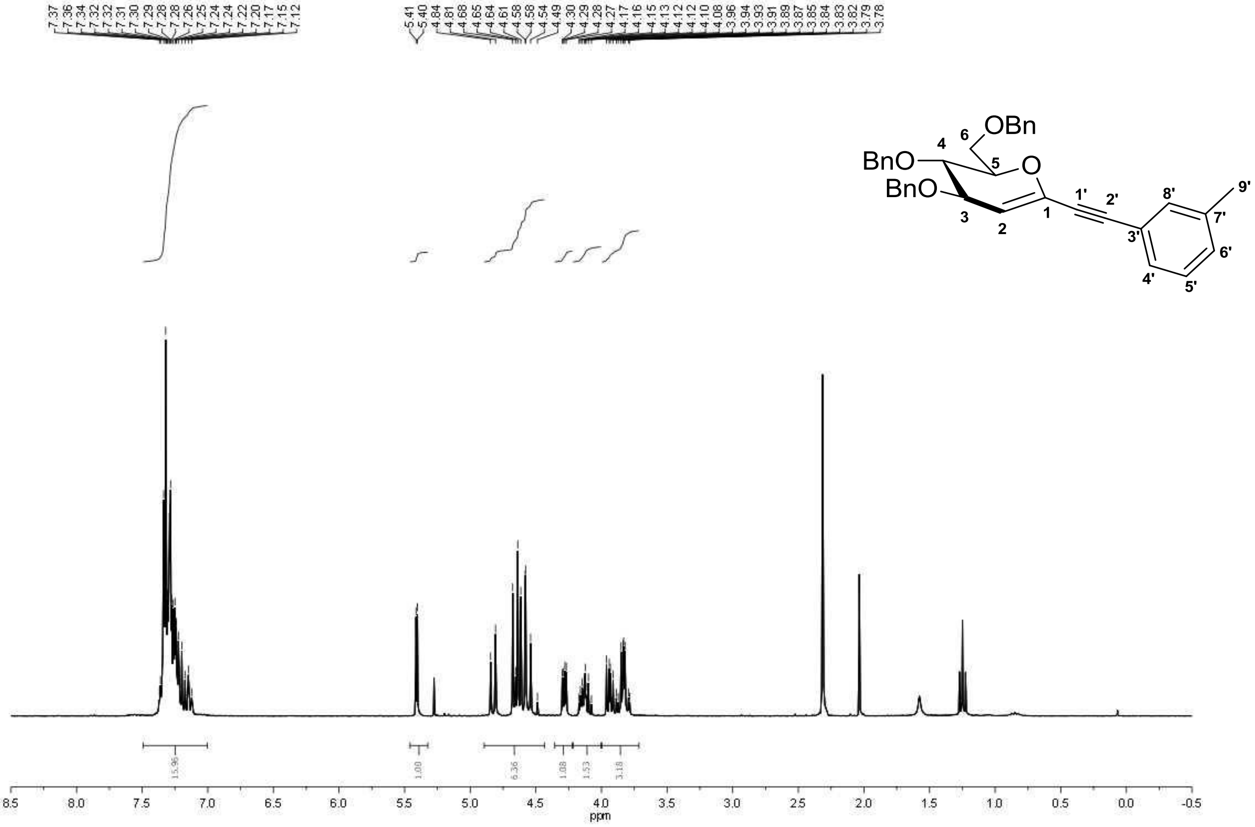

^{1}H NMR (300 MHz, CDCl$_3$)

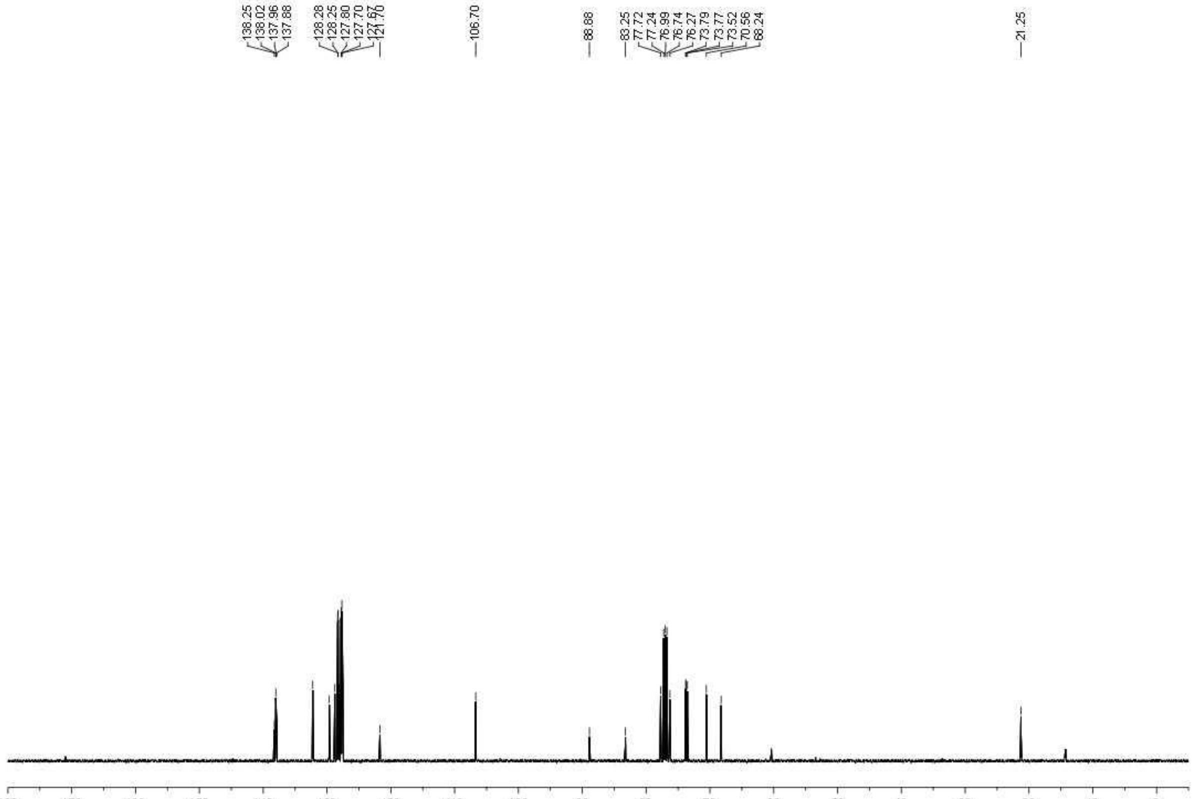

^{13}C NMR (125 MHz, CDCl$_3$)

1-(3-Methylphenylethynyl)glucal (9eb)

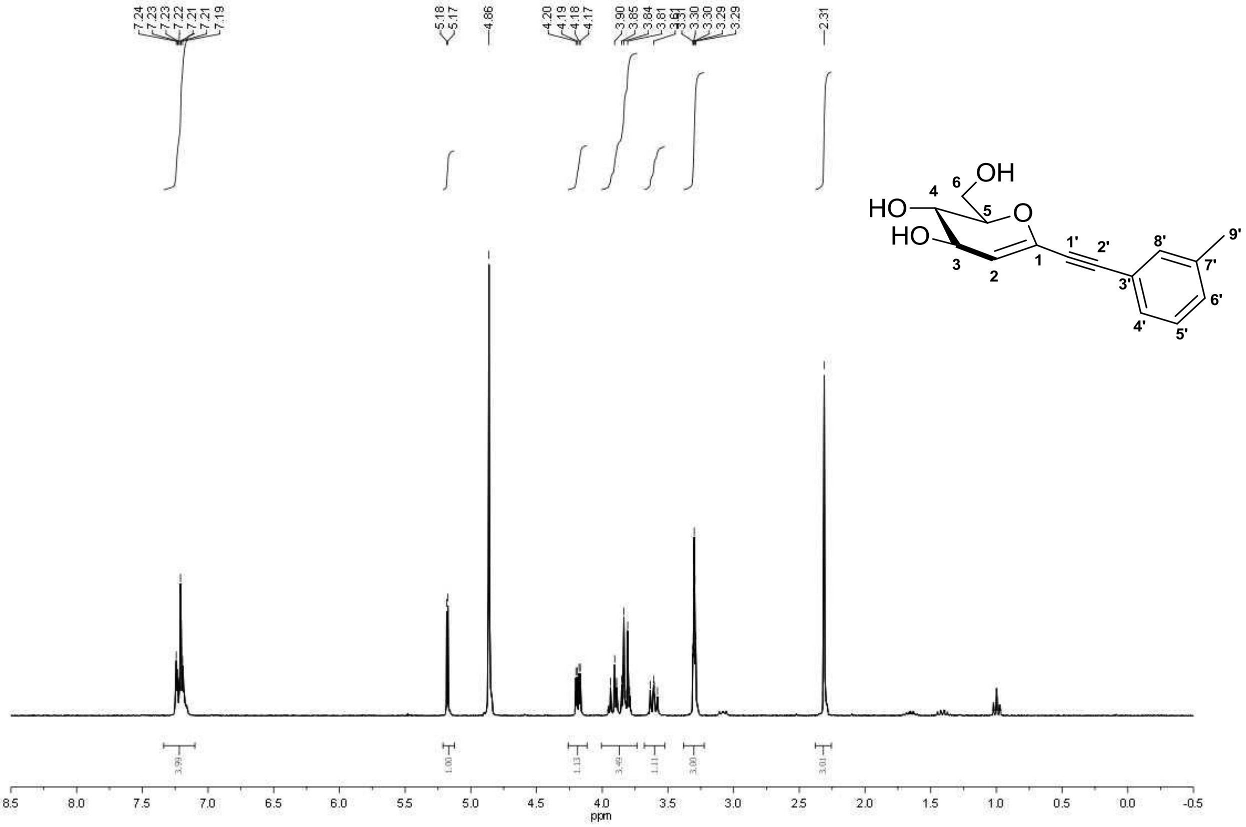

^{1}H NMR (300 MHz, $CDCl_3$)

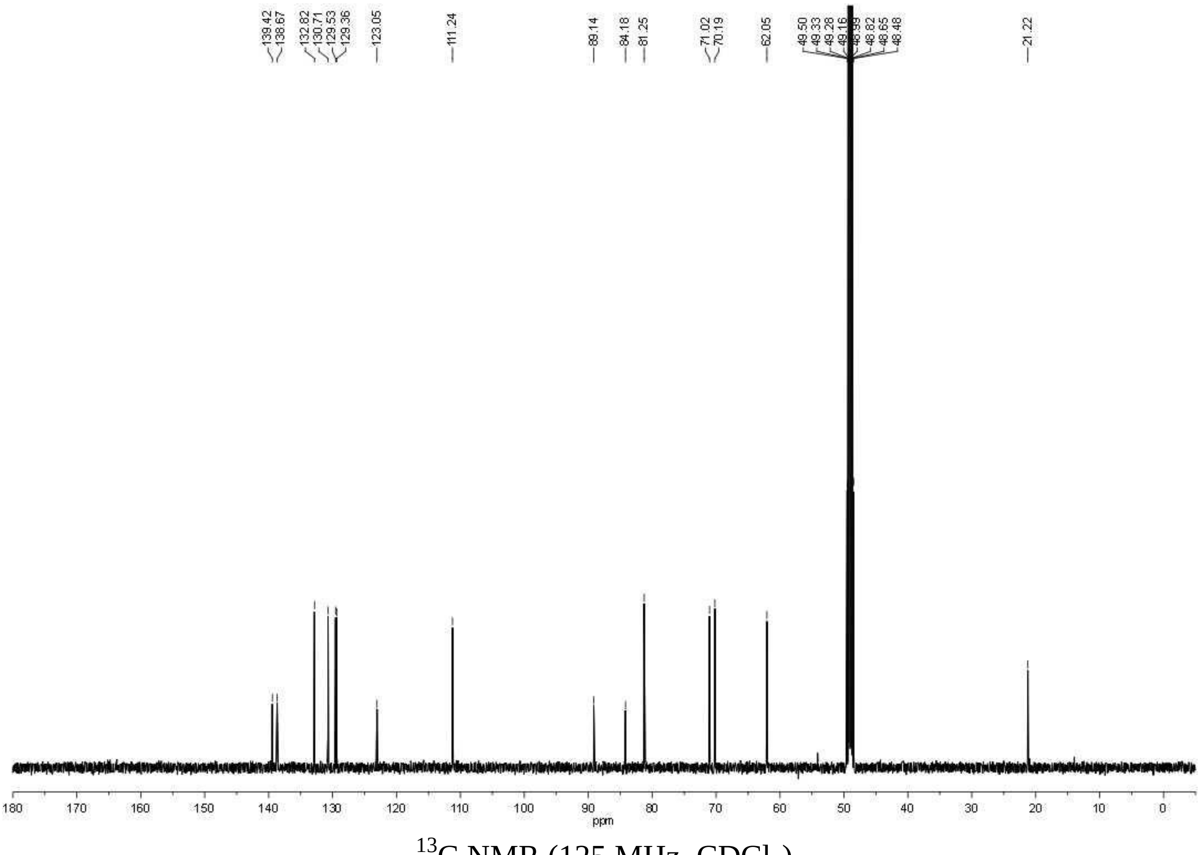

^{13}C NMR (125 MHz, $CDCl_3$)

2-(Phenylethynyl)-3,4,6-tri-*O*-acetylgalactal (11a)

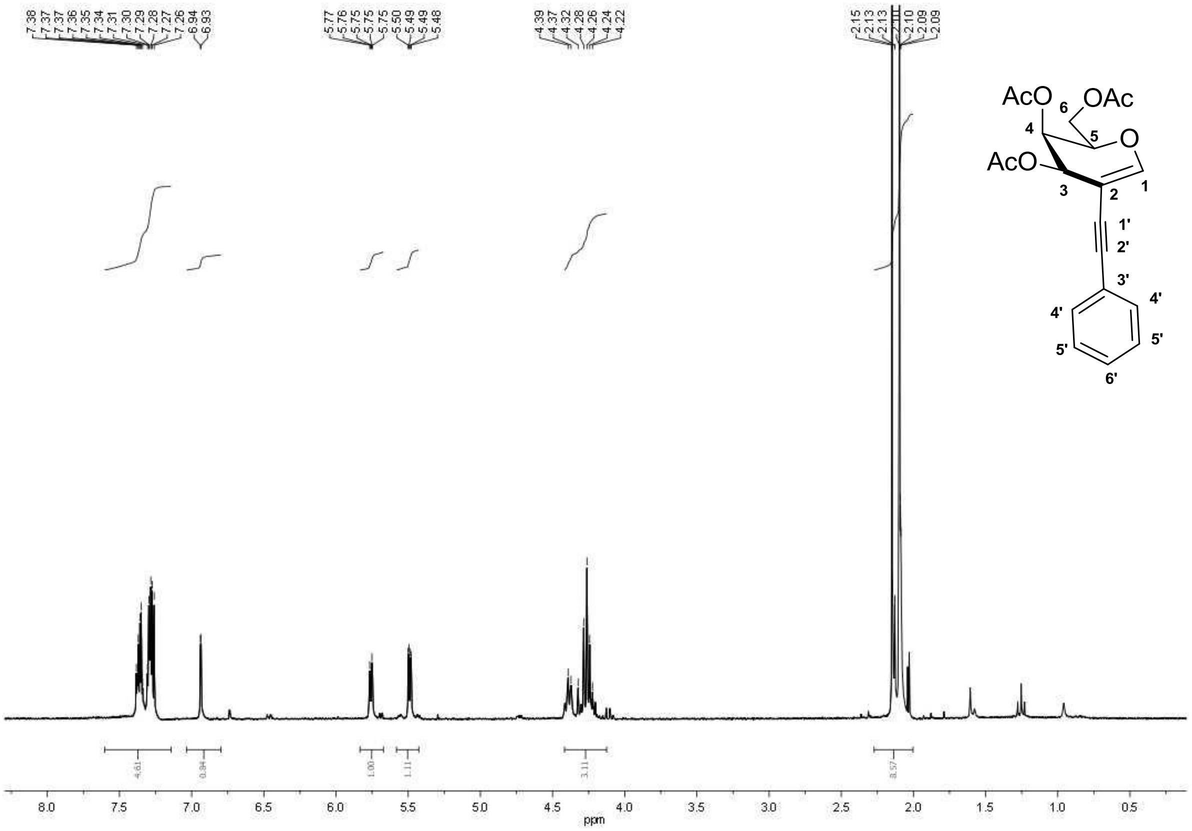

^{1}H NMR (300 MHz, $CDCl_3$)

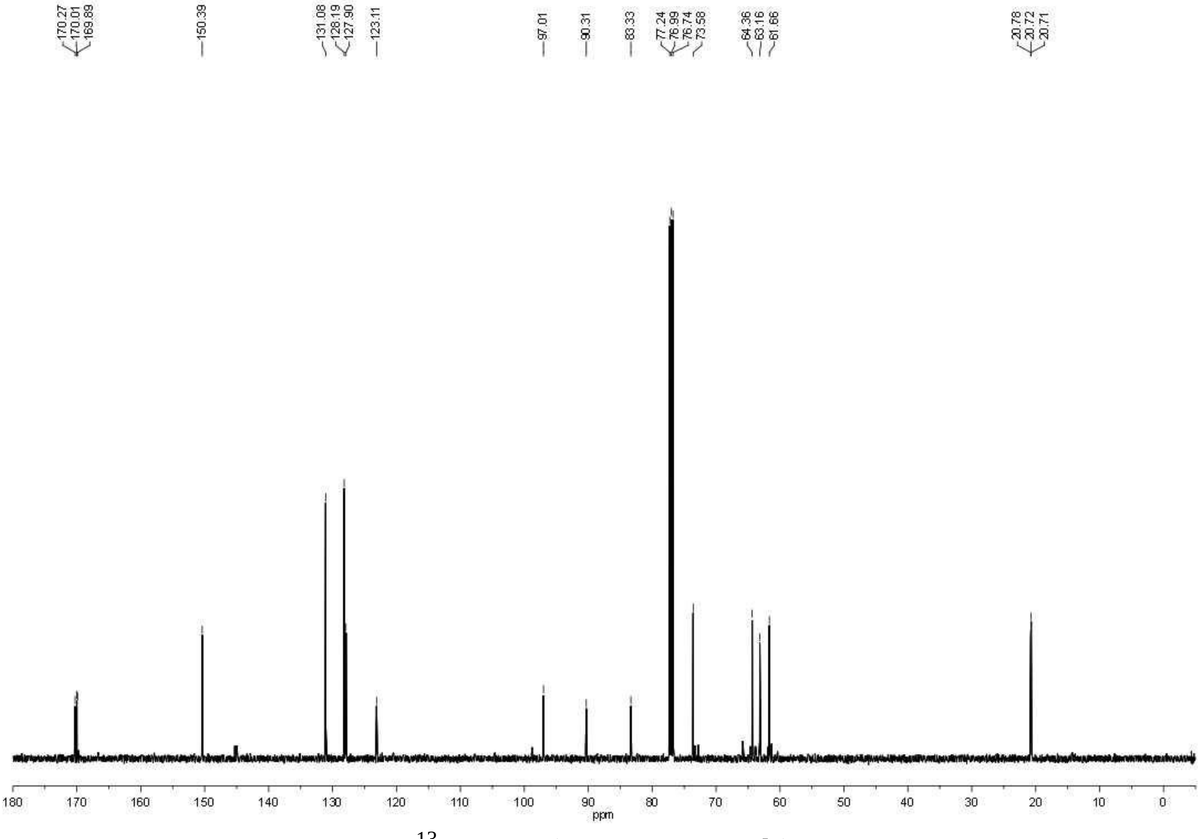

^{13}C NMR (125 MHz, $CDCl_3$)

2-(Trimethylsilylethynyl)-3,4,6-tri-*O*-acetylgalactal (11b)

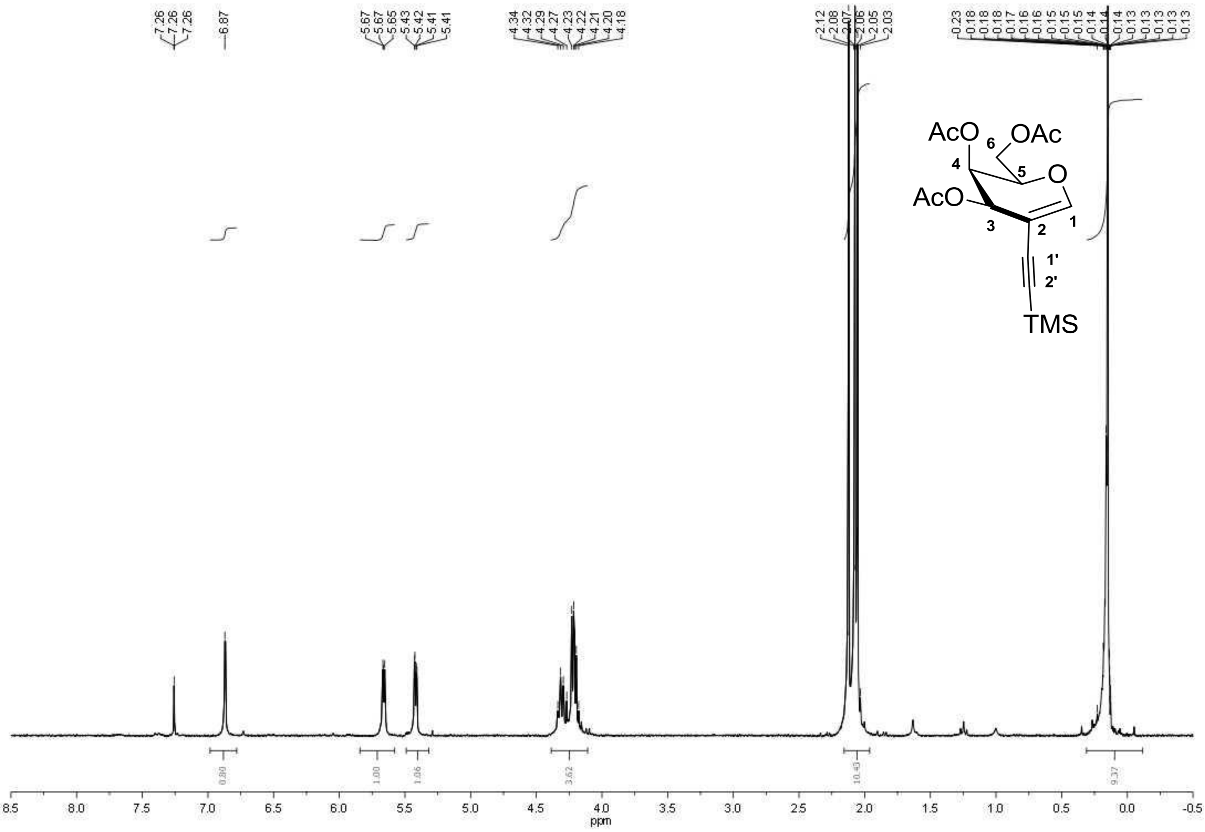

^{1}H NMR (300 MHz, CDCl$_3$)

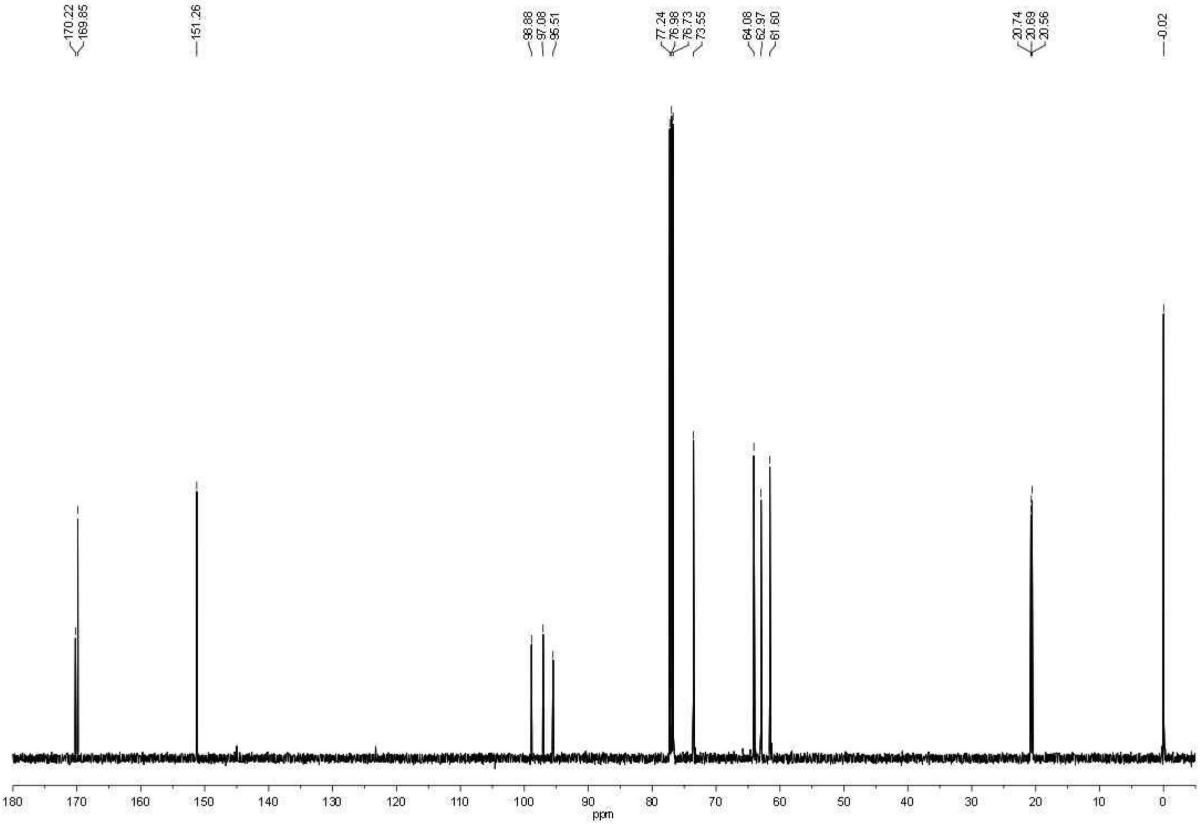

^{13}C NMR (125 MHz, CDCl$_3$)

1-Phenylethynyl-3,4,6-tri-*O*-benzyl-2-chloroglucal (13)

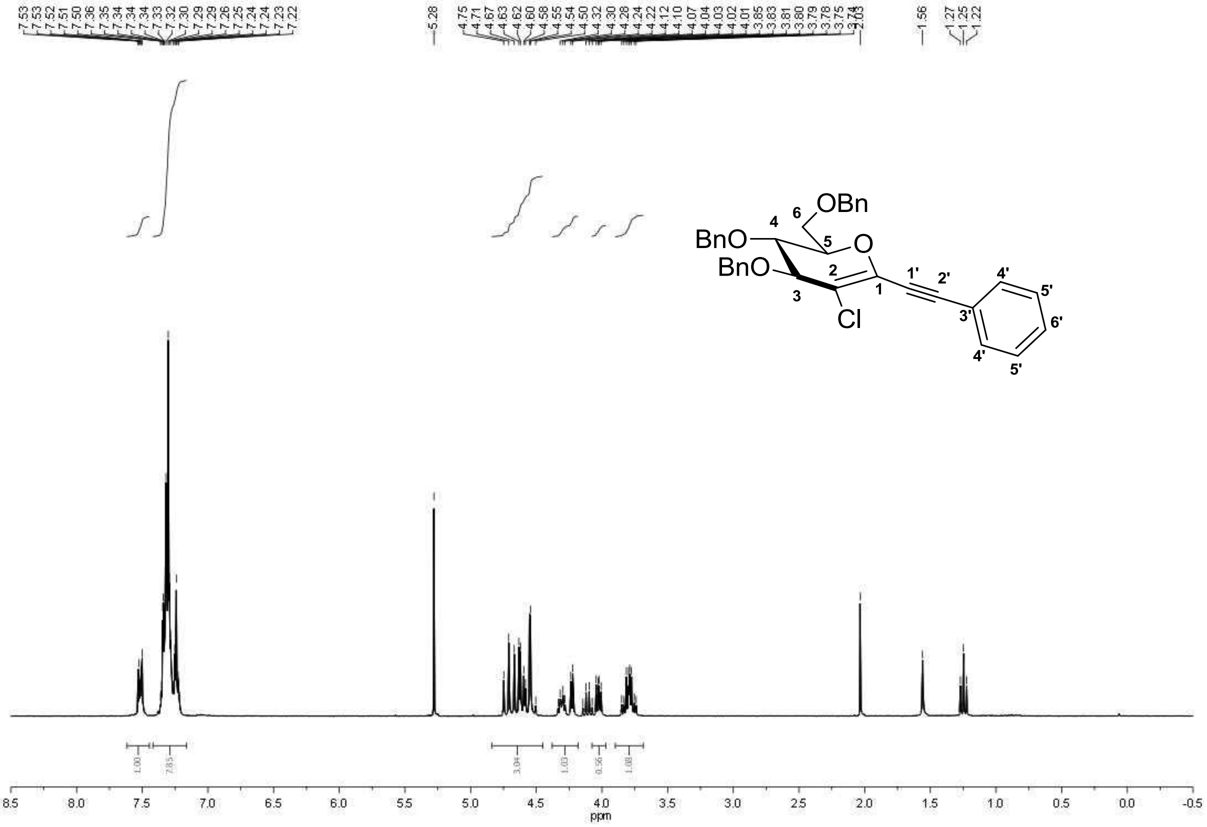

^{1}H NMR (300 MHz, $CDCl_3$)

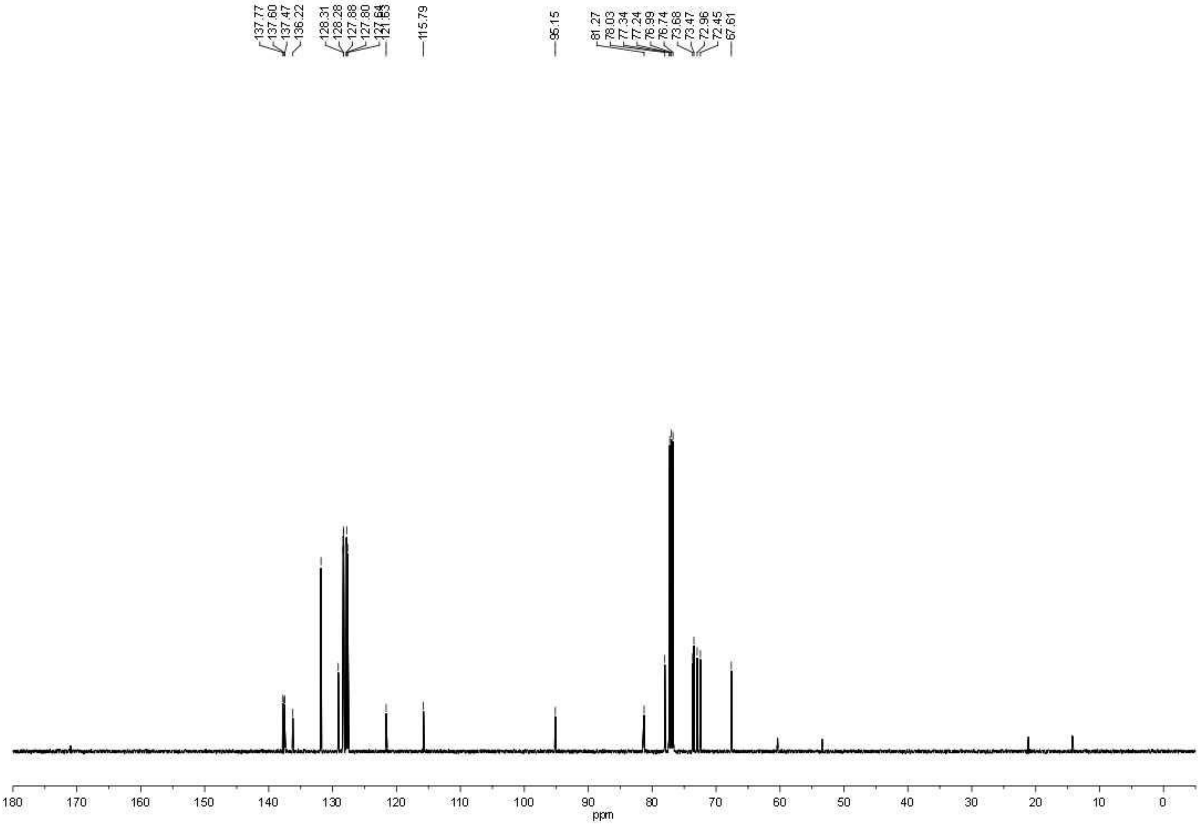

^{13}C NMR (125 MHz, $CDCl_3$)

1-(2-(3-Methylphenyl)ethyl)-3,4,6-tri-*O*-isopropylsilylglucal (14a)

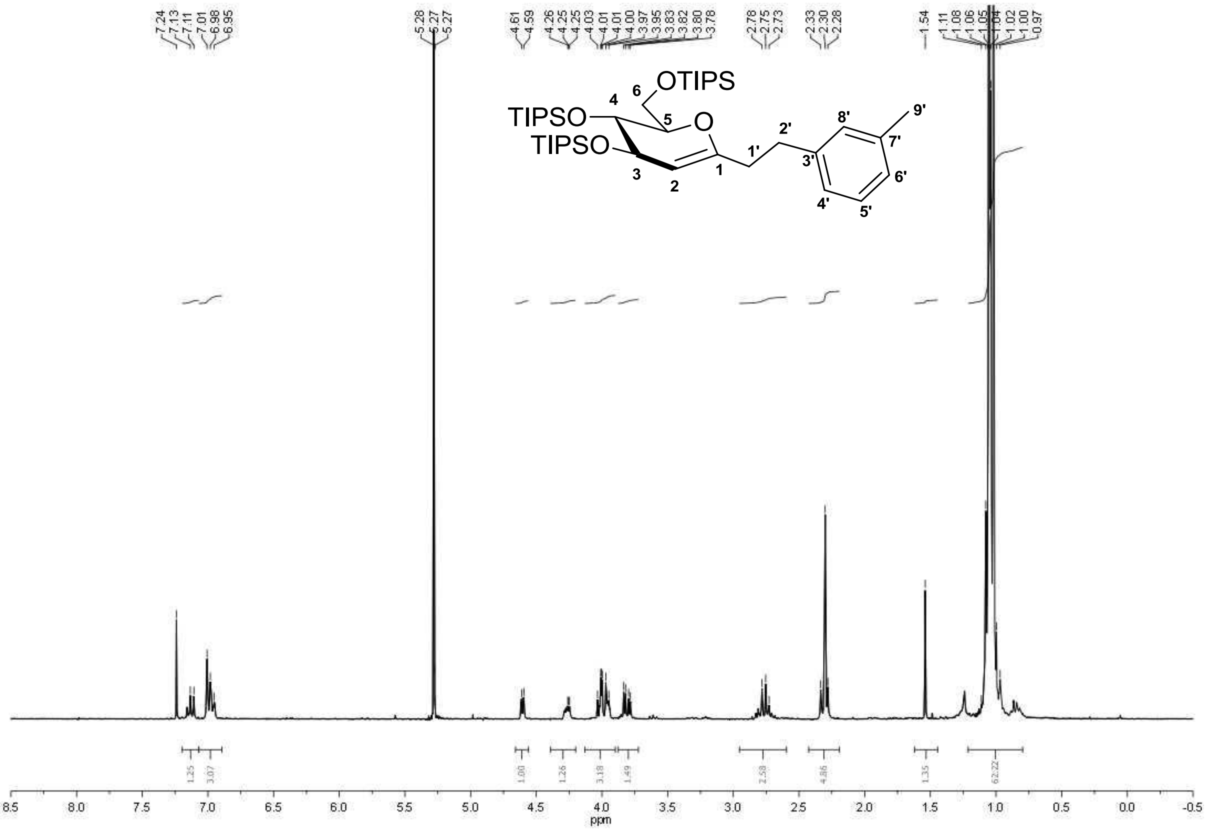

^{1}H NMR (300 MHz, $CDCl_3$)

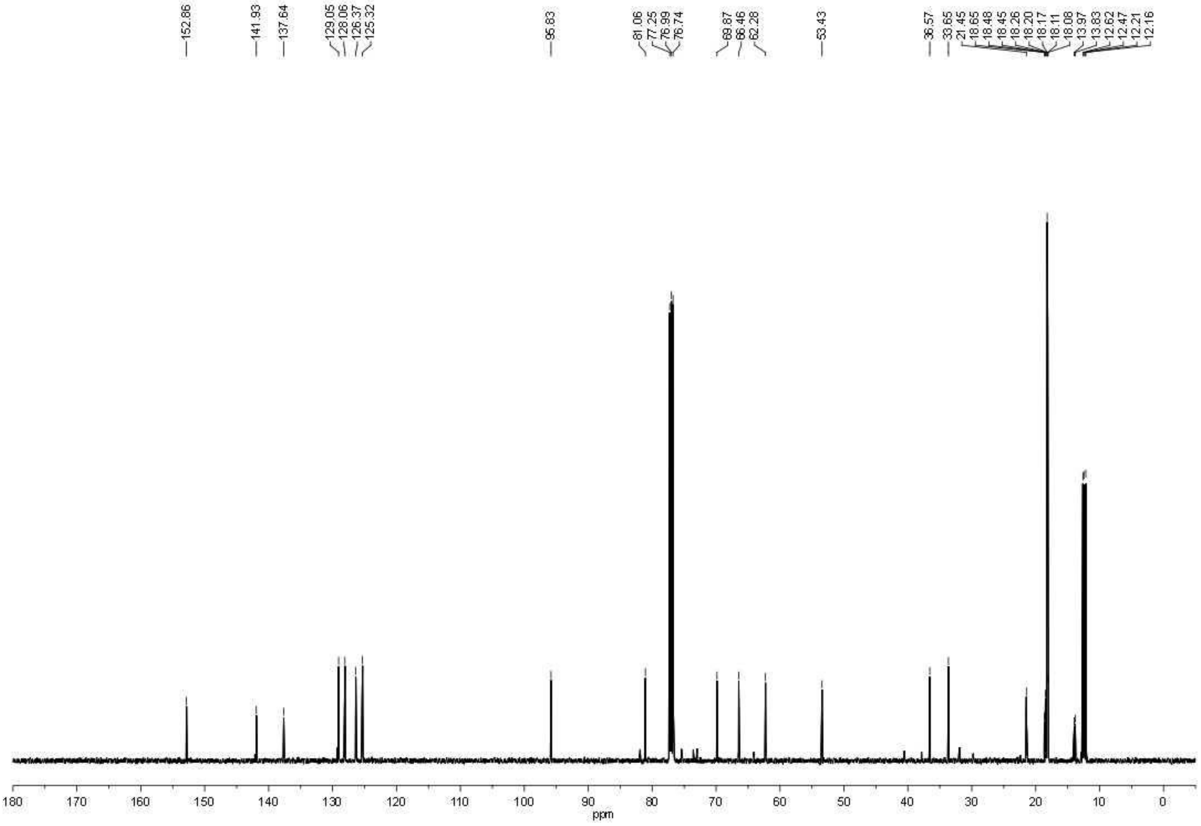

^{13}C NMR (125 MHz, $CDCl_3$)

1-(2-(3-Methylphenyl)ethyl)-3,4,6-tri-*O*-benzylglucal (14b)

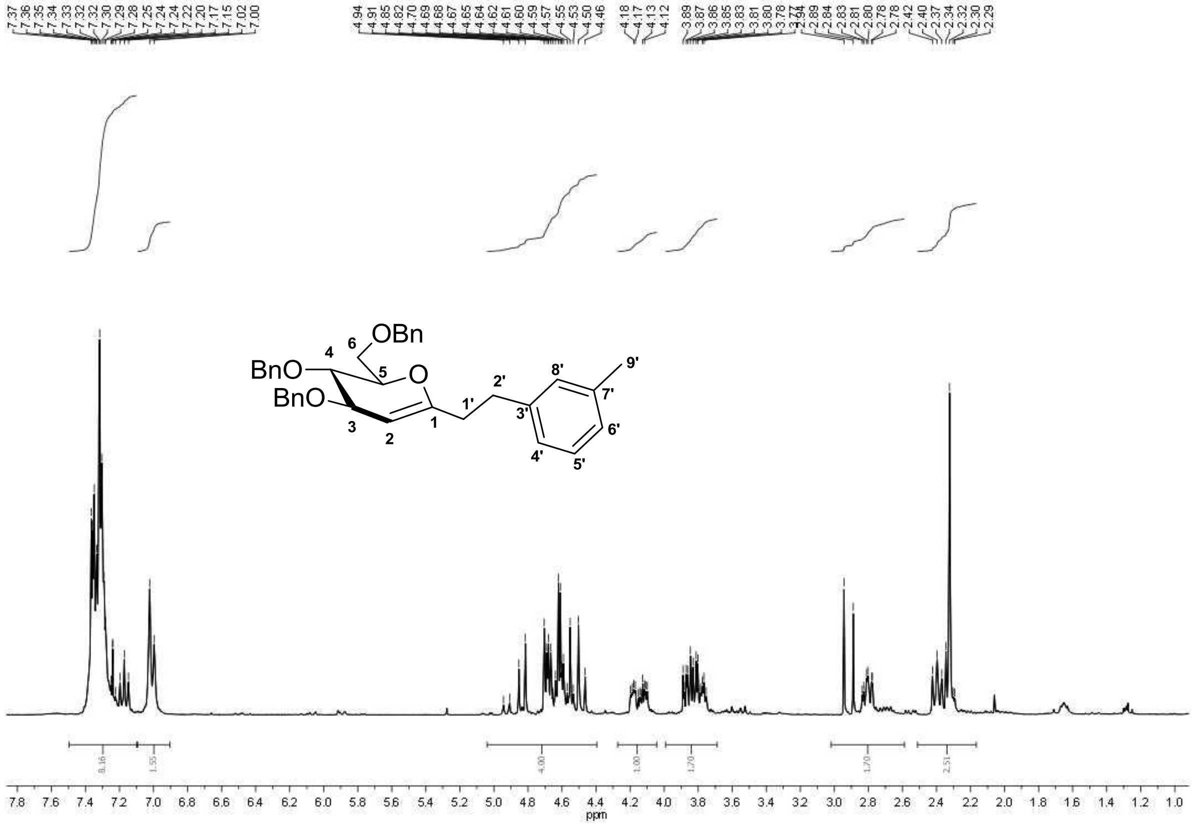

^{1}H NMR (300 MHz, CDCl$_3$)

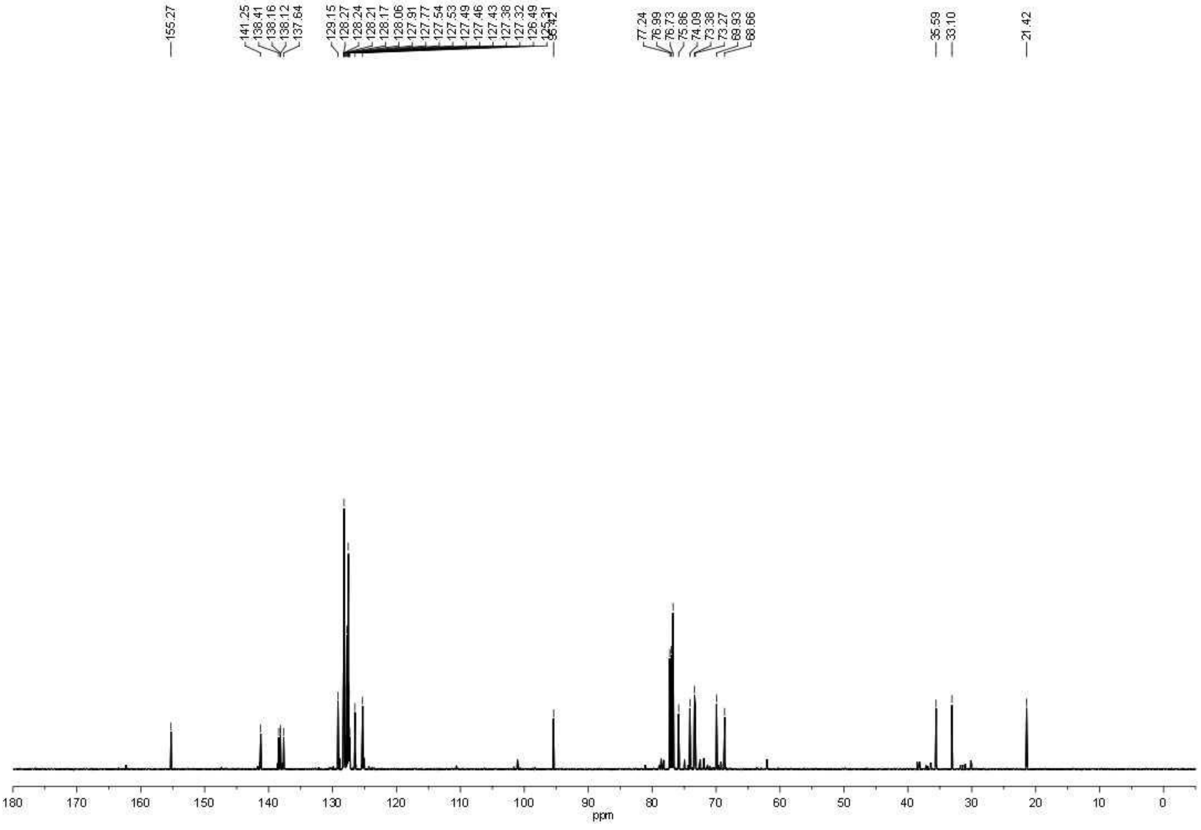

^{13}C NMR (125 MHz, CDCl$_3$)

1-Pentyl-3,4,6-tri-*O*-isopropylsilylglucal (14c)

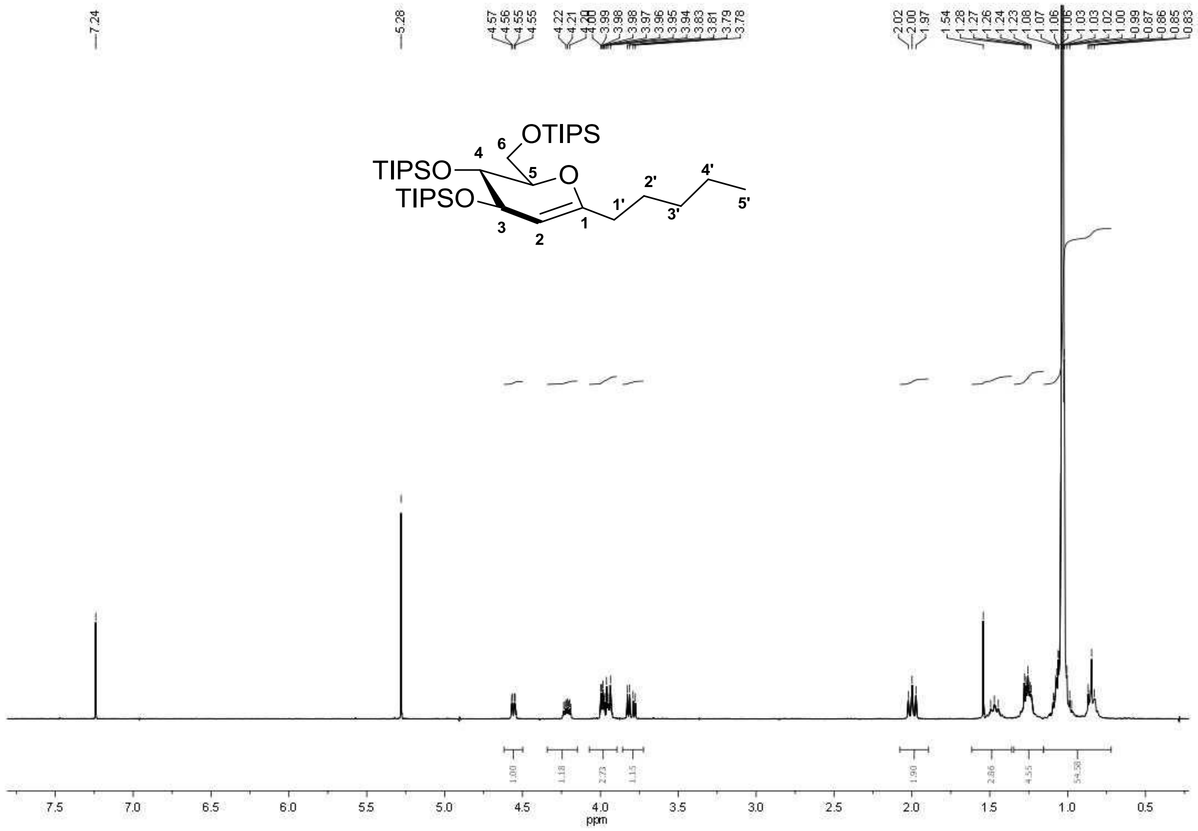

^{1}H NMR (300 MHz, $CDCl_3$)

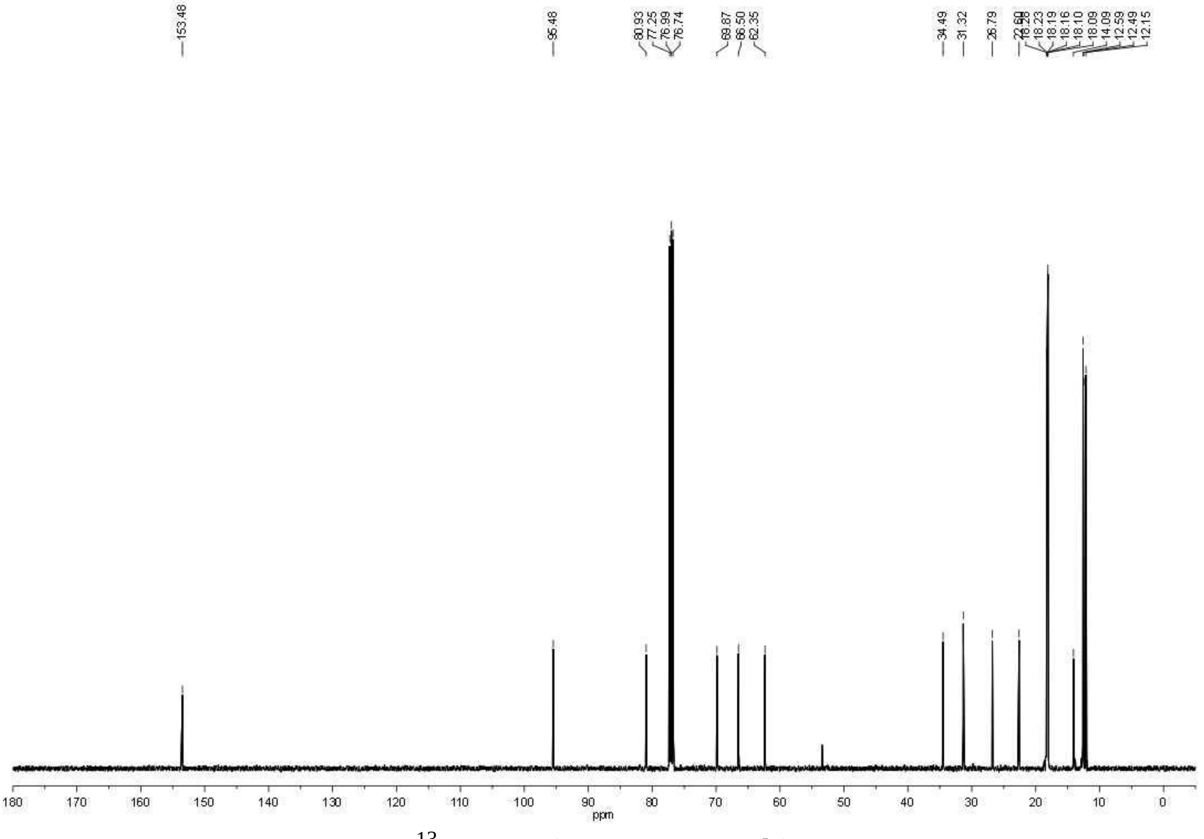

^{13}C NMR (125 MHz, $CDCl_3$)

1-(2-(3-Methyphenyl)ethyl)glucal (14aa)

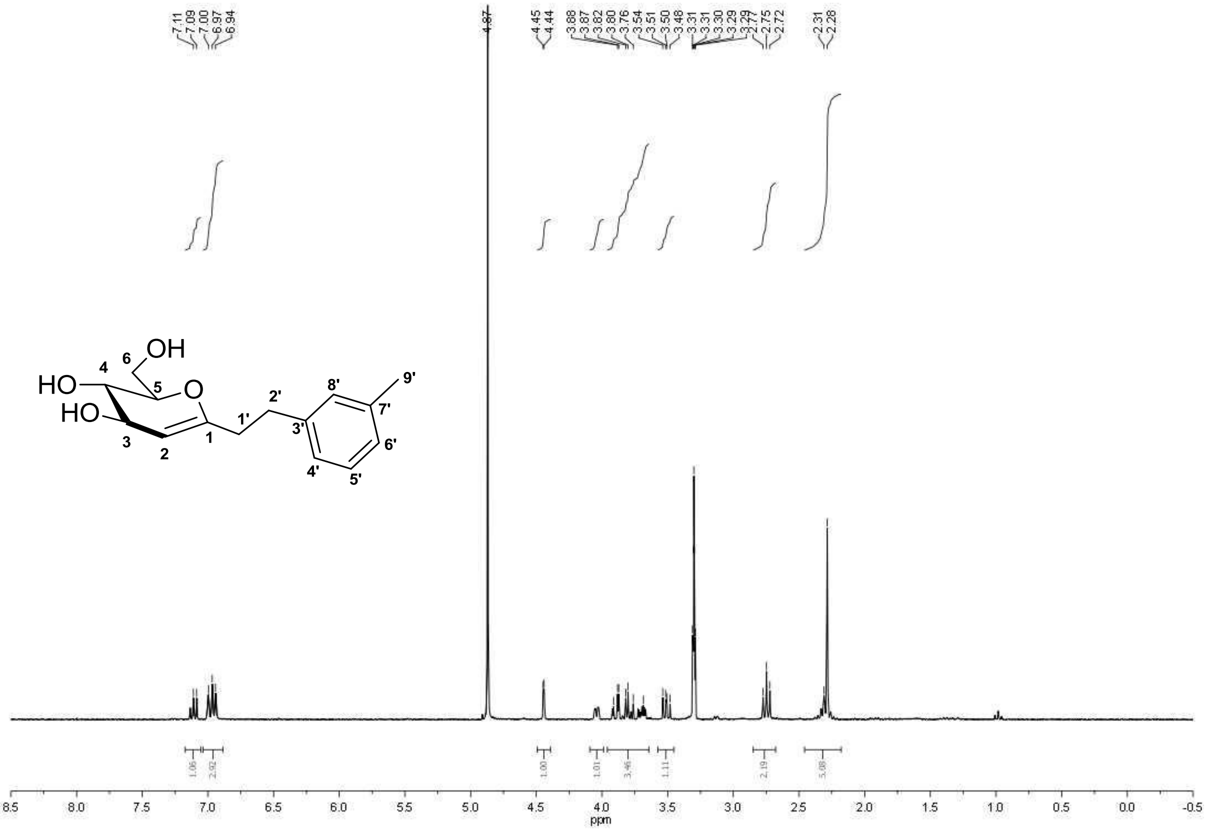

^{1}H NMR (300 MHz, $CDCl_3$)

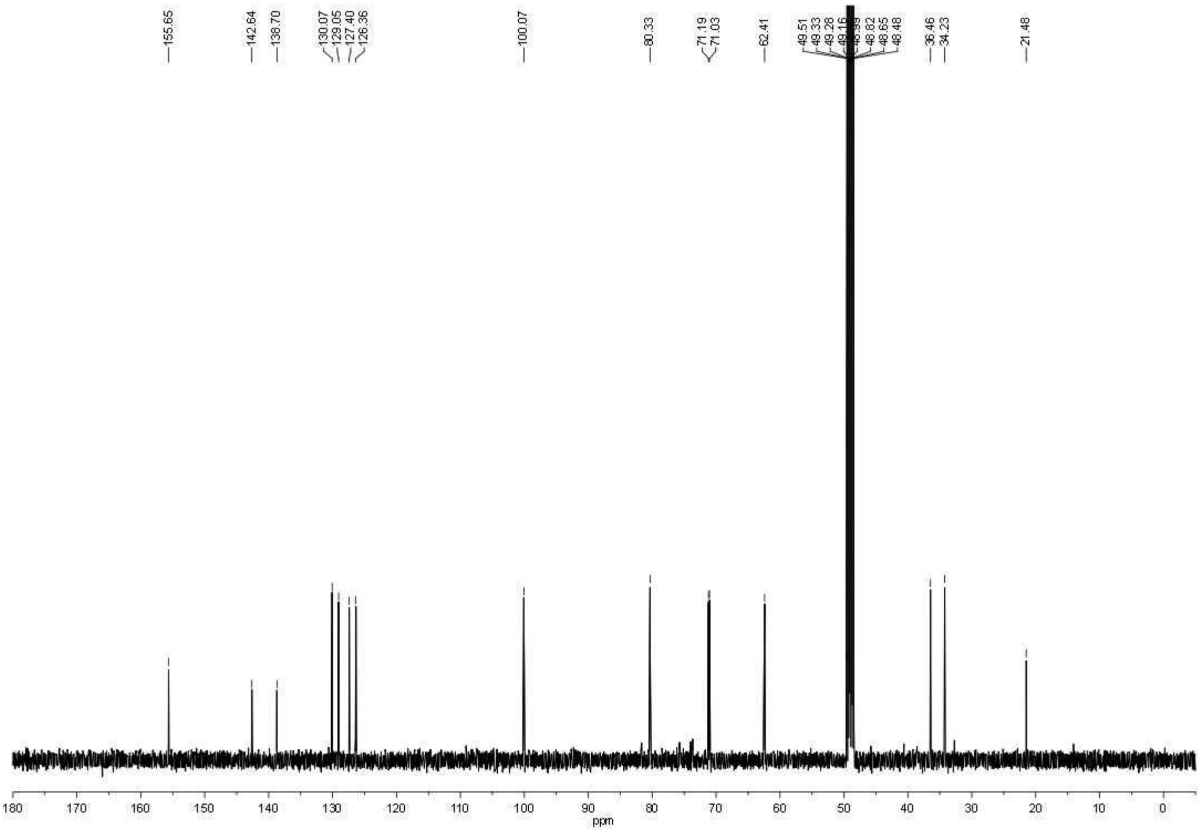

^{13}C NMR (125 MHz, $CDCl_3$)

3,4,6-Tri-*O*-acetyl-1,2-dideoxy-2-(*R*)-(2-phenylethyl)galactose (14d)

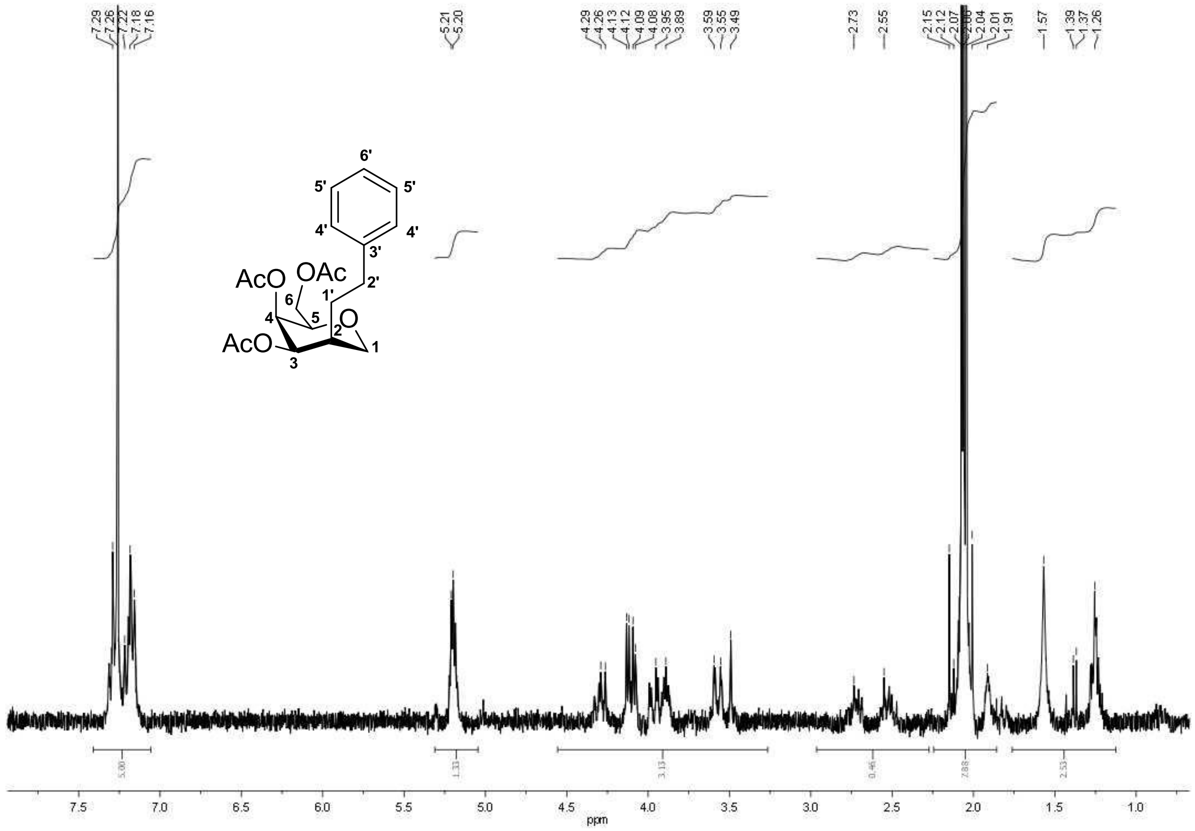

^{1}H NMR (300 MHz, $CDCl_3$)

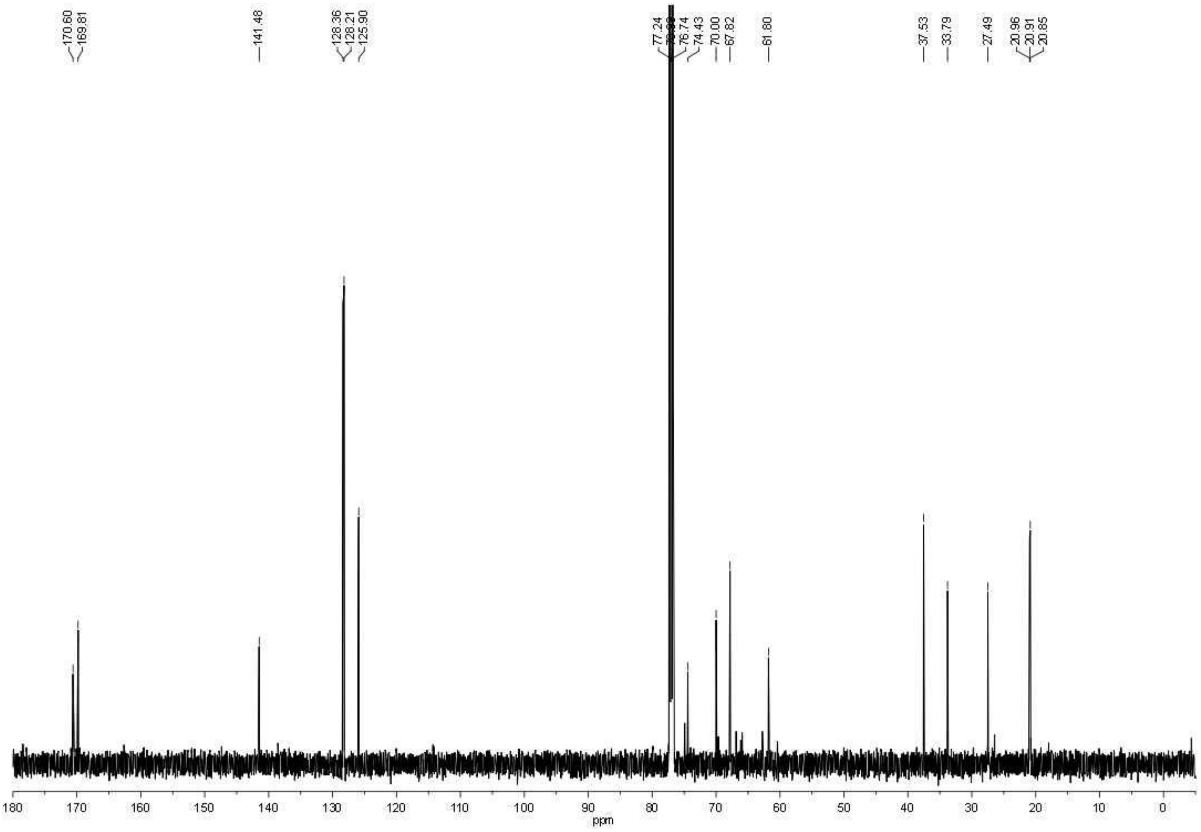

^{13}C NMR (125 MHz, $CDCl_3$)

2-(2-Trimethylsilylethyl)-3,4,6-tri-*O*-acetylgalactal (14e)

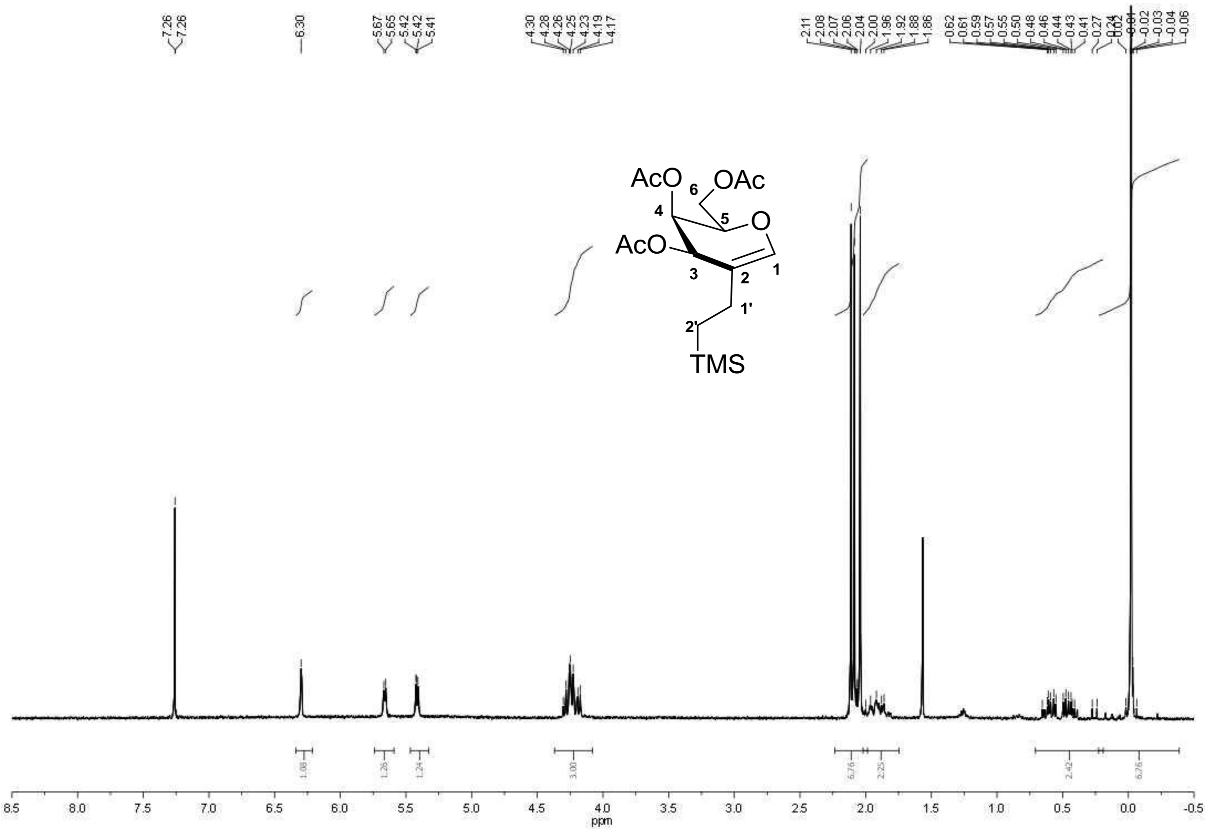

^{1}H NMR (300 MHz, $CDCl_3$)

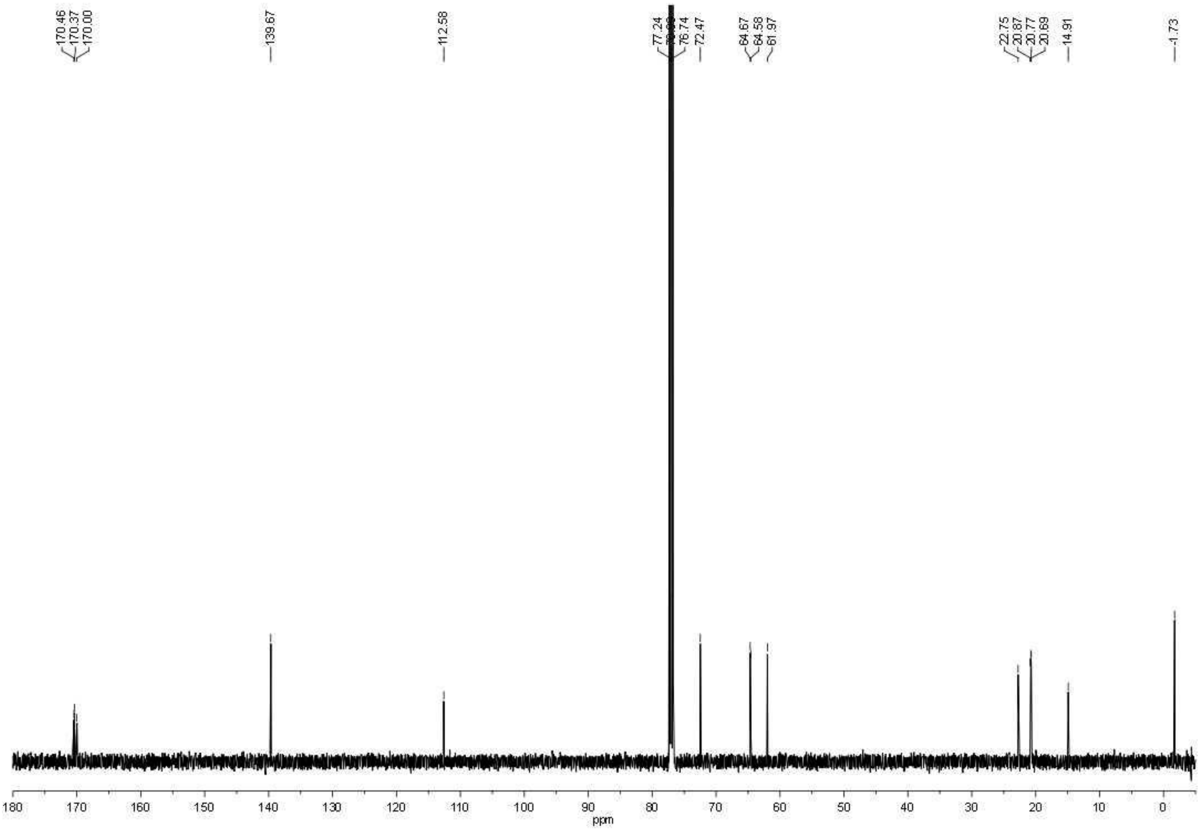

^{13}C NMR (125 MHz, $CDCl_3$)

α-1-(2-(3-Methylphenyl)ethyl)-1-deoxy-3,4,6-tri-*O*-benzylglucose (15a)

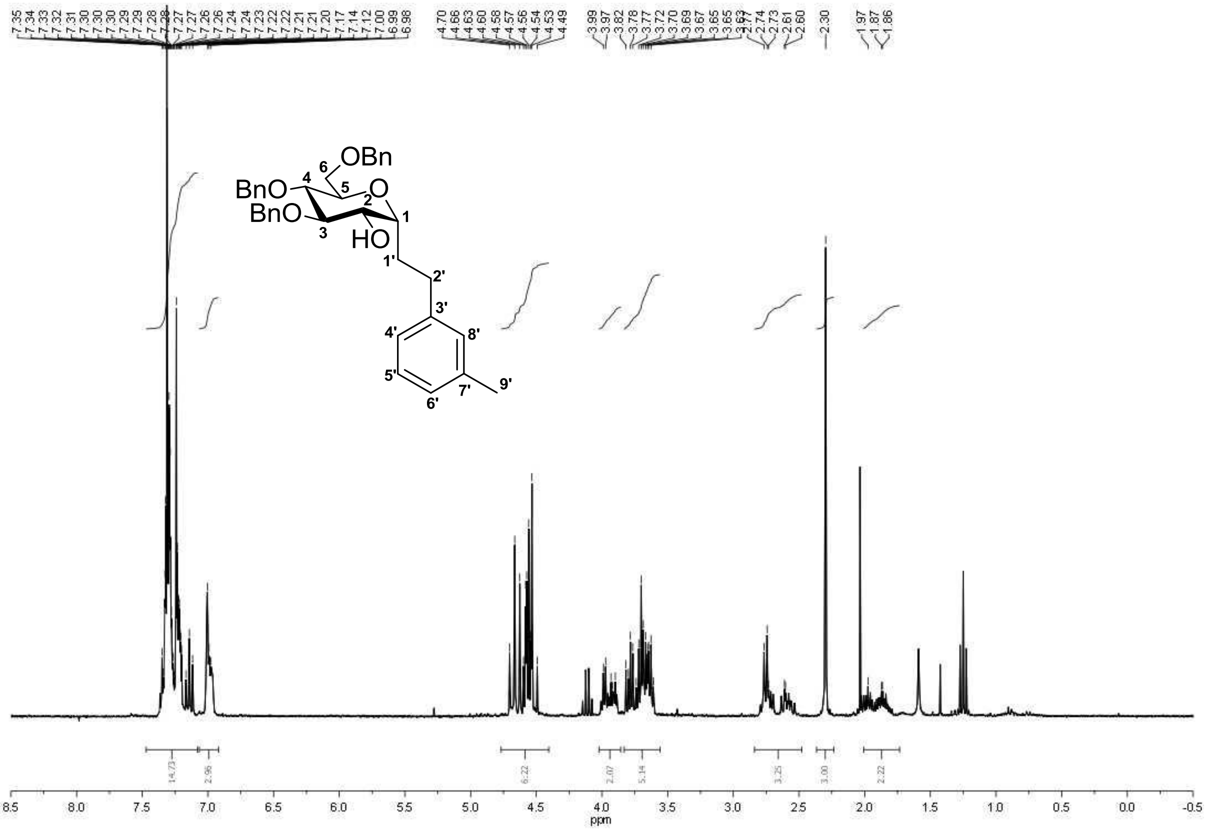

^{1}H NMR (300 MHz, $CDCl_3$)

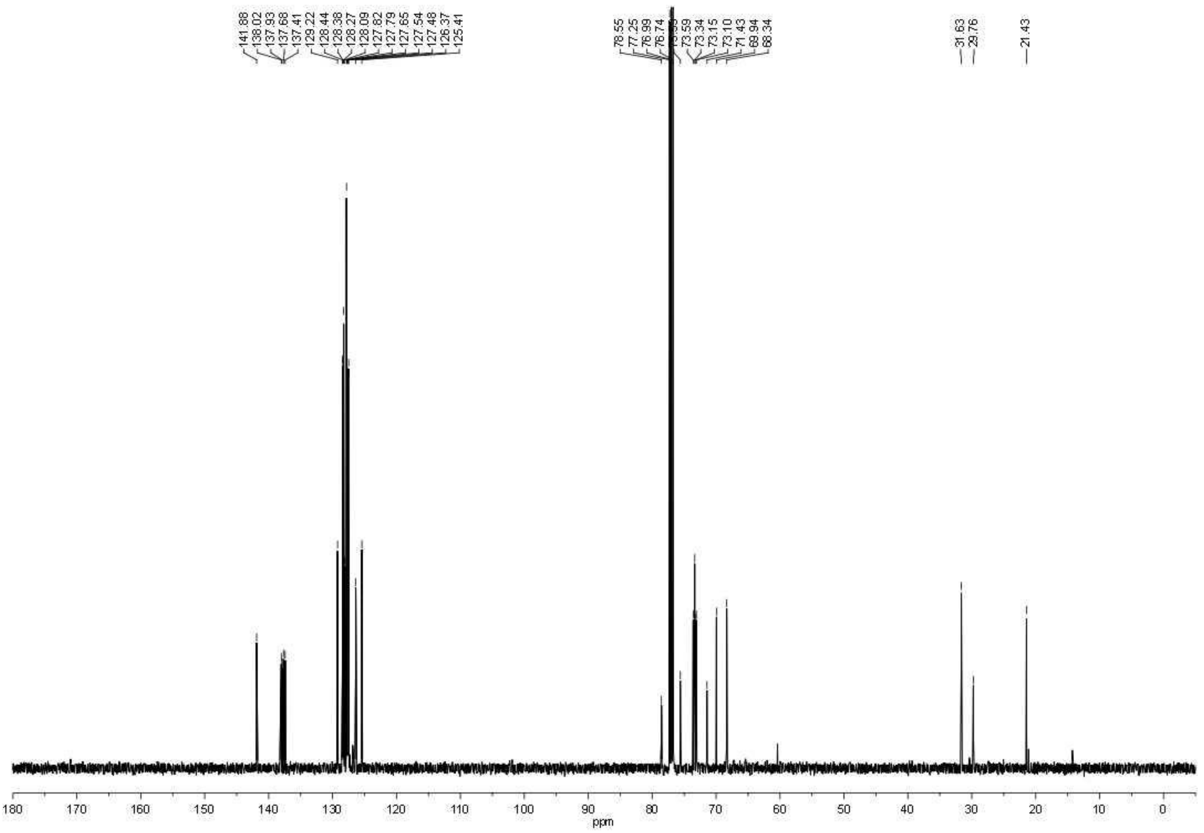

^{13}C NMR (125 MHz, $CDCl_3$)

β-1-(2-(3-Methylphenyl)ethyl)-1-deoxy-3,4,6-tri-*O*-benzylglucose (15b)

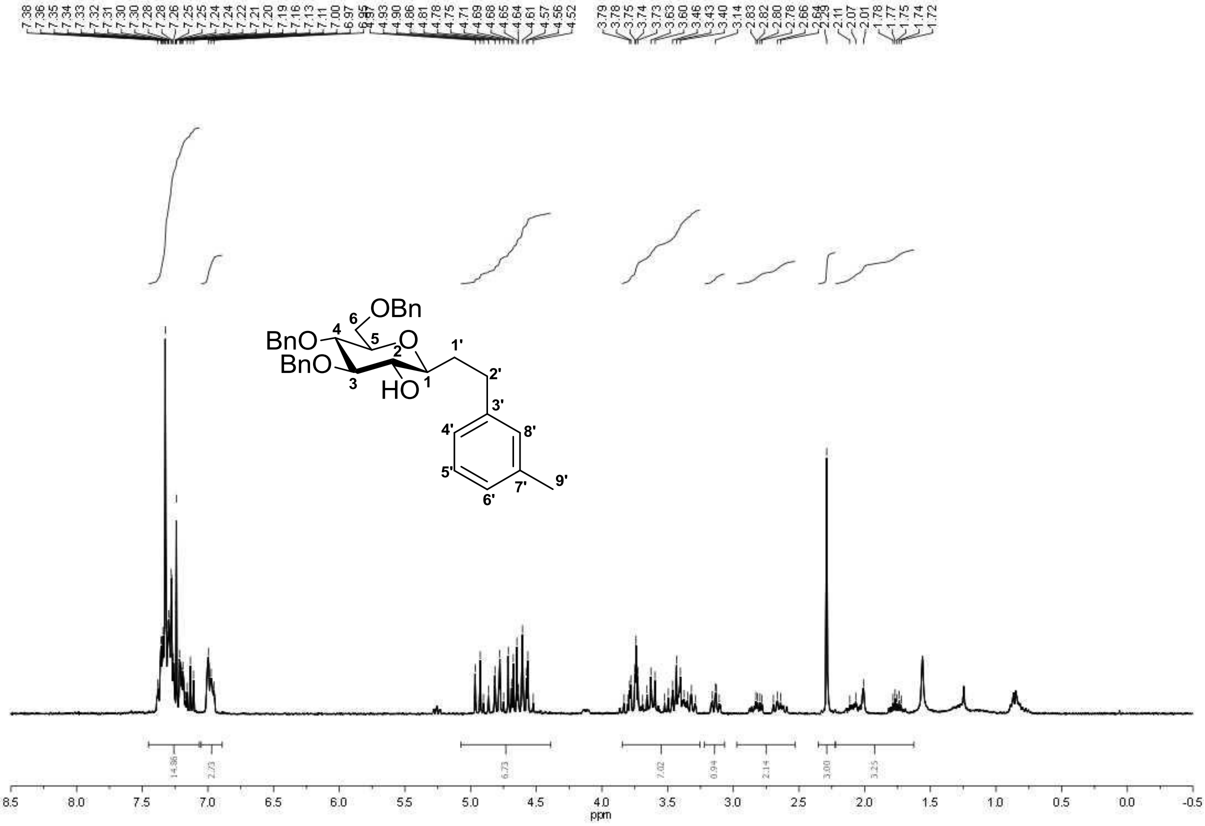

^{1}H NMR (300 MHz, $CDCl_3$)

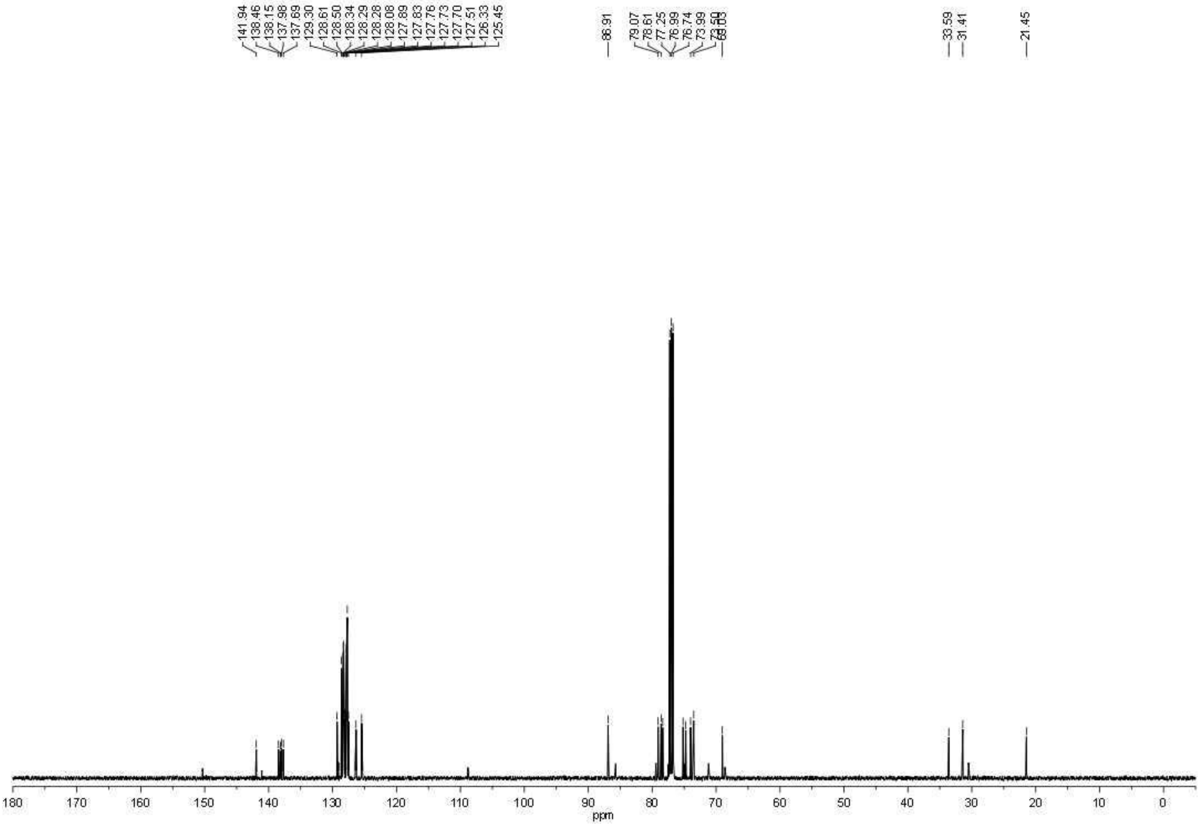

^{13}C NMR (125 MHz, $CDCl_3$)

β-1-Pentyl-3,4,6-tri-*O*-isopropylsilylglucose (15c)

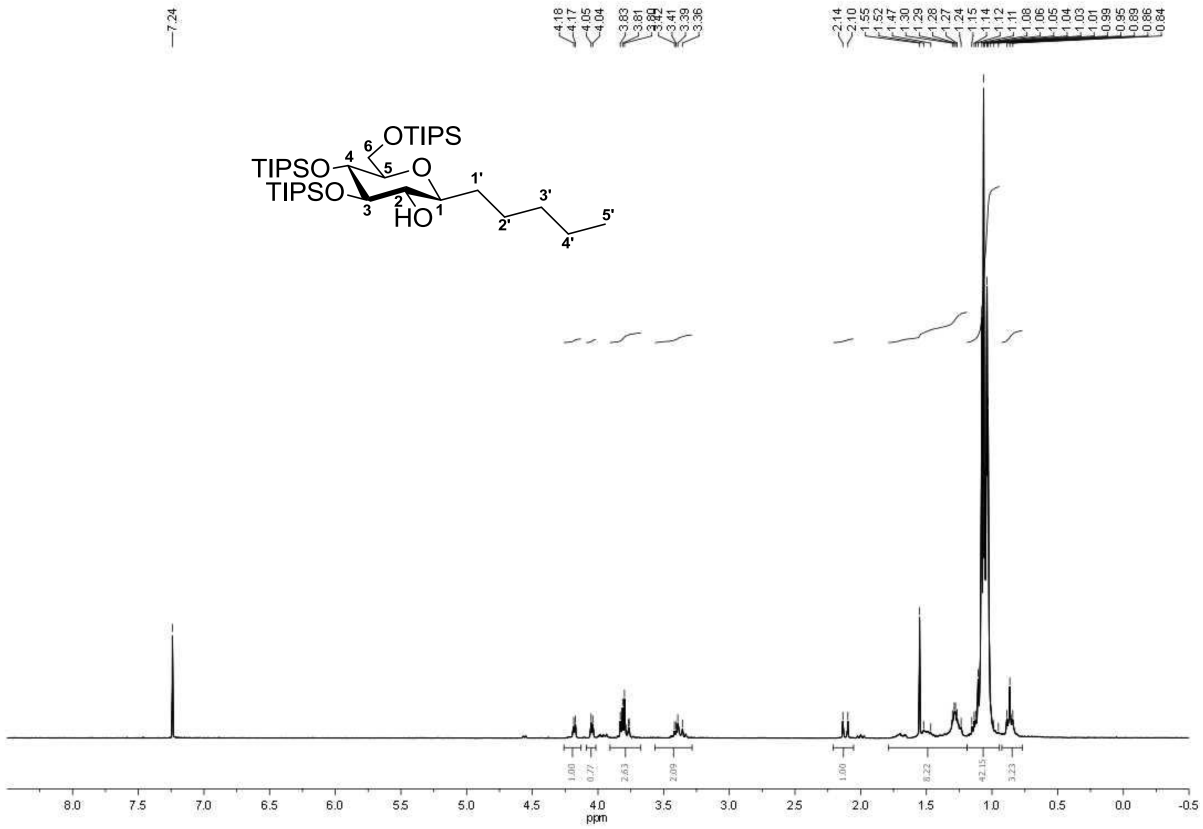

^{1}H NMR (300 MHz, $CDCl_3$)

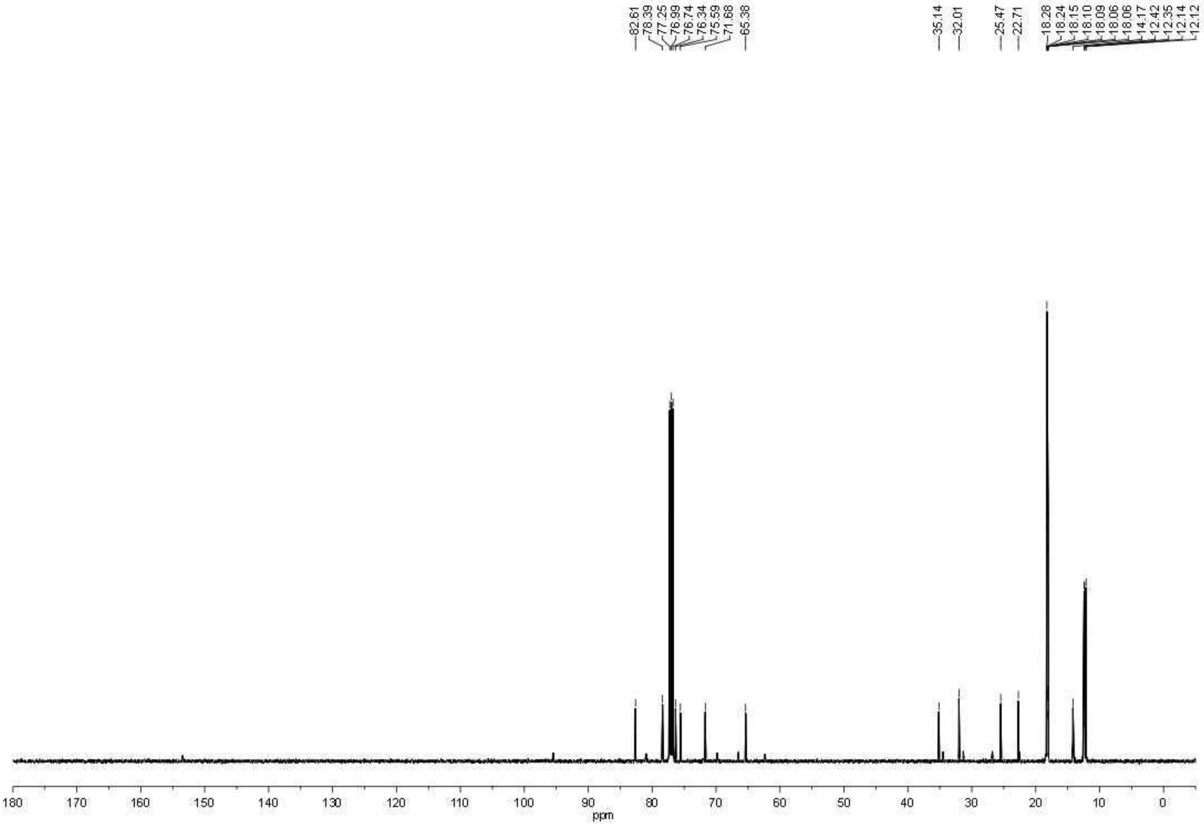

^{13}C NMR (125 MHz, $CDCl_3$)
